# Simulation of the Hydro-ecological Impacts of Climate Change on an Upland Peatland in the Massif Central

**DOI:** 10.1007/s13157-026-02046-7

**Published:** 2026-03-23

**Authors:** Julian Richard Thompson, Arnaud Duranel, Emma Keisser, Philippe Durepaire, Hervé Cubizolle

**Affiliations:** 1 Department of Geography, University College London (UCL), London, WC1E 6BT UK; 2 Jean Monnet University, UMR 5600 CNRS EVS, Saint-Etienne CEDEX 2, 42023 France; 3 Réserve Naturelle Nationale de la Tourbière des Dauges, Conservatoire d’Espaces Naturels de Nouvelle Aquitaine, Sauvagnac, Saint-Léger-la- Montagne, 87340 France

**Keywords:** Climate change, Peatlands, Massif Central, MIKE SHE, DRIAS-2020

## Abstract

Hydro-ecological impacts of 60 climate change scenarios on peat ecosystems of the Dauges National Nature Reserve are assessed using high spatial resolution MIKE SHE / MIKE 11 modelling. Annual precipitation increases for 45 of 60 scenarios (ensemble mean increases: 5–8% for RCP2.6, 1–5% for RCP8.5). Annual potential evapotranspiration increases in all cases (ensemble mean: 2–3% for RCP2.6, 6–12% for RCP8.5). Winters become wetter and summers drier. Mean stream discharges increase in most cases (ensemble means for catchment outlet: 8–9% for RCP2.6, 2–4% (2050s) and − 1–2% (2080s) for RCP8.5). Flows become more seasonal with increasing peaks and declining lows. Winter peat groundwater levels still intercept the surface but declines in summer dominate (> 80% of cases) driving enhanced seasonal ranges and lower mean levels (> 70% of cases). The magnitude of changes increases with higher radiative forcing and into the future (ensemble mean increases in low levels of <1 cm for RCP2.6 in both time slices, declines of 5–7 cm and 12–14 cm for RCP8.5 in the 2050s and 2080s, respectively). The largest declines are concentrated around peatland margins. Hydrological conditions assumed to support mire vegetation decline in extent in most cases (41 of 60 scenarios, more common with higher radiative forcing). Small (< 1%) increases in area are projected by ensemble means for RCP2.6 with declines of 12–13% for RCP8.5 in the 2080s. Sources of uncertainty include shifts in catchment vegetation, changes in peat hydraulic properties and mire vegetation species-specific impacts of hydrological changes.

**Supplementary Information:**

The online version contains supplementary material available at 10.1007/s13157-026-02046-7.

## Introduction

Hydrology exerts dominant controls upon wetland ecosystem functioning (e.g. Baker et al. 2009) and underpins delivery of multiple ecosystem services (Maltby et al. 2011; Okruszko et al. 2011). Water table depth and its temporal variability strongly influence the composition and zonation of wetland plants (Toogood et al. 2008; Wheeler et al. 2009) whilst hydrological conditions influence suitability and quality of habitat for a range of animals contributing to the conservation significance of many wetlands (Ausden et al. 2001; Plum 2005; Smart et al. 2008; Eglington et al. 2010; House et al. 2016a).

The reliance upon specific hydrological conditions for the creation and persistence of wetlands and their plant communities is typified by mires; wetlands with naturally accumulated peat that are dominated by vegetation currently producing peat (Joosten and Clarke 2002). High water tables, driven by excess of precipitation over evapotranspiration as well as groundwater inflows that are critical for many mires (Boeye and Verheyen 1992; Rossi et al. 2012; House et al. 2016b), drive saturation at or very close to the ground surface. Anoxic conditions promote accumulation of plant remains from which the peat is derived (Rydin and Jeglum 2006). Hydrological variations, alongside base richness, pH and fertility gradients, drive mire floristic gradients (e.g. Anderson et al. 1995; Wheeler and Shaw 1995a, b; Wheeler and Proctor 2000; Marini et al. 2008; Sottocornola et al. 2008). The water level regime often dominates over other environmental factors in delimiting extent of mire vegetation and in explaining vegetation patterns (Asada 2002; Yazaki et al. 2006; Conradi and Friedmann 2013; Hettenbergerová et al. 2013). This control operates via factors that include relationships between water level and root zone aeration/drought stress (Gowing et al. 1998, 2002a; Silvertown et al. 1999; Dwire et al. 2006; Clilverd et al. 2022). Water levels also influence physico-chemical processes that impact nutrient availability whilst water flow through the root zone, sometimes driven by groundwater influx from the wider catchment, produces higher redox potential compared to more stagnant conditions (Armstrong and Boatman 1967) and increases nutrient supply.

Climate change-driven alterations to hydrological conditions may impact the long-term persistence and health of mires. Intensification of the global hydrological cycle will impact precipitation and evapotranspiration (IPCC 2018; Douville et al. 2021; Lee et al. 2021), driving changes in catchment hydrology. Modifications to stream flow, soil moisture as well as groundwater recharge, levels and flow can be expected (Kundzewicz et al. 2007; Bates et al. 2008; Douville et al. 2021; Jiménez Cisneros et al. 2014). There is considerable uncertainty in these hydrological changes (Wilby and Dessai 2010) that includes variable responses in different regions (Gosling et al. 2017; Do et al. 2020) and across individual catchments (Thompson et al. 2014, 2017a), as well as different magnitudes and even directions of change in high and low flows/water levels (Giuntoli et al. 2015; Chan et al. 2020). Such changes have potentially major implications for the world’s aquatic ecosystems (Döll and Zhang 2010; Thompson et al. 2021a; Parmesan et al. 2022). Climate change is thus a significant causative factor that may drive future wetland loss and degradation (Ramsar Convention on Wetlands 2018, 2021; Xi et al. 2020). A particular concern for mires are declines in the area with suitable conditions for peat development, especially towards limits of current distributions (Gallego-Sala et al. 2010; Coll et al. 2014). Whilst climate change may not result in rapid, wholesale mire loss (Gallego-Sala and Prentice 2013), it will likely impose stress to environments that, alongside many aquatic ecosystems (Tickner et al. 2020), have already been impacted by human activities. For mires, these include direct drainage, peat extraction and forestry (e.g. Joosten and Clarke 2002; Parish et al. 2008). A particular concern is potential positive feedback upon global warming if large carbon stores provided by peat-based wetlands are diminished due to climate change-induced hydrological impacts (e.g. Frolking et al. 2011).

Evaluating hydrological impacts of climate change upon mires, or other freshwater wetlands, and translating these impacts to ecosystem responses such as persistence of specific vegetation requires a combination of approaches. The first requires process-based hydrological/hydraulic models which can represent complex, inter-related processes within wetlands and their catchments. Process representation should be at sufficiently fine spatial and vertical resolutions to accommodate variability in hydrological conditions common within even the smallest wetlands (Thompson et al. 2004; House et al. 2016b). This inevitably imposes data demands which can restrict application of the most robust modelling approaches in the absence of intensive field surveys and monitoring (Hollis and Thompson 1998). High resolution modelling is required to compare simulated hydrological conditions against the often very sensitive preferences of wetland plants and animals (Thompson et al. 2009, 2017b, 2023; House et al. 2016b; Clilverd et al. 2022). These preferences are themselves an important prerequisite for hydro-ecological impact assessments and are usually based on field investigations to establish relationships between hydrological characteristics (e.g. water table depth, surface wetness) and species distributions (Ausden et al. 2001; Gowing et al. 2002b; Tattersfield and McInnes 2003; Wheeler et al. 2004).

Simulating climate change impacts requires forcing of meteorological inputs with climate projections. Ensembles of future climate facilitate investigation of uncertainty due to factors that include alternative emissions scenarios, future periods or General Circulation Models (GCM; e.g. Wilby and Dessai 2010; Gosling et al. 2017). Whilst forcing of meteorological inputs to hydrological models that incorporate wetland components with GCM projections can be undertaken, the spatial resolution of GCMs means that this is most appropriate for large catchments with extensive wetlands (e.g. Thompson et al. 2016, 2017a). For smaller wetlands, downscaling of GCM projections to smaller spatial scales is desirable although it represents another source of uncertainty. Downscaled projections for specific countries (e.g. Jenkins et al. 2009; Lowe et al. 2018 Météo-France 2020) provide datasets that are specifically designed to support climate change impact assessments. They provide a means of reconciling disparities in the spatial resolution at which GCMs operate, and the much finer resolutions required for robust investigations of the impacts of climate change on most wetlands (e.g. Thompson et al. 2009, 2023; House et al. 2016a, 2017).

The current study employs a previously described (Duranel 2015; Duranel et al. 2021) high resolution MIKE SHE/MIKE 11 hydrological/hydraulic model of a catchment in the French Massif Central containing acid mire habitats. A total of 76 climate change scenarios are developed using the DRIAS-2020 dataset (Donner accès aux scénarios climatiques Régionalisés français pour l’Impact et l’Adaptation de nos Sociétés et environnement; www.drias-climat.fr; Météo-France 2020) which provides nation-wide high-resolution projections for a range of meteorological variables. Scenarios encompass three alternative emissions scenarios with impacts projected to the 2050 s and 2080s. To our knowledge, this is the first study to employ the complete set of DRIAS-2020 scenarios within a wetland climate change impact assessment. Initial investigation of the hydrological impacts of climate change focusses on stream discharge within and from the catchment and peat groundwater levels. A further novel aspect of the study is an evaluation of the future extent of peat-forming wetlands. This is achieved using relationships between simulated hydrological conditions and the distribution of mire vegetation and peat soils.

## Methods

### Study Area

**Fig. 1:**
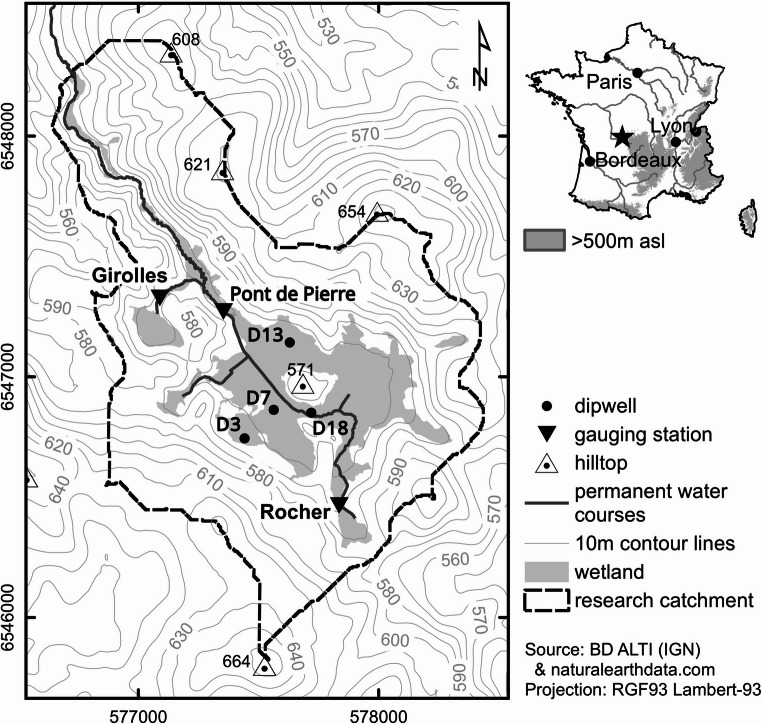
The Dauges mire, its catchment and locations of hydrological monitoring infrastructure (gauging stations and dip wells) referred to in the current study (see Duranel et al. 2021 for the complete monitoring network used for model calibration and validation)

The MIKE SHE/MIKE 11 model domain encompasses a 2.31 km^2^ catchment that approximates the boundaries of the Dauges National Nature Reserve (NNR) located within the Haute-Vienne administrative department of Nouvelle-Aquitaine, France (Fig. 1). It is close to the centre of the Monts d’Ambazac, a low altitude mountain range located towards the northwestern margins of the Massif Central. Elevation varies from 664 m above sea level (masl; NGF69) at the summit of Puy de la Garde in the southeast to 532 masl at the catchment outlet (Duranel et al. 2021). Catchment topography comprises a circus-like basin with a relatively flat bottom surrounded by gentle hills which opens into a narrow linear valley. This is characteristic of the etch-basins (alvéoles in French) that are common in Hercynian mountains (Valadas 1998). A small residual hill, Puy Rond, rises approximately 30 m above the main basin whilst downstream, beyond the model domain, the valley flows into a second etch-basin. Drainage is via a single stream which flows through the mire to the south of Puy Rond and is supplemented by a number of small tributaries. Ultimately, the stream joins with others draining similar catchments to form the Couze River which in turn enters the Gartempe River and then the Vienne River, a major tributary of the lower Loire.

Catchment geology comprises leucogranite dissected by numerous veins of lamprophyres. Erosion has almost entirely removed a layer of saprolite above the leucogranite and it is generally less than 1–2 m thick (Valadas 1998; Mauroux et al. 2009; Duranel 2015). Peat, which supports acidic mire habitats, is primarily located at the bottom of the etch-basin. Based on botanical (Durepaire and Guerbaa 2008) and pedological (Duranel 2015) criteria, the area defined as mire covers 0.43 km^2^ (19% of the catchment; Fig. 1). Mean peat depth is 0.80 ± 0.49 m whilst the maximum depth towards the centre of the mire is 3.45 m (Duranel 2015).

The Dauges catchment lies at the transition between altered ocean and mountainous climates (Joly et al. 2010). Long-term (1981–2010) mean temperature and annual total precipitation for the Saint-Léger-la-Montagne meteorological station 4 km from the site and at an altitude of 629 masl are 10.1 °C and 1367 mm, respectively. The equivalent figures for a slightly shorter period (1998–2013) for a station in the middle of the mire are 9.6 °C and 1304 mm, respectively. Annual total Penman Monteith reference evapotranspiration in the middle of the mire (given here as a standardised estimate of atmospheric evaporative demand independent of vegetation type to allow for comparison with other sites in different geographical contexts) is 687 mm. Precipitation is relatively well distributed throughout the year albeit with a winter peak (middle of the mire in December: 151 mm) and summer low (June and August: 79 mm). Evapotranspiration is more variable through the year and follows the reverse seasonal distribution (e.g. December 12 mm vs. July 112 mm).

Semi-natural beach (*Fagus sylvatica* L.), oak (*Quercus robur* L.) and chestnut (*Castanea sativa* Mill.) woodland dominate the catchment although there are patches of permanent acidic grassland and heathland. There are also a few coniferous plantations (largely Douglas fir, *Pseudotsuga menziesii* (Mirbel) Franco, with some Scots pine, *Pinus sylvestris* L.) but their extent is limited. Wet woodland dominated by willow (*Salix* spp.) and birch (*Betula* spp.) is scattered within the mire and around its margins. Based on their floristic composition, a large part of the mire was classified by Durepaire and Guerbaa (2008) as belonging to “raised bogs” as defined in the Corine Biotope classification (Commission of the European Communities 1991). However, the mostly concave topography indicates that the mire is not ombrotrophic, even though some microforms may be. The original NNR designation in 1998 was driven by the need to conserve the acid mire habitats not least because French mires are towards the southern limit of these peat forming habitats in the northern hemisphere (Julve 1996). The ecological significance of the NNR is further demonstrated by its designation as a Special Area of Conservation (SAC) under the EU 92/43/EEC Habitats Directive with wetland habitats highlighted in the SAC designation including Northern Atlantic wet heaths with *Erica tetralix* L. (habitat 4010), active raised bogs (7110), degraded raised bogs (7120), and transition mires and quaking bogs (7140).

### Hydrological/Hydraulic Model

Duranel (2015) and Duranel et al. (2021) detail the Dauges MIKE SHE/MIKE 11 model so that it is briefly reviewed herein. MIKE SHE simulates the land phase of the hydrological cycle and includes approaches to represent evapotranspiration, unsaturated zone, saturated zone and overland flow processes. Although often described as a fully distributed, physically-based model, some process descriptions use simpler conceptual, semi-distributed approaches that can be selected according to data availability and modelling objectives (Graham and Butts 2005; Refsgaard et al. 2010). The MIKE 11 1-dimensional hydraulic model represents channel flow and is dynamically coupled to MIKE SHE to simulate bi-directional exchanges between channels and both the saturated zone and overland flow (Thompson et al. 2004). MIKE SHE/MIKE 11 models have been employed in studies of a range of wetlands with some applications including climate change impact assessments (e.g. Al-Khudhairy et al. 1999; Thompson 2004; Hammersmark et al. 2008; Bourgault et al. 2014; House et al. 2016a; Gardner et al. 2019; Thompson et al. 2023).

The Dauges MIKE SHE/MIKE 11 model combines physically-based and conceptual process descriptions to reduce computational demands, thereby producing acceptable run times, whilst still representing key hydrological processes (Duranel et al. 2021). A 10 m × 10 m model grid results in 23,111 cells. Elevation is specified using a digital elevation model (DEM) based on aggregating three datasets: an approximately 5 m spatial resolution, 10 cm vertical accuracy differential geo-positioning system (dGPS) survey within the mire, the nationwide IGN BD Alti 25 m spatial resolution DEM for the southern part of the catchment, and, for the remainder of the model domain, digitised points and contours from 1:1000 topographic maps produced using traditional surveys and stereo-photogrammetry (Duranel 2015). Saturated zone (SZ) flow and water levels are modelled using an iterative implicit finite difference technique to solve the 3-dimensional Darcy equation. Two SZ computational layers are based on field investigations that include geological drilling, electrical resistivity tomography and available outcrops (Duranel 2015). Fissured granite is represented by the lower layer with a fixed depth of 55 m below the ground surface and spatially uniform hydraulic properties (established via calibration). Depth and properties of the upper layer are spatially variable. In the mire this layer represents peat with its depth based on the boundary between peat and underlying mineral formations (0.5 m minimum to avoid numerical instabilities). On mineral soils, where the upper layer corresponds to a complex of soil, peri-glacial formations, saprolite and, in much of the catchment, the top of the fissured granite, depth is 2 m below the ground surface to correspond with the largest vegetation root depth (woodland) since the root zone cannot extend beyond the first SZ computation layer (see Duranel et al. (2021) for details of SZ computational layer definition). A zero flow SZ boundary is specified around most of the domain, the exception being its downstream end where the catchment flows into a lower etch-basin. Here a constant gradient boundary follows the assumption that the water table slope follows the topographic surface.

The empirical two-layer unsaturated zone (UZ) model (Yan and Smith 1994) is employed since it is particularly suitable for shallow water table conditions (e.g. Thompson 2012). It is also computationally efficient and smaller data demands compared to the full Richards equation means parameters (saturated hydraulic conductivity, volumetric soil moisture at saturation, field capacity and wilting point) could be established via calibration. Given some limitations of the two-layer UZ model in representing interception in woodland, the dominant land cover of the Dauges catchment (see Duranel 2015), evaporation from interception is modelled outside of MIKE SHE using equations employed in the HYLUC model (Calder 2003). Vegetation distribution is based on a CORINE biotope vegetation map reclassified to nine categories (needle-leaved evergreen woodlands, broad-leaved deciduous woodlands, mixed woodlands, dry heathland, clearings and shrubs, meadows and pastures, wet woodlands, wetland, impervious areas). For tall vegetation (woodland, heath and shrubs) distinctions are made between wet-time evaporation (evaporation from interception, modelled outside MIKE SHE) and dry-time evapotranspiration (transpiration and evaporation from the ground surface, simulated using the two-layer UZ model). Meteorological data (precipitation and reference Penman-Monteith evapotranspiration) are derived from the Saint-Léger-la-Montagne meteorological station. For tall vegetation, precipitation is adjusted to reflect water lost via evaporation of intercepted water, calculated following the HYLUC method with parameters calibrated to achieve bulk interception ratios matching those in the literature for similar vegetation and environmental conditions (e.g. Delgado et al. 2010). For short vegetation (grassland and mire) no distinction is made and evapotranspiration is fully modelled within MIKE SHE.

The MIKE 11 model includes the principal channels with cross-sections established using dGPS surveys. All MIKE 11 branches are coupled to MIKE SHE using the approach described by Thompson et al. (2004). Channel flow is simulated using the kinematic routing method which is computationally fast and generally accurate for fast-flowing streams with no backwater. It does impact representation of stream stage and in turn channel overtopping and inundation of adjacent MIKE SHE grid cells. This is considered acceptable since over-bank flooding is restricted to a very narrow band along the main stream and a small downstream section of the mire. Consequentially exchanges between the MIKE SHE overland component and MIKE 11 are assumed to only occur towards the latter - i.e. no overbank inundation occurs but flooding is simulated if the water table intercepts the ground surface or precipitation falls on saturated surfaces with runoff being intercepted by MIKE 11. Overland flow is represented using the 2D, finite-difference, diffusive wave approximation of the Saint-Venant equations solved using the successive over-relaxation method.

The model was calibrated and validated against observations of daily mean groundwater table depth and stream discharge (see Duranel et al. 2021). Groundwater was measured using pressure transducers within 16 shallow dip wells in the mire or in mineral soils on lower slopes. Discharge records for the mire’s outlet at Pont de Pierre and in three upper stream reaches were derived from similar water level observations and a rating curve established via spot gaugings (Pont de Pierre) or standard equations for v-notch weirs (Dingman 1994). Calibration parameters included hydraulic parameters of both SZ computational layers (horizontal and vertical saturated hydraulic conductivity, specific yield), the two-layer UZ parameters for peat and mineral soils (saturated hydraulic conductivity, volumetric soil moisture at saturation, field capacity and wilting point) and Manning’s n of the stream channels. Whilst the model was run from 01/08/1998 (installation of the Saint-Léger-la-Montagne meteorological station) to 31/12/2013 (end of intensive field monitoring), calibration and validation periods were limited by groundwater depth and stream discharge data. To account for slightly different lengths of observed data and to exclude model spin-up, the calibration period was 01/01/2011–31/12/2012 for groundwater and 01/01/2011–30/06/2012 for discharge whilst the validation period was 01/01/2013–31/12/2013 and 01/07/2012–31/12/2013 for groundwater table depth and discharge, respectively.

**Fig. 2:**
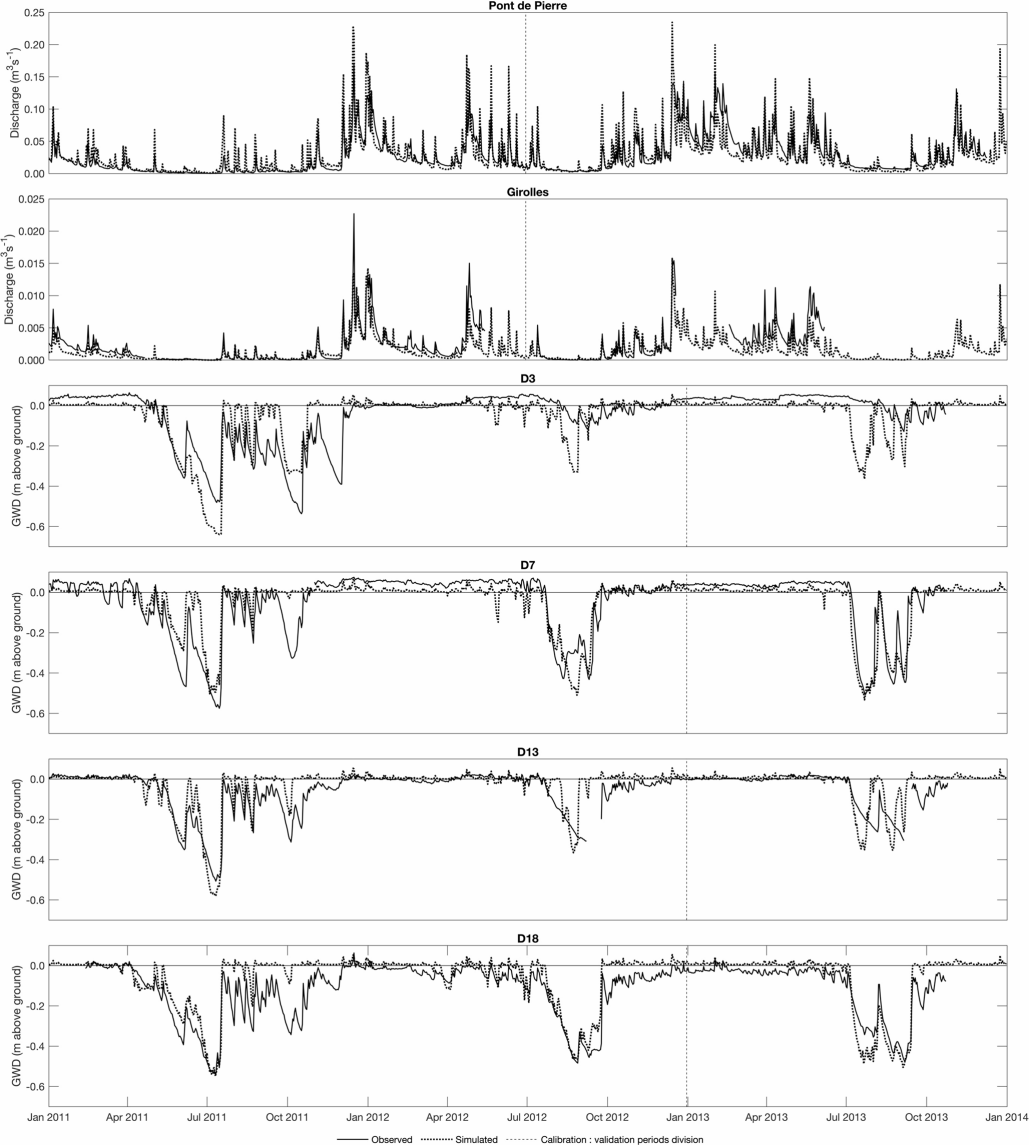
Observed and simulated stream discharge at two gauging stations and groundwater level (GWD) at the location of four dip wells within the Dauges mire for the period 01/01/2011–31/12/2013. The periods used for calibration and validation are indicated. Note different y-axis scales for the two gauging stations

According to the classification of Moriasi et al. (2007), model performance for discharge is very good (Nash-Sutcliffe Efficiency; NSE > 0.75) to satisfactory (NSE > 0.5). Figure 2 shows that the model simulates seasonal variations in discharge characterised by high flows in autumn and especially winter and a gradual decline in spring to summer lows. Individual precipitation events superimpose flashy discharge responses on this seasonal cycle. Model performance for groundwater table depth is variable, at least in part, due to more observation locations. This is illustrated in Fig. 2 which also shows observed and simulated water table depth at four representative dip wells. At most locations performance, again assessed using NSE, is very good to satisfactory. Prolonged periods in autumn-spring of high water levels, largely coincident with the ground surface, are simulated as are summer drawdowns which vary in magnitude and duration between years and locations.

### Simulation of Climate Change

Climate change scenarios were developed using the DRIAS-2020 dataset which is based on high-resolution regional climate simulations from the Euro-Cordex ensemble (Météo-France 2020). Projections are available for three widely used Representative Concentration Pathways (RCPs; Moss et al. 2010). These RCPs (RCP2.6, RCP4.5 and RCP8.5) are associated with targets for radiative forcing of between 2.6 and 8.5 Wm^− 2^ by 2100 and encompass several assumptions concerning global population, economic development and greenhouse gas emissions. The high spatial resolution (8 km) DRIAS-2020 projections, covering the whole of France, are based on simulations from 12 Global Climate Model (GCM)/Regional Climate Model (RCM) pairs, followed by statistical correction of climate model results using observed climatology. Although results are available for all 12 GCM/RCM pairs for the historical period (1951–2005) and 2006–2100 for RCP8.5, they are only available for eight and ten pairs for RCP2.6 and RCP4.5, respectively. As a result, projections are available for 30 future climate simulations (Table 1).

**Table 1:** DRIAS-2020 Global Climate Model (GCM)/Regional Climate Model (RCM) pairs used to develop climate change scenarios for the three Representative Concentration Pathways (RCPs)

No.	GCM	RCM	RCP2.6	RCP4.5	RCP8.5
1^+‡^	CNRM-CM5	Aladin63 V2	•	•	•
2^+‡^	CNRM-CM5	Racmo22E v2	•	•	•
3^‡^	IPSL-CM5A-MR	WRF381P		•	•
4^‡^	IPSL-CM5A-MR	RCA4		•	•
5	HadGEM2-ES	RegCM4-6	•		•
6^‡^	HadGEM2-ES	CCLM4-8–17		•	•
7^+‡^	EC-EARTH	Racmo22E v2	•	•	•
8^+‡^	EC-EARTH	RCA4	•	•	•
9^+‡^	MPI-ESM-LR	CCLM4-8–17	•	•	•
10^+‡^	MPI-ESM-LR	REMO 2009	•	•	•
11^‡^	NorESM1-M	HIRHAM5 v3		•	•
12	NorESM1-M	REMO 2015	•		•

*+ GCM-RCM pairs used to calculate EM*_*6*_*for all three RCPs; ‡ GCM-RCM pairs used to calculate EM*_*10*_* for RCP4.5 and RCP8.5 (Note that in the case of RCP4.5*,* EM*_*10*_*= EM*_*all*_*)*

DRIAS-2020 provides a series of meteorological indicators at monthly and seasonal resolutions for three 30-year time slices over the 21 st Century compared to a 30-year (1976–2005) reference (or baseline) period. The current study focusses on the 2041–2070 (referred to as 2050 s and representative of the middle of the current century) and 2071–2100 (2080s, end of century) time slices. Changes (delta factors) in monthly mean precipitation (% change), mean monthly minimum and maximum temperatures (ºC change), and monthly mean wind speed (ms^− 1^ change) were acquired for the DRIAS-2020 grid square covering the Dauges catchment for each of the 30 climate simulations and both time slices. The resulting 60 scenarios were extended by calculating ensemble means of each monthly delta factor for a given time slice and RCP. The different number of GCM/RCM pairs for the three RCPs complicates direct comparison between different levels of radiative forcing. Whilst one ensemble mean (EM_all_) employed all sets of delta factors available for a given RCP (i.e. eight, ten and 12 for RCP2.6, RCP4.5 and RCP8.5, respectively), another two imposed some consistency. EM_6_ employed the six GCM/RCM pairs available for all three RCPs enabling some comparisons between magnitude of radiative forcing. A second ensemble mean, EM_10_, used the ten pairs providing data for the two higher radiative forcing scenarios (equivalent to EM_all_ for RCP4.5). By definition, EM_10_ could not be calculated for RCP2.6 scenarios. With the addition of the three ensemble means, this approach provides 38 scenarios for both the 2050 s and 2080 s (i.e. 76 in total).

Following studies using similar climate projections, most notably the UK Climate Projections (e.g. Acreman et al. 2009, 2012; Thompson 2012; Thompson et al. 2009, 2017b, 2023), scenario precipitation was derived by multiplying original precipitation time series by projected monthly percentage changes. The original minimum and maximum temperature, as well as wind speed, time series were perturbed by addition of their respective monthly delta factors before recalculating reference Penman-Monteith evapotranspiration and HYLUC-based evaporation from interception. Given that DRIAS-2020 delta factors are not available for relative humidity and global radiation, the original time series were employed. This does deviate from studies employing the UK Climate Projections (e.g. Thompson et al. 2017b, 2023) which provides delta factors for humidity and downward surface shortwave radiation. As such there is some uncertainty in scenario evaporation/evapotranspiration given, for example, the reductive effect of elevated humidity (and vice versa - e.g. Lemaitre-Basset et al. 2022). In common with earlier studies the method retains original climate variability but excludes changes in factors such as rainfall intensity or distribution within a month (number of wet days; Chiew et al. 1995; Fowler et al. 2007; Graham et al. 2007). It does, however, enable robust comparisons of average outcomes of projections and has been widely used in climate change impact studies (Arnell and Reynard 1996; Arnell 2004; Thompson et al. 2016; Ho et al. 2016; Chan et al. 2020).

The MIKE SHE/MIKE 11 model was forced with each set of perturbed meteorological data with the simulation period defined as 01/08/1998–31/12/2013, replicating the original model hereafter referred to as the baseline. Baseline-scenario comparisons used the period 01/01/2001–31/12/2013 to exclude model spin-up. The impacts of climate change upon stream flow were assessed via comparisons of baseline and scenario discharge upstream and downstream of the mire. Similar comparisons were undertaken for groundwater depths for MIKE SHE grid cells corresponding to locations of representative dip wells as well as across the whole mire.

### Translation of Hydrological Changes to Impacts on the Mire Vegetation Distribution

Implications of climate-change upon mire vegetation were investigated using previously established relationships between peat-based ecosystems and spatially distributed hydrological variables from the baseline MIKE SHE/MIKE 11 model. Duranel et al. (2021) identified particularly strong agreement between the extent of mire vegetation (Fig. 1) and simulated groundwater seepage (upwelling from the water table to the surface) in September, the month when seepage reaches its minimum. It was argued that the boundaries of peat-based ecosystems are strongly determined by the presence of groundwater seepage in the driest month as it will sustain the wet conditions required for mire vegetation whilst limiting peat loss (Duranel 2015). Optimisation of a Cohen’s kappa agreement function (Congalton 1991) determined that the threshold mean September groundwater seepage best discriminating between mire and non-mire vegetation was 0.005 mm d^− 1^. The value of kappa (0.845) established for this threshold was slightly higher than one obtained using mean annual groundwater depth which established 0.166 m below ground as the threshold (kappa: 0.841). The September seepage threshold was therefore used to translate simulated hydrological changes to ecological responses (the analysis using mean groundwater depth is in Supplementary Materials SM6). The MIKE SHE grid cells in which mean September seepage (mean annual groundwater depth) during the period 01/01/2001–31/12/2013 exceeded the threshold were identified for the baseline and each scenario. Baseline-scenario comparisons were based on changes in the area in which the threshold was exceeded and changes in simulated seepage within these areas.

## Results

### Climate Change Impacts on Meteorological Forcing

There is inter-scenario variability in magnitude and direction of change in annual precipitation although most GCM/RCM pairs project increases (45 of 60; Table 2; Supplementary Materials SM1). Whilst for RCP2.6 in the 2050 s (2080s) all eight (seven) pairs project increases, results are not available for three pairs (4, 6 and 11) that tend to project declines for RCP4.5 and RCP8.5. For RCP8.5 in the 2080 s, an additional three pairs (i.e. half of the 12) project declines. Across all pairs available for an individual RCP/time slice combination inter-pair ranges of change in annual precipitation tend to increase with magnitude of radiative forcing and more distant time slice. In the 2050 s the range for RCP2.6 is equivalent to 9.5 percentage points compared to 21.6 percentage points for RCP8.5 (Table 2). Corresponding ranges for the 2080 s are 16.3 and 22.5 percentage points although the smaller range for RCP2.6 may be influenced by absence of data for pairs projecting declines for higher radiative forcing. The same caveat applies to the ensemble means but differences between RCP/time slice combinations are relatively small and all project modest increases in annual precipitation. EM_6_ in the 2050s (2080s) projects increases of 5.4%, 4.0% and 3.3% (5.5%, 4.4% and 1.3%) for RCP2.6, RCP4.5 and RCP8.5, respectively. Increases for EM_10_ are marginally smaller and those for EM_all_ slightly larger.

**Table 2:**
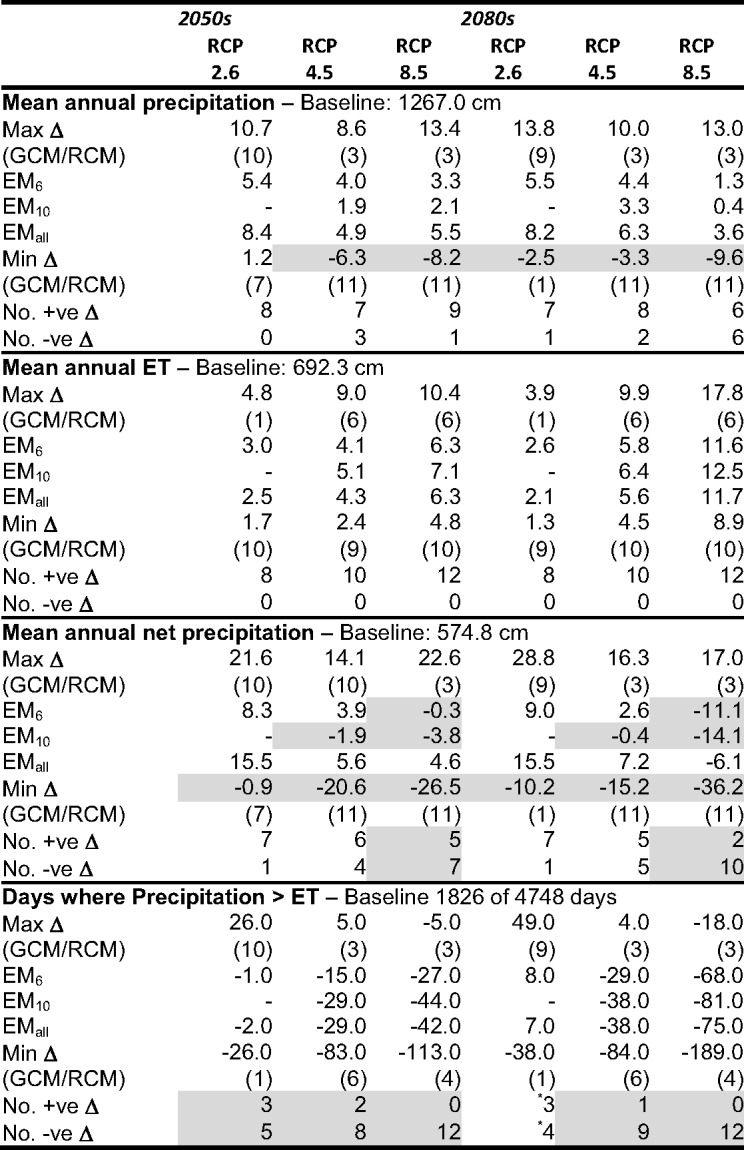
Baseline mean annual precipitation, annual reference evapotranspiration (ET), annual net precipitation (mm) and number of days when precipitation > ET; maximum, ensemble mean (EM) and minimum changes in these climate variables (% and days) across GCM/RCM pairs for each climate change scenario (pairs responsible for the extreme changes); and frequency of positive and negative changes in these variables. Baseline data are for the period 01/01/2001–31/12/2013. Shaded cells indicate reductions compared to the baseline and where the majority of pairs project declines in the value of the variables

**Fig. 3:**
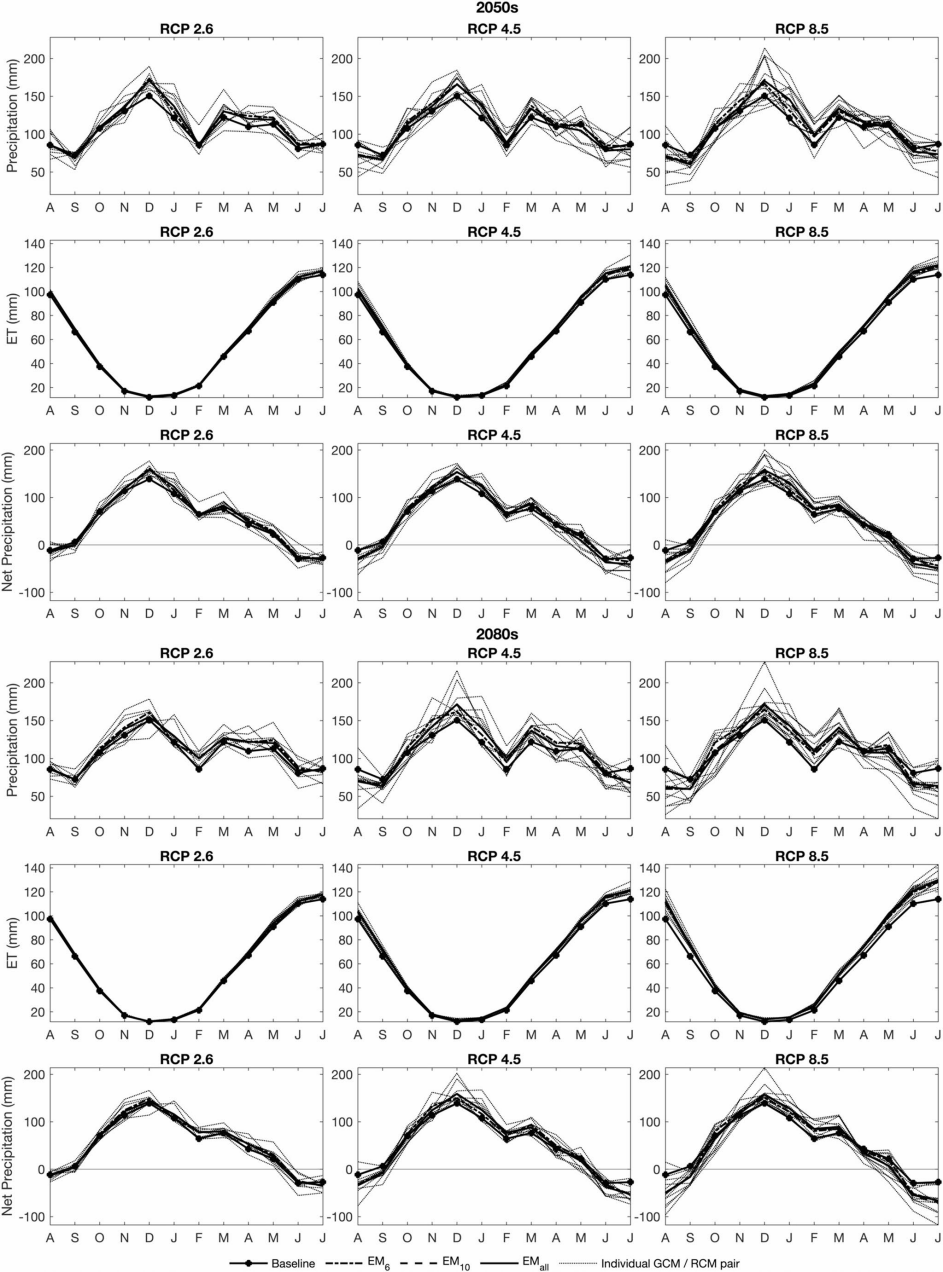
Mean monthly precipitation, reference evapotranspiration (ET) and net precipitation for the baseline and each climate change scenario in the 2050 s (top half) and 2080 s (bottom half)

Variability in mean monthly precipitation across GCM/RCM pairs is considerable and tends to increase with radiative forcing and into the future (Fig. 3) although the different number of pairs between RCPs should be acknowledged. Total winter (November–February) precipitation (baseline 488.7 mm) increases for all pairs for all RCPs in both time slices with just two exceptions. The largest increases for a given RCP/time slice are consistently in double figures (overall maximum: 39.4%). For EM_6_ increases are in the range 6.6–10.4% in the 2050 s and 7.3–12.0% in the 2080 s (RCP2.6 and RCP8.5 projecting the limits of these ranges). The corresponding values for the other ensemble means are characteristically slightly (3–6 percentage points) larger. Declines in summer precipitation (June–September, baseline 325.7 mm) dominate although this trend is less equivocal and for RCP2.6 five out of eight pairs project increases for both time slices. The ensemble means project small (< 2.5%) increases. In contrast, eight (nine) of ten pairs project declining summer precipitation for RCP4.5 in the 2050 s (2080s) increasing to ten of 12 for RCP8.5 (both time slices). In percentage terms, declines are more variable than for winter increases (1.3–31.2% and 1.3–39.9% for RCP4.5 and RCP8.5, respectively in the 2050 s; 2.2–34.6% and 2.4–57.1% for the 2080 s with the few increases being less then 12%). EM_6_ projects declines of 5.6% (12.4%) and 9.2% (23.4%) for RCP4.5 and RCP8.5, respectively in the 2050 s (2080s). Declines for the other ensemble means are slightly (1–4%) larger.

All GCM/RCM pairs project increased mean annual evapotranspiration for all three RCPs in both time slices (Table 2; Supplementary Materials SM1). In the 2050 s increases from the baseline (692.3 mm) are in the range 1.7-4.8% (RCP2.6) to 4.8–10.4% (RCP8.5) and in the 2080 s 1.3–3.9% to 8.9–17.8%. For any given pair and time slice, gains in annual evapotranspiration grow with radiative forcing. Similarly, increases for a given pair for the two higher radiative forcing scenarios consistently grow from the 2050 s to the 2080s. Changes for the ensemble means follow these trends with increases for EM_6_ in the 2050 s (2080s) equalling 3.0%, 4.1% and 6.3% (2.6%, 5.8%, 11.6%) for the progressively higher radiative forcing. Increases for EM_10_ and EM_all_ are very similar.

The seasonal distribution of reference evapotranspiration is unchanged for all scenarios and inter-GCM/RCM pair variability is relatively small (Fig. 3). Increased mean monthly evapotranspiration dominates although some (mostly one but up to three) pairs project declines in a few winter months for RCP2.6 and RCP4.5. Mean evapotranspiration increases in every month for all scenarios under RCP8.5. The largest absolute increases are concentrated in summer (July and August in particular) when baseline monthly evapotranspiration is high. The magnitude of changes increases with radiative forcing and more distant time slice and is reflected in the ensemble means; EM_6_ projects a mean July–August increase for RCP2.6 in the 2050 s (2080s) of 2.9% (2.8%) compared to 6.5% (13.0%) for RCP8.5. Increases for the other ensemble means are slightly (< 1.5%) larger.

The number of GCM/RCM pairs projecting declines in annual net precipitation (precipitation – reference evapotranspiration) for a given RCP/time sluice is characteristically larger than for annual precipitation (Table 2; Supplementary Materials SM1). Just one pair projects declines for RCP2.6 in both time slices. For RCP4.5 four and five of ten pairs project declines in the 2050 s and 2080 s, respectively, whilst for RCP8.5 seven (2050s) and ten (2080s) of 12 pairs project such declines. As for annual precipitation, inter-pair ranges of change in net precipitation grows with radiative forcing and into the future with ranges being larger, when expressed in percentage points, than for precipitation. For the two lower radiative forcing scenarios EM_6_ projects increases, albeit of declining magnitude from RCP2.6 to RCP4.5 (8.3% and 3.9% for the 2050 s; 9.0%, 2.6% for the 2080 s) whilst declines are projected for RCP8.5 (reductions of 0.3% and 11.1% for the 2050 s and 2080 s, respectively). Results for the other ensemble means follow these trends.

Inter-scenario differences in projected mean monthly net precipitation exhibits similar trends to those for precipitation (Fig. 3). This includes wetter winters although this is slightly less equivocal than for precipitation (a single GCM/RCM pair projects small declines for RCP2.6 in the 2080 s and RCP8.5 in both time slices). Increases in November–February net precipitation (baseline: 425.3 mm) for EM_6_ are in the range 7.0–10.7% in the 2050 s and 7.9–11.5% in the 2080 s (increases for EM_10_ and EM_all_ slightly larger). Summer net precipitation (June–September, baseline − 62.1 mm) declines for most scenarios with this drying trend being more pronounced than for precipitation. Declines for EM_6_ under RCP2.6 range between 8.0% (2050s) and 12.8% (2080s), gaining in magnitude for RCP4.5 (54.0% and 99.5%) and RCP8.5 (85.8% and 196.8%). The declines for other ensemble means are larger (by up to c.10 percentage points), especially for higher radiative forcing.

On average under baseline conditions evapotranspiration exceeds precipitation in three months (June–August; Fig. 3). There is a tendency for the frequency of negative net precipitation to increase with magnitude of radiative forcing and into the future. For example, under RCP2.6, one (2050s) and three (2080s) pairs project an additional month (September) of negative net precipitation whilst for RCP8.5 in the 2050 s, nine pairs project an additional month of negative mean net precipitation with one projecting an increase of two months. The corresponding figures for the 2080 s are seven and four pairs. Whilst no change in incidence of negative mean monthly net precipitation is projected by the ensemble means for RCP2.6, under RCP4.5 and RCP8.5 (both time slices), a single additional month (September) experiences a rainfall deficit. Similar trends are evident when the analysis is based on days during the simulation period (Table 2). The number of pairs projecting a higher incidence of negative daily net precipitation and the number of days subject to this change for individual pairs and ensemble means grow with magnitude of radiative forcing.

### Climate Change Impacts on Stream Discharge

Climate change impacts on mean stream discharge as well as Q5 and Q95 discharges (discharges exceeded for 5% and 95% of the time and representative of high and low flows, respectively) downstream of the mire (Pont de Pierre) and at Rocher, on the stream flowing into the mire from the south with changes similar to those at other upstream stations, are summarised in Table 3 (results for all scenarios are in Supplementary Materials SM2).

**Table 3:**
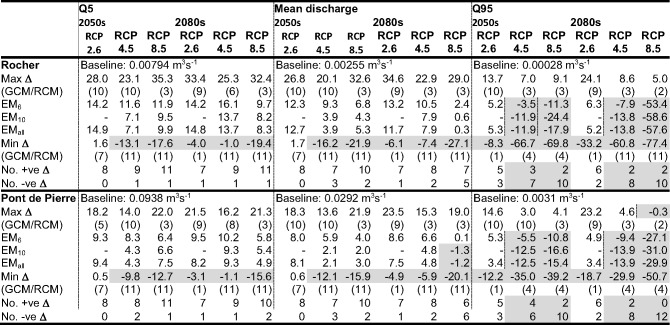
Baseline simulated Q5, mean discharge and Q95 (m^3^s^− 1^) for two gauging stations in the Dauges catchment (Rocher upstream of the mire, Pont de Pierre downstream of the mire – see Fig. 1); maximum, ensemble mean (EM) and minimum changes in these discharge metrics (%) across GCM/RCM pairs for each climate change scenario (pairs responsible for the extreme changes); and frequency of positive and negative changes in metrics. Shaded cells indicate reductions in metrics compared to the baseline and where the majority of pairs project declines in the value of the metrics

Gains in mean discharge above the mire (Rocher) dominate. Of the 60 RCP, time slice, GCM/RCM pair combinations, 47 (78.3%) project increases. There is some consistency in the pairs producing the largest increases (pairs 9 or 10 for RCP2.6, pairs 3 or 10 for RCP4.5 and RCP8.5). When declines are projected, they are more associated with higher radiative forcing and more distant time slice with pair 11 producing most of the largest declines. Inter-GCM/RCM pair range of changes tends to grow with radiative forcing especially in the earlier time slice. All ensemble means project increased mean discharge for all three RCPs with magnitude of increases reducing slightly with radiative forcing; increases for EM_6_ range from 12.3% (13.2%) for RCP2.6 to 6.8% (2.4%) for RCP8.5 in the 2050 s (2080s). Increases for the other ensemble means tend to be a few percentage points smaller. Trends downstream of the mire (Pont de Pierre) are very similar to those upstream with 46 (76.7% of the 60) pairs projecting increases although for RCP8.5 (2080s) half of the pairs project declines. The same pairs account for the largest increases and decreases as for Rocher. There is a tendency for increases and decreases at the downstream station to be smaller than upstream (mean difference across all increases, the dominant direction of change: 4.4 percentage points). Inter-pair ranges of change in mean discharge also increase with radiative forcing but for a given RCP/time slice combination they are smaller (by between 7 and 17 percentage points) than those upstream. This is repeated for the ensemble means; declines dominate but they are smaller than those at Rocher.

Inter GCM/RCM pair variability in direction of change in high flows (Q5) is much less equivocal than for mean discharge. At Rocher and Pont de Pierre 55 (91.7%) and 53 (88.3%), respectively of the 60 combinations project increases (Table 3). The pattern of increases for a given RCP/time slice combination, including pairs responsible for the extremes of change, are broadly similar at the two stations. Inter-pair ranges of change follow the same trends as those for mean discharge (and are usually within a few percentage points of these ranges). Percentage changes in Q5 of either direction tend to be larger above the mire compared to below it (56 of the 60). Mean differences across different pairs for individual RCP/time slice combinations range between 3.9 (RCP4.5, 2050 s) and 6.0 (RCP4.6, 2080 s) percentage points. All ensemble means project increasing Q5 at both stations. Inter-scenario variations are relatively small; overall ranges of increases for EM_6_ at Rocher and Pont de Pierre across all time slice/pair combinations are 9.7–16.1% and 5.8–10.2%, respectively. Increases for the other ensemble means are very slightly smaller.

Declines in low flows dominate with 40 (66.7%) and 41 (68.3%) of the 60 combinations projecting declines in Q95 at Rocher and Pont de Pierre, respectively (Table 3). The number of pairs projecting declines increases with radiative forcing from two or three (out of eight) for RCP2.6 to ten, and in the case of Pont de Pierre for the 2080 s, all 12 for RCP8.5. This is accompanied by a general increase in the magnitude of declines (and smaller increases when they are projected). The upstream vs. downstream differences in changes in Q95 are notably larger than those for mean and Q5 discharge with percentage declines at Pont de Pierre being considerably smaller than those at Rocher, at least for RCP4.5 and RCP8.5. As a result, the overall inter-pair ranges of change in Q95 are, with the exception of RCP2.6 in the 2050 s, larger above the mire than below it. The ensemble means project relatively small increases (3.4–6.3%) in Q95 at Rocher and Pont de Pierre for RCP2.6 in both time slices. With higher radiative forcing, progressively larger declines in Q95 are projected. In most cases declines at Rocher and Pont de Pierre are similar although they are notably larger at the first station for. RCP8.5 in the 2080 s (e.g. decline of 53.4% compared to 27.1% for EM_6_). EM_10_ and EM_all_ project larger declines compared to EM_6_ (by on average 7.2 and 5.0 percentage points, respectively).

The range of flows, indexed by the difference between Q5 and Q95, increases in most cases. Above the mire (Rocher), just four (6.7%) of the 60 combinations exhibit a smaller range compared to the baseline whilst below the mire (Pont de Pierre) the equivalent figure is seven (11.7%). Ensemble means all project increases in these range which decline very slightly with radiative forcing. For example, at Rocher EM_6_ projects increases of 14.6% (14.4%) for RCP2.6 in the 2050 s (2080s) and 12.8% (12.0%) for RCP8.5. These ranges are slightly smaller for Pont de Pierre.

**Fig. 4:**
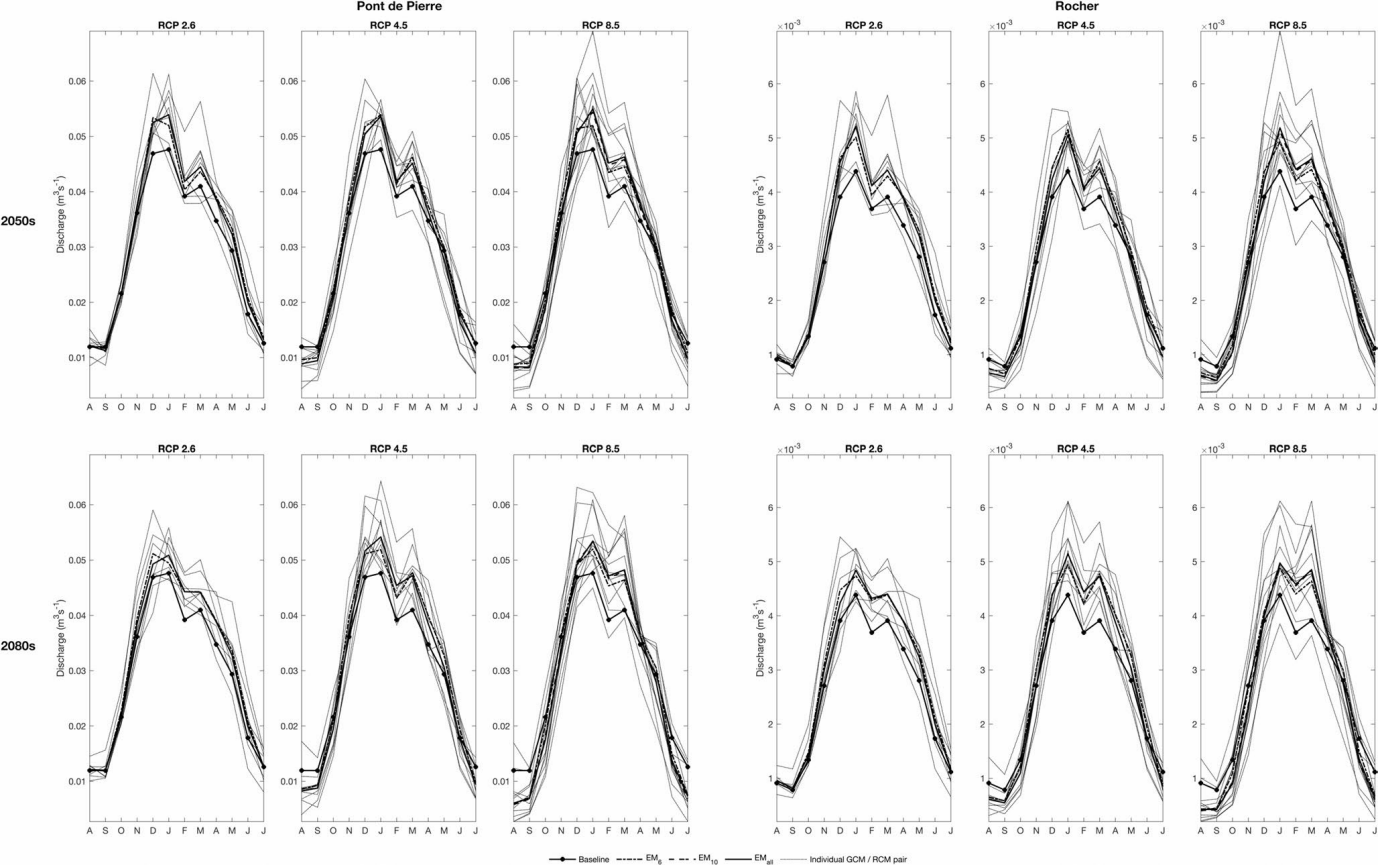
Mean monthly discharge at Rocher and Pont de Pierre (above and below the main mire, respectively) for the baseline and each climate change scenario in the 2050 s (top half) and 2080 s (bottom half). Note different y-axis scales for the two gauging stations

Figure 4, which shows the river regimes (mean monthly discharges) at Rocher and Pont de Pierre for the baseline and each scenario demonstrates the dominant trends for higher winter flows, declines in summer and an increase in seasonal ranges. In the 2050 s, mean winter (November–February) discharge at Rocher increases for eight (i.e. all), eight and 11 GCM/RCM pairs for RCP2.6, RCP4.5 and RCP8.5, respectively. The equivalent figures for the 2080 s are seven, nine and nine pairs. Mean winter discharge at Pont de Pierre increases for seven (RCP2.6), eight (RCP4.5) and 11 (RCP8.5) pairs in the 2050 s and seven (RCP2.6) and ten (RCP4.5 and RCP8.5) pairs in the 2080s. Ensemble means project declines in winter flows for all scenarios with relatively small inter-scenario variations (e.g. ranges of change for EM_6_ are 9.0–12.9% for Rocher and 7.7–9.5% for Pont de Pierre). The number of pairs projecting reduced mean summer (June–September) discharge at Rocher for the 2050 s rises from three, through six to eight with elevated radiative forcing. The equivalent figures for Pont de Pierre are four, eight, and ten. In the 2080 s, two, six, and ten pairs (Rocher) and three, six and ten pairs (Pont de Pierre) project declines for RCP2.6, RCP4.5 and RCP8.5, respectively. Whilst the ensemble means project increasing mean summer discharge for RCP2.6 at Rocher (EM_6_: 12.5% and 11.2% for the 2050 s and 2080 s, respectively) and Pont de Pierre (5.3 and 4.9%), declines, increasing with magnitude of radiative forcing, are projected for RCP4.5 and RCP8.5. For example, EM_6_ projects declines at Rocher of 4.3% (10.5%) for RCP4.5 and 10.0% (33.0%) for RCP8.5 in the 2050 s (2080s). Those for Pont de Pierre are slightly larger (by on average 2.7 percentage points).

### Climate Change Impacts on Mire Groundwater Levels

**Fig. 5:**
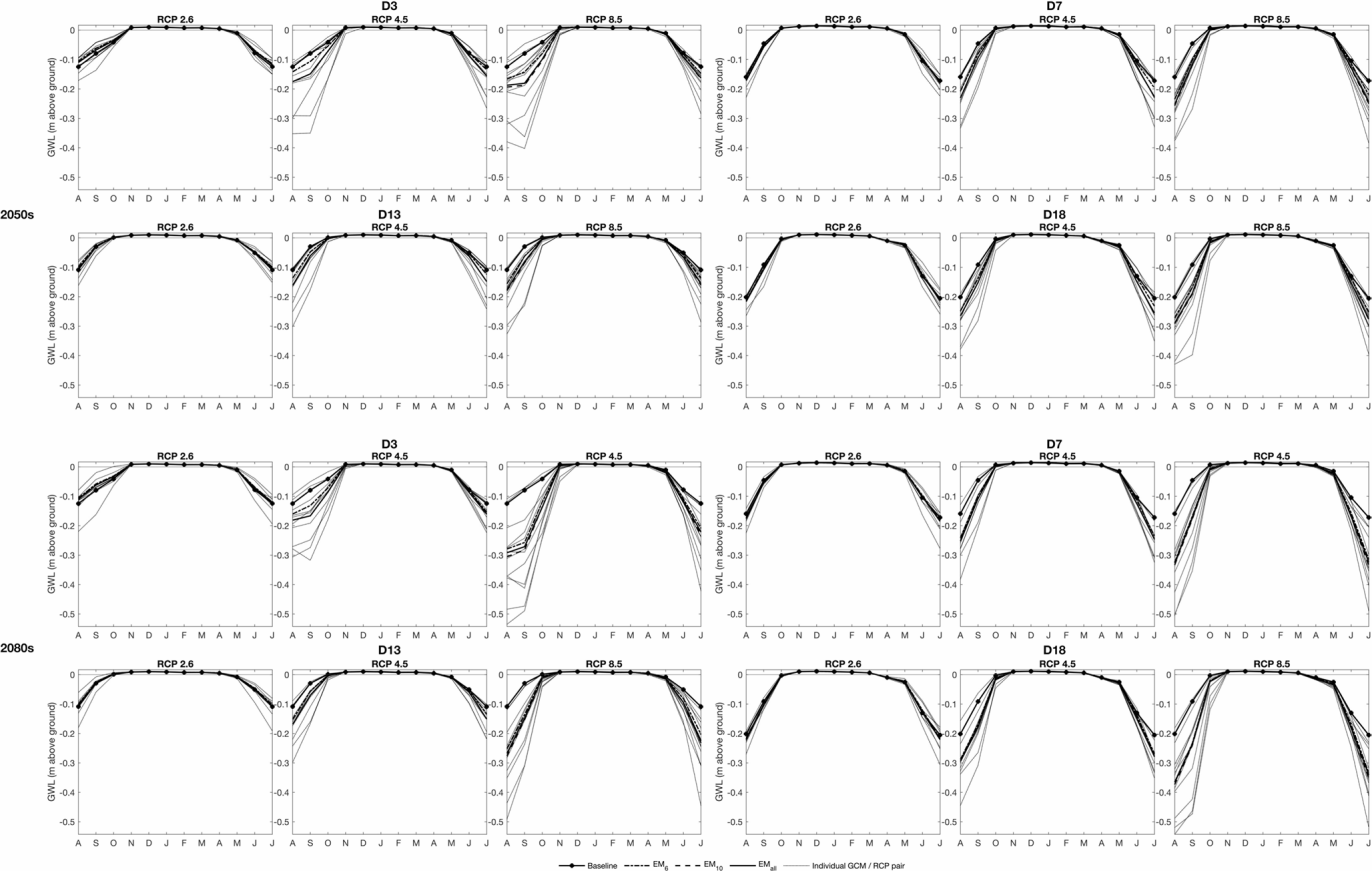
Mean monthly simulated groundwater depth (GWD) at the locations of four dip wells within the Dauges peatland for each GCM/RCM pair and the three ensemble means (EM) for the three RCP scenarios in the 2050 s and 2080s

The climate change impacts on mean groundwater depth (GWD), GWD-5 and GWD-95 (water table depths exceeded for 5% and 95% of the time, indicative of high and low groundwater conditions, respectively) at the locations of the four representative dip wells used to demonstrate model performance are summarised in Table 4 (Supplementary Materials Table SM3.1 provides the complete results for these wells for all scenarios). Three wells are along an approximately southwest-northeast transect beginning close to the southern edge of the mire (D3), crossing the stream running through the mire (D7 is south of, but close to, this channel) and extending into a large area of peat to the north of Puy Rond (D13). D18 is upstream of the transect and close to the stream. Figure 5 provides baseline and scenario mean monthly groundwater depths for the four wells.

**Table 4:**
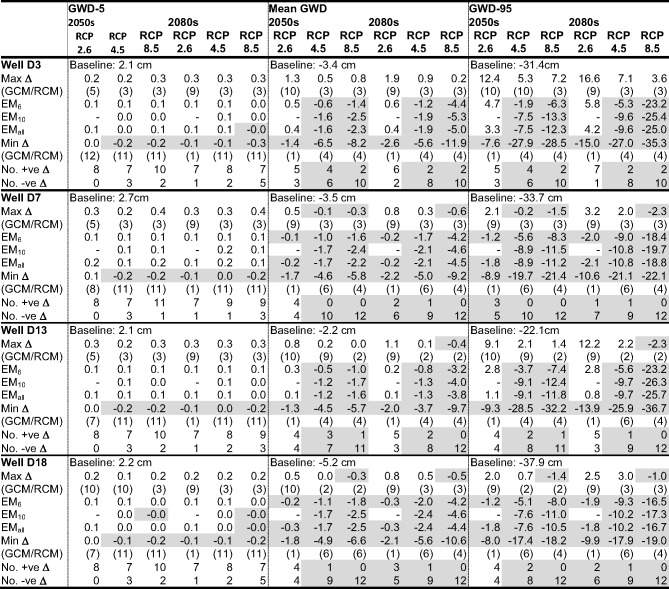
Baseline simulated GWD-5, mean groundwater depth (GWD) and GWD-95 (cm above ground) at the locations of four dip wells within the mire; maximum, ensemble mean (EM) and minimum changes in these groundwater metrics (cm) across GCM/RCM pairs for each climate change scenario (pairs responsible for the extreme changes); and frequency of positive and negative changes in metrics. Shaded cells indicate reductions in metrics compared to the baseline and where the majority of pairs project declines in the value of the metrics. See Fig. 1 for the locations of dip wells

Climate change driven impacts on winter groundwater levels are relatively small. Baseline GWD-5 at the four wells is consistently a little over 2 cm (Table 4). Across the 240 combinations of RCPs, time slices, GCM/RCM pairs and dip wells increases in GWD-5 are projected in 194 cases (80.8%) although the incidence of increases tends to decline slightly with magnitude of radiative forcing and more distant time slice; at the four wells all eight (seven) RCP2.6 pairs in the 2050 s (2080s) project increases whilst on average increases are projected by ten and eight pairs for RCP8.5 in the 2050 s and 2080 s, respectively. However, the absolute magnitudes of changes in GWD-5 are extremely small ranging across the 240 combinations between − 0.2 cm and 0.4 cm. As a result, in most cases the ensemble means project similarly small increases in GWD-5. Under baseline conditions, the water table at the four wells is, on average, slightly (< 2 cm) above the ground surface for five (D18), six (D3) or seven (D7 and D13) months (at least November–March, extending into April or October for some; Fig. 5). Across the 240 combinations increases in these numbers occur just three times and are limited to a single month (October). Declines are more common (65 out of 240) but are again limited to a single month (normally the start of the period of highest levels) and water tables are still very close to the surface. Responses differ between wells and more declines occur with larger radiative forcing. For example, whilst at D18 just two scenarios (one each under RCP2.6 and RCP4.5 in the 2080 s) project a decline in number of months when mean monthly water table is above the ground surface, at D13 declines are more frequent (one pair for RCP2.6 in both time slices, five and eight pairs for RCP4.5 in the 2050 s and 2080 s, respectively, and ten pairs for RCP8.5 in both time slices).

Declines in the low summer water tables dominate projections (Fig. 5). On average the lowest monthly mean water tables under baseline conditions are simulated in August (Well 3) or July (the remaining three wells). Summer drawdowns tend to be larger closer to surface water drainage (D7 and especially D18, lowest monthly mean water table depths of 17.2 cm and 20.5 cm, respectively) compared to further from these features (D3: 12.5 cm and D13: 10.9 cm). Across the 240 combinations, declines in the lowest mean monthly water table depths are projected in 187 cases (77.9%). Instances of where most pairs project increases are restricted to RCP2.6 for D3 (both time slices) and D13 (2050s). In all other cases, declining summer mean monthly water tables are in the majority and their frequency increases with radiative forcing as does the inter-pair range of change for an individual scenario. At D3 (smallest number of declines), three (two) of the eight RCP2.6 pairs project declines in August mean water levels in the 2050 s (2080s). This increases to eight of the ten RCP4.5 pairs and then 10 of the 12 pairs for RCP8.5 (same frequencies in both time slices). At the other extreme, for D7 (largest number of declines) five pairs project declines in July for RCP2.6 in both time slices, eight (2050s) and nine (2080s) for RCP4.5 and all 12 (both time slices) for RCP8.5. The ensemble means project very small changes in the lowest monthly mean groundwater levels for RCP2.6 with some increases (< 2 cm). Ensemble mean levels then progressively decline for higher radiative forcing. Inter-well differences are relatively small (< 5 cm) although it is notable that the largest declines are mostly projected for D18 which, as noted, experiences the largest baseline summer drawdowns. The average decline for EM_6_ across the four wells for RCP4.5 is 2.7 cm and 6.4 cm for the 2050 s and 2080 s, respectively whilst for RCP8.5 the equivalent figures are 4.1 cm and 13.0 cm.

Projected changes in GWD-95 follow the same trends as those for the lowest monthly means (Table 4). Given the very small changes in GWD-5, the trends for GWD-95 are also indicative of changes in the range (central 90%) of water tables depths. Of the 240 combinations, GWD-95 declines in 191 cases (79.6%). The frequency of declines, their magnitude and inter-pair range of change tend to increase with radiative forcing and future time slice. For example, the number of pairs projecting declines in GWD-95 at an individual well for the 2050 s ranges between 3 and 5 (RCP2.6), 6 and 10 (RCP4.5), and 10 and 12 (RCP8.5). The equivalent figures for the 2080 s are 1–7, 8–9, and 10–12. The pairs responsible for the extremes of the ranges of change in GWD-95 shown in Table 4 are characteristically those with the largest and smallest (largest increases for some) declines in net precipitation (e.g. pairs 4 or 6 and 2 or 3). The ensemble means for RCP2.6 project small (< 6 cm) increases in GWD-95 for both time slices at D3 and D13 and even smaller increases (< 2 cm) at the other two wells. In contrast, for RCP4.5 declines are projected by all the ensemble means at each of the four wells. For EM_6_ these range between 2.0 cm and 5.6 cm in the 2050 s and between 6.3 cm and 9.3 cm in the 2080s. Declines for EM_10_ and EM1_all_ are slightly (1–6 cm) larger. This is repeated for RCP8.5 with declines projected by EM_6_ across the four wells in the range 6.3–8.3 cm (2050s) and 16.5–23.3 cm (2080s).

As a result of the very small changes in winter high water table levels and the dominance of lower summer water tables, there is an overwhelming trend for mean GWD to decline. Of the 240 combinations, mean GWD is lower than the baseline in 188 (78.3%) cases (Table 4). The reported broad trends for GWD-95 are repeated for mean GWD; the number of pairs projecting declines increases with radiative forcing and into the future as do the magnitude of the declines (with increases reducing in size) and the inter-pair ranges of change. In absolute terms, changes projected for mean GWD as well as the inter-pair ranges of a given RCP/time slice combination are smaller than those for GWD-95. For RCP2.6 in both time slices EM_6_ and EM_all_ project increases for D3 and D13 and declines at the other two wells but these changes are very small (overall range: −0.3–0.6 cm). All ensemble means project reductions in mean GWD for RCP4.6 and RCP8.5 in both time slices. On average across the four wells EM_6_ project declines of just 0.8 cm and 1.5 cm for RCP4.5 in the 2050 s and 2080 s, respectively increasing to 1.5 cm and 4.0 cm for RCP8.5. Declines for the other two ensemble means are very slightly larger (by on average just 0.6 cm).

Confirmation that results for the four wells are representative of those across the mire is provided in Table 5. This provides the same summary figures based on average values for MIKE SHE grid cells (*n* = 4332) falling within the area currently defined as mire (Fig. 1; results for all scenarios are provided in Supplementary Materials Table SM3.2). Of the 60 combinations of RCPs, time slices, and GCM/RCM pairs, 53 (88.3%) project increased mean GWD-5 within the mire, slightly higher in percentage terms than the 80.8% of 240 combinations for the four wells. Similarly, average values of mean GWD and GWD-95 across the mire decline in 71.7% and 78.3% (43 and 47 of 60) of cases, respectively, close to those for the four wells (78.3% and 79.6%). Complementarity between mire-wide mean changes and those for individual wells is repeated when comparing projections for different RCPs and time slices. For example, the number of pairs projecting lower mean GWD-95 across the mire increases with radiative forcing and, in general, slightly more pairs project declines in the 2080 s than in the 2050s. Half of the eight RCP2.6 pairs project declines in GWD-95 (both time slices). This increases through eight (nine) of ten RCP4.5 pairs, to ten (12) of the 12 RCP8.5 pairs in the 2050 s (2080s). In common with the results for individual wells, the size of declines in both mean GWD and GWD-95 tend to increase (size of increases tend to decline) with magnitude of radiative forcing and more distant time slice driving similar trends in changes for the ensemble means (Table 5). The size of these changes for individual scenarios are very similar to those reported for the four wells with some of the same pairs accounting for the extremes in projected changes. The range of changes in mean GWD-5 across the mire is, as for the individual wells, very small.

**Table 5:**
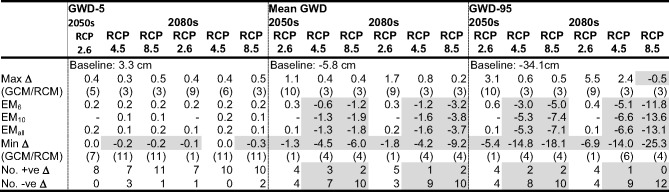
Mean baseline simulated GWD-5, mean GWD and GWD-95 (cm above ground) across all MIKE SHE grid cells that are within the area defined as mire; maximum, ensemble mean (EM) and minimum changes in these groundwater metrics (cm) across GCM/RCM pairs for each climate change scenario (pairs responsible for the extreme changes); and frequency of positive and negative changes in metrics. Shaded cells indicate reductions in metrics compared to the baseline and where the majority of pairs project declines in the value of the metrics

Supplementary Materials Figures SM4.1–SM4.3 map baseline GWD-5 and changes in GWD-5 for each scenario including ensemble means in both the 2050 s and 2080 s for RCP2.6, RCP4.5 and RCP8.5, respectively. Equivalent results for mean GWD and GWD-95 are shown in Supplementary Materials Figures SM4.4–SM4.6 and SM4.7–SM4.9, respectively. The last set of these figures illustrate inter-pair variability in changes in low summer water tables (GWD-95) as well as progressively larger reductions in these levels with increasing radiative forcing. Despite variability in the absolute magnitude of changes, there are some relatively consistent spatial patterns. For example, some of the smallest changes are projected in patches within the central mire immediately downstream of Puy Rond and along the narrow band bordering the stream in the lower catchment where under baseline conditions GWD-95 is close to, and in some areas, above the ground. In contrast, foci of some large changes in GWD-95 are around the upstream limits of the mire, especially within the narrow upslope finger-like extensions towards its south-eastern margins, as well as the small, isolated patch of mire in the west. These areas experience some of the largest declines for those pairs projecting widespread reductions in GWD-95 (e.g. pairs 4 and 6 for RCP4.5 and RCP8.5 - Supplementary Materials Figures SM4.8 and SM4.9) as well as being a focus of increases in GWD-95 for other pairs (e.g. many pairs for RCP2.6 - Supplementary Materials Figure SM4.7), including when declines are projected across much of the mire (e.g. pairs 2 and 3 for RCP8.5 - Supplementary Materials Figure SM4.9). Spatial variations in the magnitude and direction of changes in mean GWD broadly follows those for mean GWD-95 with the concentration of the largest changes (mostly declines) around the upstream mire margin being most obvious (Supplementary Materials Figures SM4.4–SM4.6). Variability in the magnitude of changes in mean GWD-5 across the mire for an individual scenario is much smaller than for mean GWD and especially mean GWD-95 (Supplementary Materials Figures SM4.1–SM4.3). In some cases (e.g. pairs 4 and 11 for RCP4.5, pairs 6, 7 and 12 for RCP8.5) it is possible to differentiate increased GWD-5 in association with the channel flowing to the south of Puy Rond and leaving the largest contiguous area of mire but in absolute terms changes are still small.

### Implications of Climate Change on the Distribution of Mire Vegetation

**Fig. 6:**
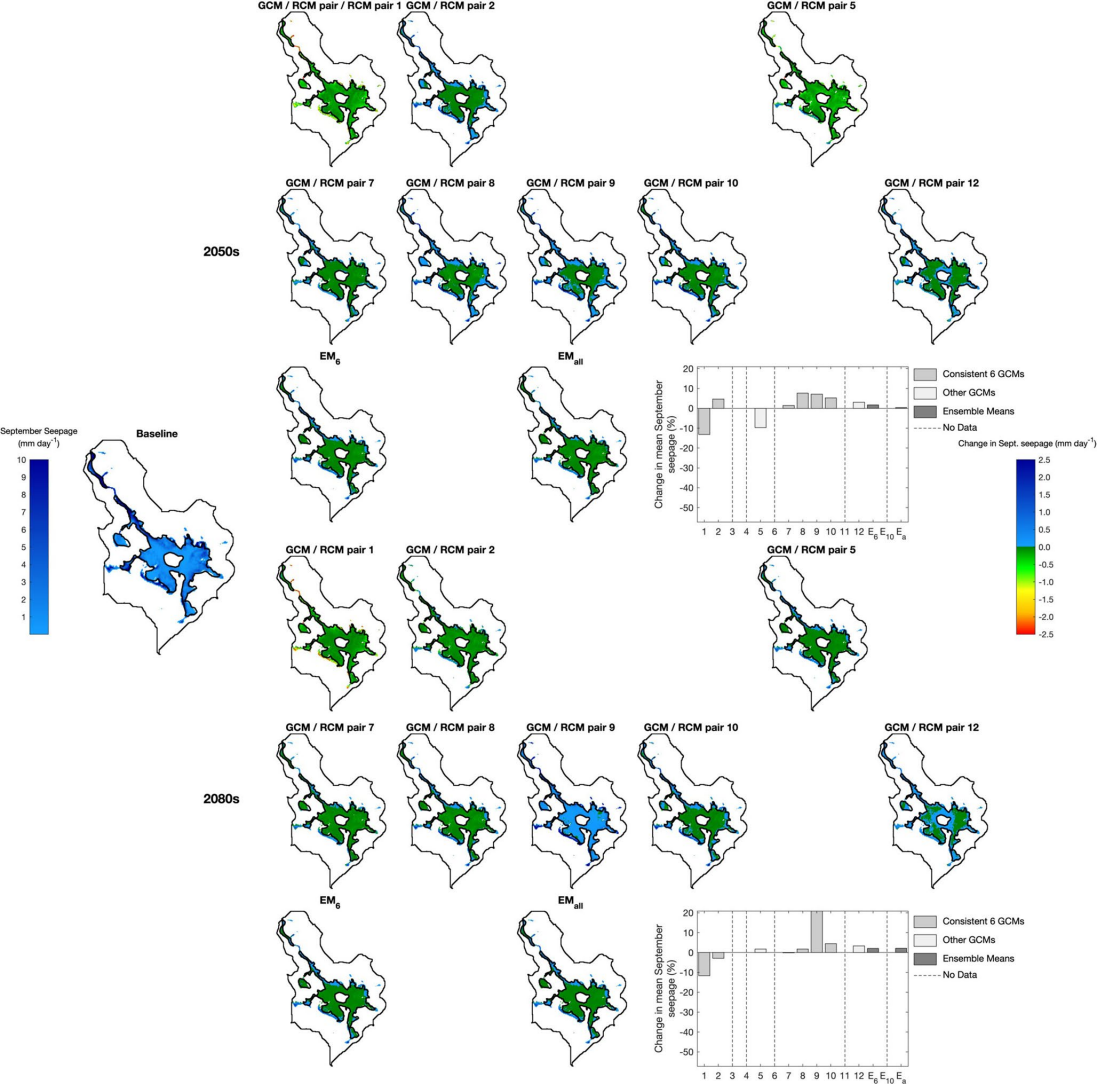
Baseline mean September seepage and scenario changes in mean September seepage within those MIKE SHE grid cells in which this seepage exceeds the threshold (0.005 mm d^− 1^) that best discriminates between mire and non-mire vegetation for RCP2.6 in the 2050 s (top) and 2080 s (bottom). Note that a consistent colour ramp is used for changes in seepage across Figs. 6, 7 and 8 to facilitate comparisons between results for different RCPs. Bar graphs summarise changes from the baseline in the average mean September seepage across the cells that for an individual scenario exceed the threshold. Different shading is used to differentiate the six GCM/RCM pairs that provide data for all RCPs, those pairs that do not provide data for all RCPs as well as the ensemble means. Vertical dashed lines indicate when a particular GCM/RCM pair does not provide data for this RCP. The y-axis ranges for the bar graphs are the same in Figs. 6, 7 and 8

**Fig. 7:**
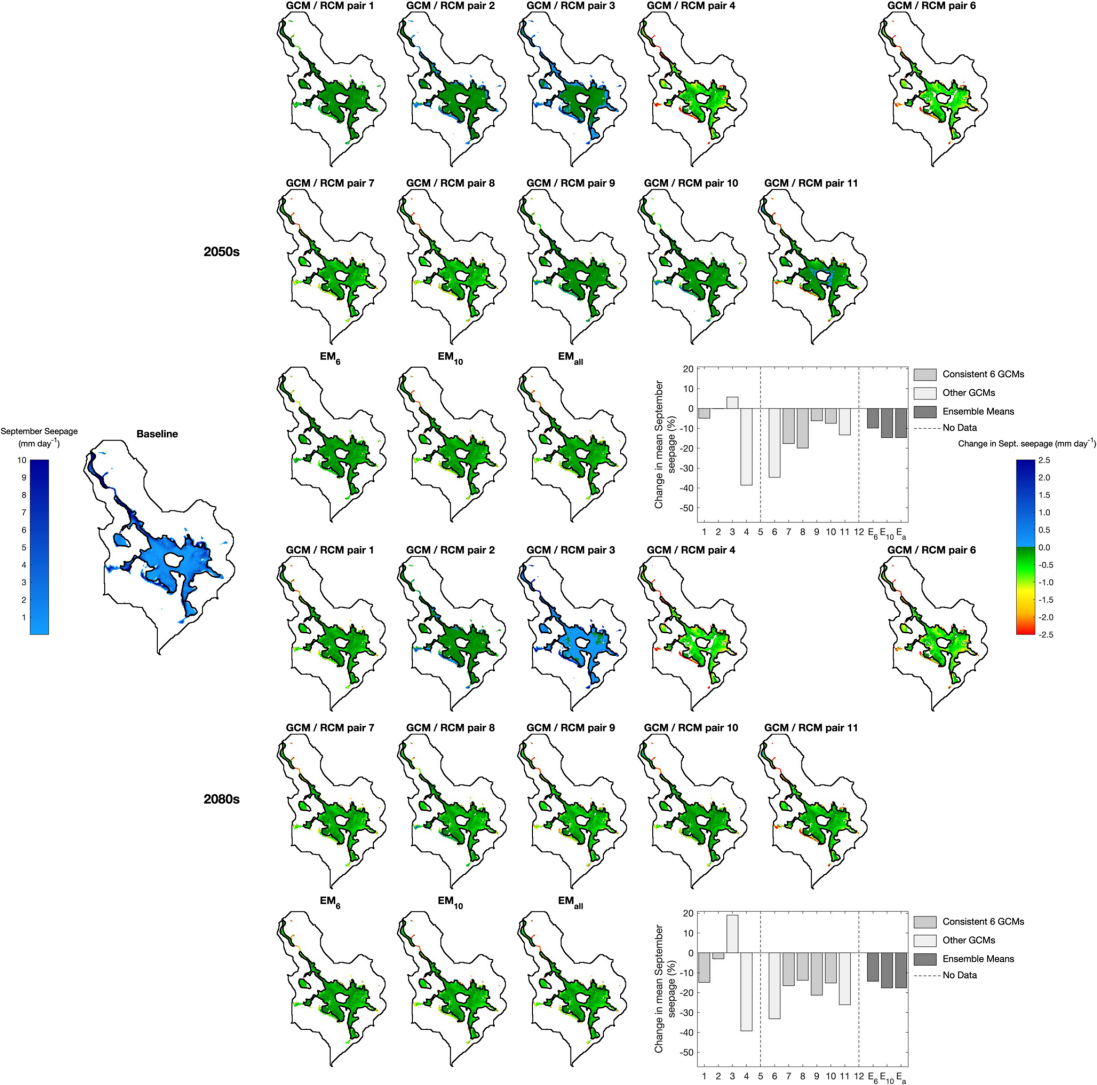
Baseline mean September seepage and scenario changes in mean September seepage within those MIKE SHE grid cells in which this seepage exceeds the threshold (0.005 mm d^− 1^) that best discriminates between mire and non-mire vegetation for RCP4.5 in the 2050 s (top) and 2080 s (bottom). Figure details are as specified for Fig. 6

**Fig. 8:**
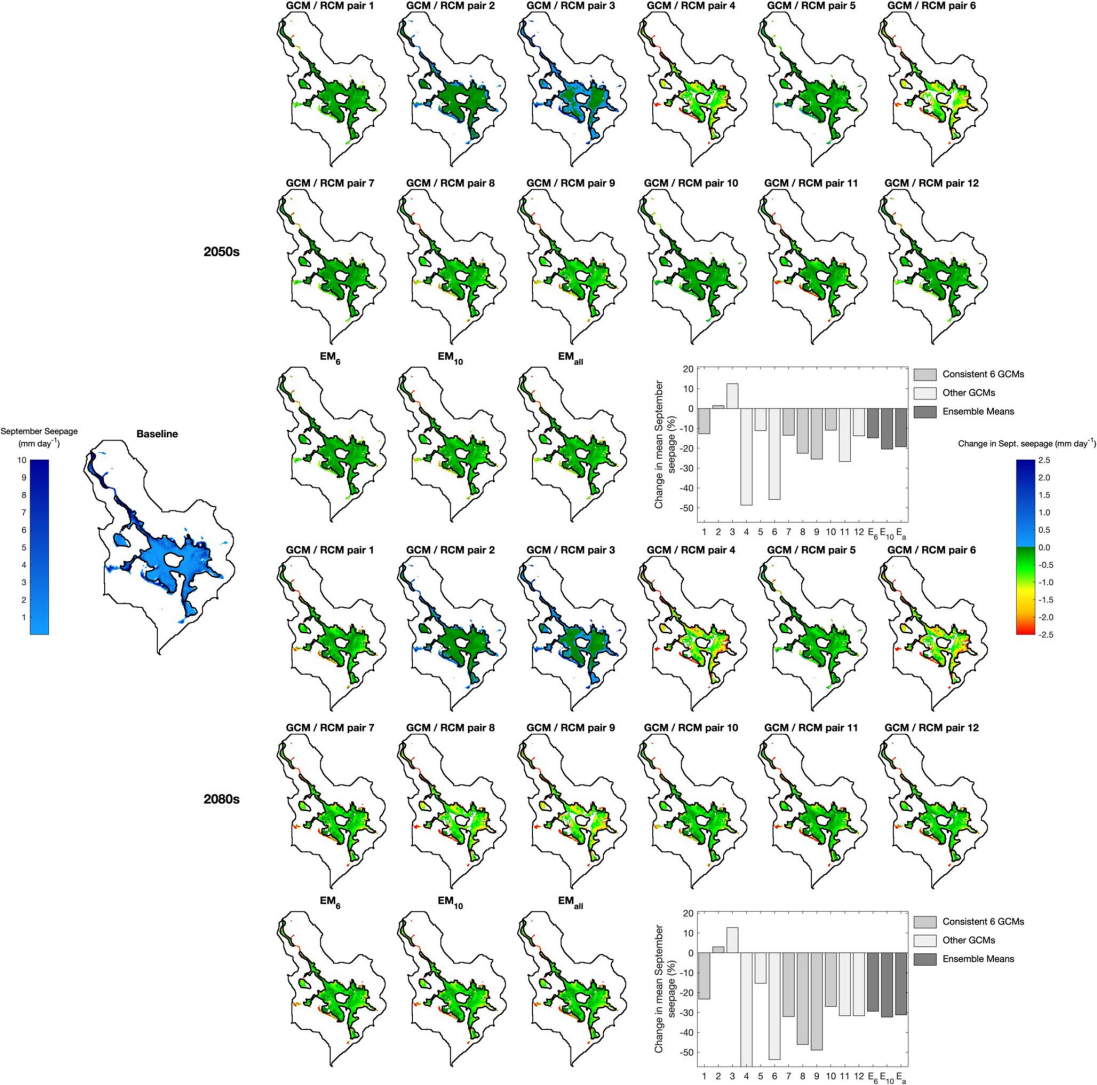
Baseline mean September seepage and scenario changes in mean September seepage within those MIKE SHE grid cells in which this seepage exceeds the threshold (0.005 mm d^− 1^) that best discriminates between mire and non-mire vegetation for RCP8.5 in the 2050 s (top) and 2080 s (bottom). Figure details are as specified for Fig. 6

Table 6 summarises the climate change impacts on the area in which mean September seepage exceeds the threshold for mire vegetation (0.005 mm d^− 1^) and the mean September seepage across these cells (i.e. different numbers of cells feature in these calculations). The corresponding results for all climate change scenarios are provided in Supplementary Materials SM5. Figures 6, 7 and 8 map cells where the threshold seepage is exceeded for the baseline and each scenario for RCP2.6, RCP4.5 and RCP8.5, respectively. For the baseline, the absolute mean September seepage for each cell is indicated whilst changes in seepage from the baseline are displayed for the scenarios. The corresponding analysis using the 0.166 m groundwater depth threshold is presented in Supplementary Materials SM6 and demonstrates very similar trends to those described below.

**Table 6:**
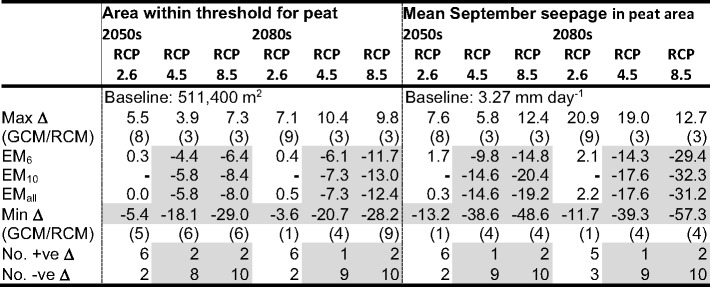
Baseline area (m^2^) in which simulated mean September seepage from the saturated zone to the surface exceeds the threshold (0.005 mm d^− 1^) that best discriminates between mire and non-mire vegetation; baseline mean September seepage (mm day^− 1^) within these areas; maximum, ensemble mean (EM) and minimum changes in these metrics (%) across GCM/RCM pairs for each climate change scenario (pairs responsible for the extreme changes); and frequency of positive and negative changes in metrics. Shaded cells indicate reductions in metrics compared to the baseline and where the majority of pairs project declines in the value of the metrics

Across the 60 combinations of RCPs, time slices and GCM/RCM pairs, the area in which the September seepage threshold is exceeded declines in 41 (68.3%) cases. As for changes in groundwater table depth, declines become more numerous with degree of radiative forcing. Declines for RCP2.6 are projected by just two of the eight (25%) pairs in both time slices with inter-pair range varying by 10.9 and 10.7 percentage points in the 2050 s and 2080 s, respectively. Very small increases for the ensemble means are equivalent to, at most, just 24 MIKE SHE grid cells. Of the ten RCP4.5 pairs, eight and nine (80% and 90%) project declines in the 2050 s and 2080 s, respectively. Inter-pair range of changes in the 2050 s doubles (22.0 percentage points) and in the 2080 s is almost three times larger (31.1 percentage points) than those for RCP2.6. EM_6_ projects declines of 4.4% (2050s) and 6.1% (2080s) with those for EM_10_ being slightly (< 1.5 percentage points) larger. Declines are larger still for RCP8.5 and for EM_6_ are equivalent to 6.4% and 11.6% for the two time slices, respectively (EM_10_ and EM_all_ project slightly larger (< 2%) declines). Of the 12 RCP8.5 pairs, ten (83.3%, both time slices) project declines in area. The range of changes spans 36.2 and 38.0% points in the 2050 s and 2080 s, respectively.

Inter-GCM/RCM pair variations in changes in the area in which the September seepage rate threshold is exceeded, as well as the pairs that account for the extremes of these ranges, follow the trends for mean and, in particular, low water table levels. For example, large declines in area for RCP4.5 and RCP8.5 are projected by pairs 4 and 6 (joined by pair 9 for RCP8.5 in the 2080 s) echoing results for GWD-95. Conversely, the largest increases for these two RCPs are projected by pair 3 followed by pair 2, again matching trends in GWD-95. Direct comparison of changes for these two RCPs with those for RCP2.6 is impacted by absence of data for pairs 3, 4 and 6 in the latter RCP. The largest declines in area for RCP2.6 are for pairs 1 and 5 which again project large reductions in mean GWD and GWD-95. There is also general agreement between pairs projecting the largest gains in area and increases in mean GWD and GWD-95 for RCP2.6 (especially in the 2080 s when pair 9 projects notably larger increases compared to other pairs).

Gains in the area where mean September seepage exceeds the mire threshold tend to be restricted to edges of areas where the threshold is exceeded for the baseline (although given the small extent of these increases, they are often difficult to discern; Figs. 6, 7 and 8). GCM/RCM pair 3 for RCP4.5 in the 2080 s (Fig. 7), associated with the largest total increase across all scenarios, best demonstrates upslope extension of narrow finger-like zones where the seepage threshold is exceeded for these few scenarios. It is in these areas, which experience some of the largest declines in groundwater level, where September seepage no longer exceeds the mire threshold in most scenarios, especially those with small declines in overall total area. Some relatively small areas within the main mire experience reductions in seepage so that it is also below the threshold. However, this is largely restricted to scenarios with the largest declines in total area, in particular pairs 4 and 6 for RCP4.5 and RCP8.5 (both time slices) but also pairs 8 and 9 for RCP8.5 in the 2080 s (Figs. 7 and 8). In these cases, continuous areas where seepage no longer exceeds the threshold are focussed immediately above and below Puy Rond, in a lobe of the main mire towards its western boundary and in the largest, isolated patch of mire in the west. In some instances, the zone in which seepage is below the threshold extends from Puy Rond to the margins of the mire dividing the continuous area of mire under baseline conditions into sections.

Declines in mean September seepage across the MIKE SHE cells in which the mire seepage threshold is exceeded dominate projections (43 or 71.7% of the 60 RCP, time slice and GCM/RCM pair combinations). The numbers of pairs projecting declines or increases for individual RCP/time slice combinations is almost identical to those for changes in the area in which the threshold is exceeded (Table 6). Inter-pair range of changes in mean seepage increases with radiative forcing and more distant time slice with this being predominately due to larger declines. In the 2050 s this range varies between 20.8 percentage points for RCP2.6 to 61.1 percentage points for RCP8.5. The corresponding values for the 2080 s are 32.6 and 69.9 percentage points. In most cases the same pairs account for the extremes of these ranges as those for change in area. The ensemble means project small increases in mean September seepage for RCP2.6 (e.g. EM_6_ 1.7% and 2.1% in the 2050 s and 2080 s, respectively). Declines are projected for higher radiative forcing; for EM_6_ in the 2050 s these are equivalent to 9.8% and 14.8% for RCP4.5 and RCP8.5, respectively, growing to 14.3% and 29.4% in the 2080 s (those for EM_10_ and EM_all_ are 2–6 percentage points larger, Table 6). Under baseline conditions, the largest seepage rates are simulated around the mire margin and these areas are projected to experience some of the largest changes (both increases and more commonly decreases) under climate change (e.g. contrast results for pairs 3 and 4 for RCP4.5 and RCP8.5; Figs. 7 and 8).

## Discussion

### Projected Climate Changes and Their Uncertainty

Future climate projections and their hydro-ecological impacts are characterised by uncertainty (e.g. IPCC 2018; Do et al. 2020; Douville et al. 2021; Lee et al. 2021). The ‘cascade of uncertainty’ (Wilby and Dessai 2010) recognises that uncertainties are associated with different emissions scenarios, alternative process representations and parameterisation schemes within GCMs, and GCM downscaling techniques, with similar process/parameterisation issues if downscaling uses RCMs (Hattermann et al. 2018). Alternative hydrological models forced with perturbed climate introduce further uncertainty (e.g. Haddeland et al. 2011; Thompson et al. 2013; Chan et al. 2020) as do the methods that translate these impacts to ecological responses (e.g. Thompson et al. 2021a).

GCM-related uncertainty frequently dominates uncertainty in hydrological projections (e.g. Prudhomme and Davies 2009; Vetter et al. 2015; Krysanova et al. 2017; Do et al. 2020). This uncertainty can be represented using ensembles of climate change projections derived from multiple climate models forced with the same emissions scenarios. DRIAS-2020 provides such an ensemble for three RCPs for the whole of France over the course of the 21 st Century (Météo-France 2020). There are, however, issues related to the number of GCM/RCM pairs for which projections are available. The largest number of pairs for an individual RCP (12 for RCP8.5) is smaller than the ensemble sizes used in some hydrological impact assessments, including a number reviewing implications for wetlands (e.g. Ho et al. 2016; Thompson et al. 2017a, 2021b; Rahman et al. 2020). Initiatives such as the Coupled Model Intercomparison Project (CMIP) include many more GCMs so that the overall range of projected climatic changes for the Dauges catchment might be larger than is represented by DRIAS-2020. The coupled GCM/RCM approach adopted by DRIAS-2020 does, however, produce projections at the fine spatial resolutions required for catchment-scale hydrological impact studies especially when catchments are much smaller than GCM cell sizes (e.g. Jenkins et al. 2009; Lowe et al. 2018; Nolan and Flanagan 2020). The 60 (78 with ensemble means) scenarios used herein represent a trade-off between a suitably large ensemble and appropriate spatial resolution.

A related issue is the different numbers of GCM/RCM pairs for the three RCPs. As the size of an ensemble declines, the potential for the ensemble mean to be biased by one or more individual members increases as does the likelihood that the ensemble does not represent the full range of projected changes (e.g. Knutti et al. 2009). For example, RCP2.6 lacks some pairs (4, 6 and 11) that project declining annual precipitation for the other two RCPs. Although there is some consistency in the pairs producing the largest decreases and increases in precipitation, it cannot be automatically assumed that these three pairs would, if data were available, project declines for RCP2.6. It is, however, possible that increasing mean annual precipitation projected by the ensemble means for this RCP are larger than they would be if data were available for all GCM/RCM pairs with implications for simulated hydro-ecological changes. Our approach of evaluating alternative ensemble means based on all of the available pairs or a consistent subset of pairs across the three RCPs facilitates comparison of simulated changes with different radiative forcing as well as encompassing the full range of changes projected by the DRIAS-2020 dataset. Whilst there are differences in results for EM_6_, EM_10_ and EM_all_, they are in most cases relatively small suggesting bias due to different numbers of GCM/RCM pairs is present but not substantial.

Notwithstanding these issues, the perturbed climate data demonstrates greater uncertainty in precipitation changes compared to evapotranspiration, echoing results of other climate change impact studies (e.g. Arnell and Gosling 2013; Ho et al. 2016; Thompson et al. 2017a, b; Rahman et al. 2020). In the case of annual precipitation, this uncertainty includes both magnitude and direction of change although most (45 of 60) GCM/RCM pairs project increases with declines being restricted to higher radiative forcing scenarios. This drives the decline in the magnitude of increases projected by the ensemble means from RCP2.6 to RCP8.5 although they are all in single figures in percentage terms. Greater inter-pair range of changes in annual precipitation with magnitude of radiative forcing and more distant time slice replicates findings from earlier studies (e.g. Jobst et al. 2018; Chan et al. 2020; Thompson et al. 2021a). Similar trends are evident for annual reference evapotranspiration, although the size of these ranges (in absolute and percentages terms) is smaller than for precipitation, again replicating previous findings (e.g. Singh et al. 2010; Ho et al. 2016). Consistent increases in annual reference evapotranspiration is supported by many climate change impact studies and is largely driven by elevated temperature (e.g. Kingston et al. 2009; Thompson et al. 2009; 2013; Jiménez Cisneros et al. 2014; Ho et al. 2016; Chan et al. 2020; Emiru et al. 2022). Increasing evapotranspiration can offset increases in precipitation with implications for hydrological changes (e.g. Thompson et al. 2009, 2021b; House et al. 2016a; Chan et al. 2020). This is reflected in our reported increases in the number of GCM/RCM pairs projecting declines in annual net precipitation compared to annual precipitation such that nearly half (2050s) and half (2080s) of the pairs project declines for RCP4.5 whilst for RCP8.5 a majority project declines. Declines for the ensemble means are largely restricted to RCP8.5.

Enhanced climatic seasonality for the Dauges catchment reflects the dominant trend in projections for northwest Europe (e.g. Buttstädt and Schneider 2014; Tabari et al. 2015; Aalbers et al. 2018; Met Office 2021; Christidis and Stott 2022). This is, in particular, driven by projected increases in winter precipitation which characterise most GCM/RCM pairs and the ensemble means. In common with earlier studies (e.g. Thompson 2012; House et al. 2016a; Thompson et al. 2009, 2023), and replicating results for annual precipitation, the range of changes in winter precipitation increases into the future and with radiative forcing. This also applies to changes in summer precipitation which in many cases is projected to decline, further enhancing seasonality. Whilst year-round increases in evapotranspiration for most pairs offset some increases in winter precipitation, it has a particularly notable impact in summer when evapotranspiration increases are largest, and precipitation is relatively small. It is responsible for the increased incidence of negative net precipitation, again replicating other European wetland-based climate change impact assessments (e.g. Thompson et al. 2009, 2017b). A caveat to these climate changes for the Dauges catchment is that, as noted above, whilst the delta factor approach upon which the DRIAS-2020 projections are based is widely used (e.g. Arnell and Reynard 1996; Ho et al. 2016; Chan et al. 2020), and is common to other national climate projection (Jenkins et al. 2009; Lowe et al. 2018), it does not include changes in factors such as rainfall intensity and number of wet days. Projected increases in precipitation intensity and extreme rainfall or an increase in the duration of dry periods (e.g. Douville et al. 2021; Markonis et al. 2021; Martel et al. 2021; Wasko et al. 2021; Rahmani and Fattahi 2024) could, for example, exacerbate some of the hydrological changes identified in our results and discussed below.

### Stream Discharge Projections and Their Implications

Increasing mean stream discharges dominate our projections. In common with previous studies (e.g. Gosling et al. 2017; Chan et al. 2020; Thompson et al. 2023), for a given time slice the inter-scenario (GCM/RCM pair) range of change increases with magnitude of radiative forcing driving declines in the size of increases for the ensemble means (just above/below double figures in percentage terms above/below the mire for RCP2.6, a few percent/close to no change, and for EM_10_ and EM_all_ at Pont de Pierre declines of a few percent, for RCP8.5 in the 2080 s). Increasing mean discharge, albeit small in magnitude, for ensemble means counters the dominance of reported climate change-induced flow reductions for some French rivers (Boé et al. 2009; Habets et al. 2013; although there is some uncertainty in direction of change, Dorchies et al. 2014). These studies focus predominantly on large river basins including the Rhone, Loire and Seine. Annual baseline precipitation over the Dauges is relatively high (1267 mm) given its upland location whilst temperatures, and hence baseline annual evapotranspiration, are relatively low (692 mm/54.6% of annual precipitation) contributing to substantially positive baseline annual net precipitation (575 mm). In contrast, larger river basins within the region extend over lower elevations with potentially lower mean catchment precipitation, relatively warmer catchment mean temperatures and so higher evapotranspiration. Increases in precipitation for the same GCM/RCM pairs employed in this study would, in absolute terms, likely be smaller and increases in evapotranspiration larger accounting for the more numerous declines in mean discharge reported in earlier studies. These same factors account, at least in part, for differences in climate change signals for mean discharge established using MIKE SHE/MIKE 11 models of upland (SW Scotland, increases in mean discharge; Thompson 2012) and lowland (Eastern England, dominance of declines; Thompson et al. 2017b) UK catchments.

Our results demonstrate a dominant climate change-driven increase in seasonality of stream flow (widening of the difference between Q5 and Q95), another feature of previous climate change impact assessments for French rivers (e.g. Boé et al. 2009; Habets et al. 2013; Dorchies et al. 2016) as well as others from across northwest Europe (e.g. Middelkoop et al. 2001; Shabalova et al. 2003; Thodsen 2007; de Wit et al. 2007; Johnson et al. 2009; Kay 2021). The majority of GCM/RCM pairs (> 85% across two time slices, three RCPs, and two gauging stations) and all ensemble means project increases in both Q5 and winter discharges. Ensemble mean increases for Q5 are in the range 10–16% above the mire and 6–10% below it. Some individual pairs project increases that are twice as large. At the other extreme, the majority of pairs project declines in Q95 and summer discharges, although this trend is less equivocal (> 65%). Indeed, increases in Q95 of 3–6% are projected by EM_6_ for RCP2.6 with only small differences between the two time slices. Ensemble means project declines with higher radiative forcing (4–9% for RPC4.5, upper end of this range associated with the 2080 s; 11% for RCP8.5 in the 2050 s and as high as 27% and 53% for the 2080 s above and below the mire, respectively with substantial inter-pair ranges).

Whilst the Dauges catchment is small, it is representative of many catchments in the Massif Central which are the headwaters for larger rivers (Duranel 2015). Given the importance of upland areas and their peatlands for catchment-wide hydrology and water resources (e.g. Hubacek et al. 2009; Xu 2018), replication of the trends for high and low flows identified in this study over a wider area have potential implications. Higher peak flows accord with observed and projected increases in riverine flood intensity, frequency and damage over large parts of Europe (e.g. Kundzewicz et al. 2010; Blöschl et al. 2019). If replicated across adjacent catchments, the impact could be higher flood discharges further downstream including the Vienne River with the potential for damage to properties and other infrastructure as witnessed during the spring 2024 floods (La Nouvelle République 2024; Ouest France 2024). Other impacts could include the hydromorphological changes reviewed by O’Briain (2019) in the context of widespread increases in winter floods throughout Europe.

Replication of our projected declines in summer low flows across the Massif Central will enhance drought conditions with ecological impacts further compounded by water quality effects including reduced dilution as well as warmer water temperatures (e.g. Whitehead et al. 2009; Laizé et al. 2014; O’Briain 2019). These changes could exacerbate the already highly degraded nature of many French rivers (Oberdorff et al. 2002; Arevalo et al. 2020; Bayramoglu et al. 2020). Declining summer flows could exacerbate existing water shortages and increase competition for water between different human uses and the environment. Droughts across Nouvelle-Aquitaine have severely impacted water supply to several communities in the Monts d’Ambazac (Meunier 2001). In recent cases this has required importation of drinking water via tankers or pumping from the Vienne River which itself has experienced extreme low flows (Populaire du Centre 2022; TF1 2023). Related water resource concerns include implications for electricity generation with droughts in summer 2022 cutting nuclear power generation as river flows and temperatures approached limits set for cooling water discharges (Guardian 2022; Vie Publique 2023). Power stations affected included the Civaux plant on the Vienne River for which the annual volume of water pumped for cooling is already close to the maximum limit (Cour des Comptes 2023).

Our results show some reasonably consistent differences in the relative magnitude of changes in high and low stream flows upstream and downstream of the mire. These could be attributed to some buffering of climate change by the peatland and wider catchment (Tóth 2009). The role of peatlands within catchment hydrology including controlling river flow variability and maintaining baseflow is referred to in several studies (see the review of Bullock and Acreman 2003). That being said, their role as “sponges” that store and then release water (attributed to Turner 1757), is a simplification that has been contradicted by field evidence (Evans et al. 1999; Holden 2005) with their flood mitigation role often overstated (Simonovic and Juliano 2001). Notwithstanding these issues, some research has demonstrated that peatlands fed by local or regional groundwater, such as within the Dauges catchment, can provide stores that maintain streamflow (Levinson et al. 2014) with water originating from the peat being particularly important during low flow periods (Kværner and Kløve 2006, 2008; Bourgault et al. 2014). In addition, low peat permeability may act to regulate groundwater discharge from mineral aquifers to streams (Rossi et al. 2012, 2014). Peatlands could, therefore, provide buffers within catchments, offering some resilience to recharge variations due to climate change. When our results project declines in low flows (Q95) above the mire (Rocher), the corresponding reductions further downstream (Pont de Pierre) are predominantly smaller (by on average c.12 percentage points). Whilst GCM/RCM pairs projecting reduced low flows are also associated with declining water table levels, if groundwater levels are still higher than stream water levels, the MIKE SHE/MIKE 11 coupling will simulate exchange from the saturated zone to the stream offsetting some declines in stream flow. Downstream declines in high flows (Q5) are also predominantly smaller than those upstream suggesting that this effect could persist at other times although upstream-downstream differences are much smaller (c.2 percentage points on average). When Q95 and Q5 increase above the mire, corresponding gains downstream are smaller albeit only marginally so (c.2 and c.5 percentage points on average, respectively). This could again indicate some buffering by the catchment and mire with a greater proportion of stream flow deriving from groundwater with its longer residence time. Peat in low gradient areas may intercept groundwater, especially around the mire’s margins (discussed below) and surface runoff from the catchment that would otherwise reach the stream. In addition, high stream flows and hence MIKE 11 water levels compared to water tables in adjacent MIKE SHE grid cells could enhance stream to saturated zone exchange in accordance with flow reversal under climate change described by Levison et al. (2014).

### Climate Change and Peatland Groundwater Levels

Increased seasonal variations in peat groundwater levels dominate projected changes and echo earlier assessments of climate change impacts on other wetlands (e.g. Acreman et al. 2009; Thompson et al. 2009; Herrera-Pantoja et al. 2012). Unlike stream discharge, enhanced seasonality is driven by declining summer water tables instead of a combination of elevated highs and reduced lows. Under baseline conditions, simulated water table within the mire is just below, at, or just above the ground surface for extended periods (i.e. November–March and a month or two longer for some well locations) resulting in GWD-5 values which are, on average, just (c.3 cm) above the surface. Whilst the vast majority of scenarios project increasing GWD-5 (80.8% of the 240 time slice, RCP, GCM/RCM pair and four well combinations/88.3% of the equivalent 60 combinations for mean GWD-5 across the mire), changes are very small (overall range across the 240 combinations is −0.23–0.43 cm). Such small changes, despite increased winter precipitation for many scenarios, result from simulation of runoff processes described in the literature. High water tables ensure that saturation excess overland flow is a dominant process, especially in winter (e.g. Evans et al. 1999; Holden and Burt 2002a; Kværner and Kløve 2008). Relatively small amounts of rainfall, potentially supplemented by upland runoff and groundwater upwelling, cause the water table to rise to the surface, with rainfall on these saturated areas running off (Devito et al. 1996; Holden and Burt 2003; Holden 2005). When simulated by MIKE SHE, this overland flow follows the topography before being intercepted by MIKE 11 and removed as stream flow. Given the relatively small distances across the mire to the stream, combined with gentle topography across the mire, this process happens rapidly with little opportunity for accumulation of surface water. These processes account for similarly very small changes in peak water tables despite elevated winter precipitation in MIKE SHE/MIKE 11 climate change simulations of lowland wet grasslands (Thompson et al. 2009) and small floodplains (Thompson et al. 2017b, 2023).

Simulated baseline water table levels decline through spring reaching their lowest in summer as precipitation is progressively exceeded by evapotranspiration. Whilst on average across the mire MIKE SHE cells GWD-95 equals just over 34 cm below the ground surface, there are spatial variations in these low water tables. Some larger drawdowns occur close to stream channels (e.g. patches south and east of Puy Rond that are coincident with channels; Supplementary Materials Figures SM4.7–SM4.9). This may support the assertion that channels crossing the mire locally drain the peat (e.g. Gilman 1994), potentially offsetting some declines in summer stream flows discussed above (although there is potential for underestimation of stream stage, and hence backwater into the peat, by the kinematic routing method (Duranel 2015)). Elsewhere, baseline GWD-95 is closer to the ground surface and in some cases above it. This is particularly evident around the mire’s margins and along the stream in the lower section of the catchment. Some of the largest baseline summer seepage rates are simulated in these areas (Fig. 8) and Duranel et al. (2021) argued that they exemplify edge-focussed discharge (Richardson et al. 2001) or margin seepage (Hare et al. 2017). This occurs where groundwater upwelling is enhanced due to the break of slope between the hillside and valley bottom that, in turn, impacts water table gradients (see Winter 1988). These groundwater influxes can be critical in maintaining water table levels and surface saturation in upland wetlands (Devito et al. 1996).

Many areas of shallow baseline seasonal low water tables and high groundwater upwelling are the focus of the largest changes in these characteristics with climate change. This includes the largest increases for scenarios associated with wetter conditions as well as, most notably, the largest declines for scenarios projecting reductions in GWD-95 and September seepage. The latter are in the majority (47 [43] or 78.3% [71.7%] of the 60 time slice, RCP, GCM/RCM pair combinations project declines in mean GWD-95 [September seepage] across the mire). The number of pairs projecting declining GWD-95 and September seepage, the size of the drying trends and the inter-pair range of changes increase with radiative forcing and more distant time slice. In some cases, most notably RCP 8.5, the duration of summer drawdown is extended delaying the period when water tables are close to the surface, a trend exhibited in other wetlands under climate change (Thompson et al. 2017b). Changes in GWD-95 for the ensemble means for RCP2.6 are very small with the direction of change varying between locations (for EM_6_ they range between − 0.3 cm and 4.7 cm for the four wells reviewed with mean increases of 0.6 cm (2050s) and 0.1 cm (2080s) across the mire). Declines in single (cm) figures are projected for RCP4.5 in both time slices and RCP8.5 in the 2050 s whilst for the last RCP in the 2080 s declines exceed 10 cm (11.8 cm on average and at some wells over 20 cm). Whilst relatively small, these declines approach the upper end of those simulated for some UK wetlands (Thompson et al. 2009, 2017b, 2023; House et al. 2016a). These earlier studies involved sites in lowland catchments where summer net precipitation was already more negative than the Dauges catchment and became even more so under climate change thereby accounting, at least in part, for larger declines in the low water table levels.

### Additional Sources of Uncertainty in Climate Change Impacts

Hydrological changes simulated by the model assume that catchment rainfall-runoff characteristics are unchanged from the baseline, a common approach in model-based climate change studies (e.g. Döll and Zhang 2010; Gosling et al. 2017; Hudson and Thompson 2019; Do et al. 2020; Rahman et al. 2020; Thompson et al. 2023). Climate change, combined with other factors including agroforestry practices, may modify catchment characteristics. Vegetation and land use exert important controls on runoff and groundwater recharge (e.g. Brown et al. 2005; Monger et al. 2022). Drier conditions under climate change, most likely in summer, may reduce forest production (Petr et al. 2014), offset potential increased productivity due to elevated atmospheric CO_2_ (Warren et al. 2011) and enhance tree mortality (Ciais et al. 2005). Impacts will vary between species and could contribute to shifts in community structure with implications for the Dauges catchment and its broadleaf woodland. A preliminary evaluation of hydrological changes due to land cover change demonstrated that conversion of broadleaf woodland to conifer plantations, a widespread practice since the 20th Century (Derrière et al. 2013; Dodane 2009), would enhance interception and evapotranspiration (Duranel 2015; Duranel et al. 2017). This would reduce mean stream flows and summer low flows. Water table levels would drop with the largest declines (up to 1 m) occurring around the mire margin, the same locations experiencing the largest changes in the current study. Conversely, woodland removal and replacement with grassland or heath would increase streamflow and groundwater upwelling although, as in the current study, increases in groundwater levels would be small since baseline water tables are close to the surface. Further assessments of the implications of catchment vegetation change, in isolation or combined with climate change, would be a useful extension of research using the Dauges MIKE SHE/MIKE 11 model.

Further uncertainty in the hydrological impacts of climate change include modifications to peat hydraulic properties (Holden et al. 2006). Declining water tables following drought or artificial drainage have caused peat shrinkage and creation of macropores that may develop into subsurface pipe networks (e.g. Holden and Burt 2002b; Holden 2005). Water level drawdowns can also increase bulk density and reduce hydraulic conductivity (Whittington and Price 2006; Loisel and Gallego-Sala 2022). Longer drawdowns may lead to oxidative wastage and subsidence of the peat, in turn producing irreversible changes to the mire’s topography (Kennedy and Price 2005). Such knock-on impacts are not represented within our climate change simulations. Whether, and to what extent, our simulated changes would modify peat hydraulic properties and volumesd requires additional research. Whilst empirical and experimental studies that have assessed the impacts of changing water levels on peat hydraulic properties may act as surrogates for climate change (Holden 2005; Whittington and Price 2006), translating modified water tables to alternative hydraulic conductivity values would introduce considerable uncertainty. This could be represented using an ensemble of potential values including alternative spatial patterns of change (e.g. Christiaens and Feyen 2001) but would increase computational demands if the full ensemble of climate scenarios were simulated using multiple MIKE SHE/MIKE 11 models. Similarly, whilst the MIKE SHE bypass flow and drainage options have been used to represent macropores within wetlands (e.g. Thompson et al. 2004; Clilverd et al. 2016), their parameters are usually established via calibration impacting their inclusion and modification in the context of climate-change induced alterations to peat properties.

Modified peat water table regimes within the Dauges mire that are dominated by larger summer drawdowns have potential further implications which would merit research. For example, water table depth and fluctuations exert important controls on rates of peat decomposition, aerobic conditions and carbon storage including CO_2_, dissolved organic carbon (DOC) and CH_4_ release (e.g. Holden 2005; Strack and Waddington 2007; Frolking et al. 2011; Chimner et al. 2017; Loisel and Gallego-Sala 2022). Changes in peatland hydrological conditions across the Massif Central could provide feedback to the global climate system (Strack et al. 2004; Whittington and Price 2006). Repetition of hydrological changes in adjacent catchments could have implications for downstream water quality. Peatlands have been shown to store a range of elements including nitrogen, phosphorous (Devito and Dillon 1993), methylmercury (Branfireun et al. 1996), sulphate (Devito 1995; Devito and Hill 1997), arsenic (Langner et al. 2012) and uranium (Lidman et al. 2012) that could be released with hydrological changes (Owen and Otto 1995; Daniels et al. 2008; Nieminen et al. 2017). Surface water acidification has, in some cases, been attributed to SO_2_ release from upland peatlands during prolonged periods of low water level (Van Dam 1986; Tipping et al. 2003; Eimers et al. 2006) with links to subsequent fish kills (Holopainen and Oikari 1992). Of particular concern in the Dauges area, as well as the wider Monts d’Ambazac, is release of arsenic and radionuclides into water courses (e.g. Franceinfo 2020). Further water quality implications are related to DOC and particulate organic carbon (POC) fluxes that might increase under drier conditions, especially if they change flow paths such as enhancing macropore flows (Evans et al. 2005; Holden 2005).

### Translating Hydrological Changes to Peatland Responses

The approach used to translate hydrological changes to impacts on mire vegetation employs the concept of “plants as hydrologists” (e.g. Wheeler and Shaw 1995a, b) that is to say that distribution of wetland plants reflects variability in hydrological conditions to which they are adapted. Groundwater characteristics including water table depth exert strong controls on plant composition and zonation within many wetlands (Duranel et al. 2007; Toogood et al. 2008; Wheeler et al. 2009; Clilverd et al. 2022) including upland peatlands (Bragazza and Gerdol 1999; Hilbert et al. 2000; Hose et al. 2014). The influence of hydrological conditions can be established via monitoring (e.g. water table depth) within plots of different vegetation species/communities in a single wetland or groups of wetlands (e.g. Asada 2002; Hettenbergerová et al. 2013; Johansen et al. 2018). Hydrological controls can be expressed as desirable water table levels/range of water tables including seasonal variations (e.g. Wheeler et al. 2009) or translated into indices representing physiological controls upon plants with Sum Exceedance Values for aeration stress (SEV_as_; Sieben 1965) being applied to wetlands (Gowing et al. 1998; Thompson et al. 2023). Although distributions of some species within the Dauges mire have been established (Duranel 2015), their hydrological requirements are unknown and subject to ongoing research. The current study therefore used threshold conditions for the presence of mire vegetation established using the baseline model. Whilst very close agreement between the mire boundary and a threshold mean groundwater depth was identified, mean groundwater seepage in September exerted a stronger control. Duranel et al. (2021) suggested that upwelling in the driest month is critical for sustaining mire vegetation. Groundwater-peat fluxes are difficult to measure in the field (Larocque et al. 2016), especially when they vary spatially. Simulated seepage is, therefore, advantageous in establishing controls upon mire distribution and how this distribution might vary under climate change.

The dominant drying trend under climate change, especially in summer, means that the area in which September seepage exceeds the mire vegetation threshold declines in most cases (41 or 68.3% of the 60 combinations). Declines become more numerous with magnitude of radiative forcing and more distant time slice; whilst just two of eight GCM/RCM pairs project declines for RCP2.6 in both the 2050 s and 2080 s, reductions are projected for all but two of 12 RCP8.5 pairs, again for both time slices. The magnitude of declines in area, as well as mean seepage within these areas, and inter-pair range of changes increase with radiative forcing and into the future. Whilst the extent of declines is relatively large for some pairs (approaching a third for some for RCP8.5), ensemble mean declines are in single figures (very small increases of < 2% for RCP2.6) for all but RCP8.5 in the 2080 s (c.12%). This is in line with the suggestion that climate change may not cause widespread mire loss but may impose stress on these environments (Gallengo-Sala and Prentice 2013). Changes of similar magnitude could be expected in other mires within the Massif Central although inevitably there will be uncertainty related to hydrogeological setting which controls groundwater seepage and shallow water tables, critical factors determining peatland hydroecological character (Larocque et al. 2016; Duranel et al. 2021).

Whilst the approach used to infer climate change impacts on mire vegetation is grounded in strong links between hydrological conditions and botanical responses, the binary division between seepage (mean groundwater depth) being suitable for mire vegetation or not is a simplification. It does not enable assessments of the impacts on different species or sub-communities given differential responses of peatland species to changing hydrological conditions (Weltzin et al. 2003; Dieleman et al. 2015; Laine et al. 2021). This could be investigated if species/sub-community specific hydrological thresholds are established (Clilverd et al. 2022; Thompson et al. 2023). Even then, our approach assumes that so long as thresholds are exceeded, conditions are equally favourable when they may only just exceed them with vegetation experiencing stress due to reduced seepage and lower water levels (Gowing et al. 1998; Wheeler et al. 2009). In percentage terms declines in mean September seepage in those areas where it still exceeds the threshold are predominantly larger (by on average over 2.5 times) than the corresponding change in area.

Our results suggest that foci for vegetation change and stress are around the mire boundary, including the finger-like upslope extensions which are projected to experience the largest declines in groundwater depth and seepage. Given their location on the edge of the peatland, this may facilitate encroachment by non-wetland plants (Holmgren et al. 2015; Langdon et al. 2020). Elsewhere, drier conditions have driven colonisation of peat-based wetlands by shrubs or trees (Jukaine et al. 1995; Talbot et al. 2010; Strakova et al. 2012) with this progressing around and just ahead of current vegetation boundaries (Clark et al. 2001; Dovčiak et al. 2015). Deeper roots and denser canopies could promote greater evapotranspiration and interception with positive feedback on drier conditions (Heijmans et al. 2013). In addition, there is potential for differential impacts depending on sequencing of relatively wet vs. relatively dry years. Our approach uses mean September seepage for the period 01/01/2001–31/12/2013. The same mean values could be produced from alternating wet and dry years compared to a number of wet years followed by a sequence of dry years (or vice versa). Different sequencing may have different impacts on the mire including variable implications for individual species/sub-communities and competition between them (Gowing et al. 1997; Langdon et al. 2020). For example, extreme and/or prolonged droughts have caused irreversible desiccation of mosses (Bragazza 2008) and expansion of vascular plants (Gerdol et al. 2008) within peat bogs of the Italian Alps.

There are other climate change-related drivers of potential vegetation change that are not considered using our hydrological change-based approach. For example, if lower water tables increase decomposition rates, nutrient availability could change (Macrae et al. 2013; Wang et al. 2018). The nature of such changes will vary with magnitude of hydrological change and will have variable implications for different plant functional types (Munir et al. 2017). They would also be subject to vegetation feedbacks which are currently poorly understood (see Zhong et al. 2020). Furthermore, whilst increases in temperature are incorporated within scenario evapotranspiration, temperature, combined with atmospheric CO_2_ concentrations, exerts additional direct controls on peatland plant communities (Weltzin et al. 2000; Turetsky 2003; Breeuwer et al. 2008; Heijmans et al. 2013). Higher temperatures have, for example, been shown to reduce the competitive advantage of peat-forming plants, in particular Sphagnum, over vascular plants (Dieleman et al. 2015). With everything else being equal, there is the potential for larger losses of mire vegetation within the Dauges catchment than those reported herein.

## Conclusions

A high spatial resolution coupled MIKE SHE/MIKE 11 model of the Dauges National Nature Reserve, northwest Massif Central calibrated against observations of stream discharge and peat water table depths was employed to assess the hydro-ecological implications of climate change. The ensemble of climate change scenarios employed all 60 of the available GCM/RCM pairs from the DRIAS-2020 dataset for three RCPs (RCP2.6, RCP4.5 and RCP8.5) and two thirty-year time slices centred on the 2050 s and 2080s. Three ensemble means were also employed reflecting different numbers of pairs providing data for the alternative RCPs so that in total 76 scenarios (38 for each time slice) were simulated.

There is some uncertainty in direction of change in mean annual precipitation with 45 of 60 GCM/RCM pairs projecting increases (15 decreases). Declines become more common with higher radiative forcing and inter-pair range of changes increases (e.g. RCP2.6, 2050s: c1–11%; RCP8.5, 2080s: −10–13%). Small increases (5–8% for RCP2.6, 1–5% for RCP8.5) are projected by the ensemble means. All pairs project increasing mean annual evapotranspiration. Magnitude and inter-pair range of these changes increases with radiative forcing and into the future (RCP2.6 in the 2050s: 2–5%, RCP8.5 in the 2080s: 9–18%). Increases for ensemble means are in the range 2–3% for RCP2.6 (both time slices) and between 6% (2050s) and 12% (2080s) for RCP8.5. Enhanced winter precipitation (all but two pairs) exerts a dominant influence on increasing climatic seasonality, further enhanced by declining summer precipitation (smaller majority of pairs). Increasing evapotranspiration throughout the year offsets some of the wetter winter condition but enhances summer drying increasing the incidence of negative monthly net precipitation. The magnitude of seasonal changes and the range of changes across different pairs increases with radiative forcing and more distant time slice.

Stream flow projections are characterised by enhanced seasonality although the mire and groundwater discharge from the catchment might buffer some climate change impacts below the mire. Increased peak flows are particularly common (Q5: 92% and 88% of pairs upstream and downstream, respectively) with increases for the ensemble means varying by 14–15% (8–10%) for RCP2.6 and 8–12% (5–8%) for RCP8.5 above (below) the mire. A smaller majority of pairs project declines in summer low flows (Q95: 67% and 68% of pairs above and below the mire). Whilst the ensemble means project declines in low flows for RCP4.5 (upstream 4–14%, downstream 6–14%, small differences between time slices) and RCP8.5 (upstream 11–24% in the 2050 s and 53–58% in the 2080 s; downstream 11–17% for the 2050 s and 27–31% for the 2080 s), RCP2.6 is associated with small (3–6%) increases above and below the mire. Increases in mean discharge dominate (78 and 77% of pairs upstream and downstream, respectively). Most ensemble means project increases although they decline in magnitude with radiative forcing. Above the mire increases are in the range 12–13% for RCP2.6 (both time slices), and then 4–7% (2050s) and just < 1–2% (2080s) for RCP8.5. The equivalent changes below the mire are slightly smaller (a few percentage points) so that two ensemble means project declines (< 1.5%) for RCP8.5 in the 2080s.

Declines in seasonal low peat groundwater table depths dominate (over 80% of RCP, GCM/RCM pair and well/MIKE SHE grid square combinations project declines in GWD-95). The highest groundwater tables (GWD-5), which under baseline conditions are at or very close to the ground surface, are effectively unchanged. There is a dominant trend towards increased seasonality of water table depths and lower mean water table levels (> 70% of combinations) with the magnitude of changes increasing with radiative forcing and future time slice. Ensemble means project increased mean GWD-95 across the mire of, on average, less than 1 cm for RCP2.6 (both time slices). This switches to declines of 3–5 cm (RCP4.5) and 5–7 cm (RCP8.5), respectively in the 2050 s and 5–7 cm and 12–14 cm for the 2080s. This trend is repeated for mean GWD albeit with smaller changes than for GWD-95. Projected changes in mean GWD and GWD-95 for some individual pairs are larger and inter-pair ranges of change increase from RCP2.6 through to RCP8.5 and from the 2050 s to the 2080 s (ranges of mean change in GWD-95 across the mire are − 5.4–3.1 cm for RCP2.6 in the 2050 s and − 25.3– −0.5 cm for RCP8.5 in the 2080 s). The largest absolute changes in water table depth are around the mire margins, which are locations of edge-focussed discharge from the saturated zone.

The extent of hydrological conditions assumed to support mire vegetation declines for most scenarios. In the case of mean September seepage, declines are projected for 41 of the 60 GCM/RCM pairs. Declines become more numerous as radiative forcing increases; two of eight pairs project declines for RCP2.6 (both time slices), increasing to eight (2050s) and nine (2080s) of ten RCP4.5 pairs whilst declines are projected for ten (both time slices) of 12 pairs for RCP8.5. Ensemble means projects small (< 1%, both time slices) increases for RCP2.6. This shifts to declines of 4–6% (2050s) and 6–7% (2080s) for RCP4.5 with the equivalent ranges for RCP8.5 equalling 6–8% and 12–13%. Declines in the magnitude of mean September seepage averaged across areas in which the mire threshold is exceeded are, in percentage terms, larger than area reductions (10–15% and 14–18% for RCP4.5 in the 2050 s and 2080 s, respectively, 15–20% and 29–32% for RCP8.5). Areas simulated as no longer able to support mire vegetation, and those areas which retain their suitability but undergo the largest declines in seepage, are concentrated around the mire margin.

Whilst results project predominately drier conditions and modest declines in the extent of suitability for mire vegetation, there are sources of uncertainty regarding the future of peat ecosystems in the Dauges catchment and, by extension, the wider Massif Central. These include climate change driven shifts in catchment vegetation and their impacts on runoff and groundwater fluxes, changes in peat hydraulic properties and nutrient status, differential impacts of changing hydrological conditions (as well as temperature and nutrient availability) upon different species and, in turn, impacts on inter-specific competition.

## Supplementary Information

Below is the link to the electronic supplementary material.

Supplementary File 1 (PDF 12.2 MB)

## Data Availability

The datasets generated during and/or analysed during the current study are available from the corresponding author on reasonable request.
