# Supplementary material for: Simulation of the Hydro-ecological Impacts of Climate Change on an Upland Peatland in the Massif Central
Source: Wetlands (Wilmington). 2026 Mar 23;46(4):36. doi: 10.1007/s13157-026-02046-7 (PMC13009095; doi:10.1007/s13157-026-02046-7)
Supplement: Supplementary file 1 — (PDF 12.2 MB) [file 13157_2026_2046_MOESM1_ESM.pdf]

Simulation of the hydro-ecological impacts of climate change on an upland peatland in the Massif Central

Supplementary Materials

Julian R. Thompson[1], Arnaud Duranel[1,2], Emma Keisser[1], Philippe Durepaire[3] and Hervé Cubizolle[2]

1. UCL Department of Geography, University College London, London WC1E 6BT, UK.
2. Jean Monnet University, UMR 5600 CNRS EVS, 42023 Saint-Etienne CEDEX 2, France.
3. Réserve Naturelle Nationale de la Tourbière des Dauges, Conservatoire d'Espaces Naturels de Nouvelle Aquitaine, Sauvagnac 87340 Saint-Léger-la-Montagne, France.

SM1. Projected changes in meteorological forcing

Table SM1.1 presents the projected changes in annual climate variables (mean annual precipitation, reference potential evapotranspiration, net precipitation) as well as the frequency of days when precipitation exceeds evapotranspiration for all of the climate change scenarios including the three ensemble means. These data are used to summarise the projected changes in meteorological forcing for the MIKE SHE / MIKE 11 model within Table 2 of the paper.

Table SM1.1. Baseline mean annual precipitation, annual reference evapotranspiration (ET) and annual net precipitation (mm) over the Dauges catchment and changes in these annual totals (%) projected by each GCM / RCM pair and the three ensemble means (EM) for the three RCP scenarios in the 2050s and 2080s. Number of days when precipitation > ET (i.e. net precipitation is positive) for the baseline and changes (days) for each scenario. Baseline data are for the period 01/01/2001– 31/12/2013. *Shaded cells indicate reductions compared to the baseline.*

		GCM / RCM 1	GCM / RCM 2	GCM / RCM 3	GCM / RCM 4	GCM / RCM 5	GCM / RCM 6	GCM / RCM 7	GCM / RCM 8	GCM / RCM 9	GCM / RCM 10	GCM / RCM 11	GCM / RCM 12	EM_6	EM_{10}	EM_{all}
Mean annual precipitation – Baseline: 1267.0																
2050s	RCP2.6	2.8	6.0	-	-	9.1	-	1.2	3.5	8.3	10.7	-	2.3	5.4	-	8.4
	RCP4.5	2.6	3.8	8.6	-4.9	-	-2.2	3.5	1.5	4.8	7.8	-6.3	-	4.0	1.9	4.9
	RCP8.5	3.4	4.4	13.4	-3.3	7.7	-0.6	4.1	3.2	0.5	4.4	-8.2	2.4	3.3	2.1	5.5
2080s	RCP2.6	-2.5	1.9	-	-	4.7	-	3.8	6.4	13.8	9.6	-	2.6	5.5	-	8.2
	RCP4.5	4.1	5.9	10.0	-1.7	-	3.3	5.2	8.1	0.9	2.1	-3.3	-	4.4	3.3	6.3
	RCP8.5	3.8	8.3	13.0	-3.5	4.4	-4.1	0.2	-3.9	-2.8	2.3	-9.6	-1.0	1.3	0.4	3.6
Mean annual ET – Baseline: 692.3																
2050s	RCP2.6	4.8	3.4	-	-	4.3	-	2.9	3.2	1.8	1.7	-	3.9	3.0	-	2.5
	RCP4.5	4.8	3.6	4.7	6.7	-	9.0	5.5	5.8	2.4	2.5	5.6	-	4.1	5.1	4.3
	RCP8.5	6.3	5.5	5.8	9.9	8.2	10.4	7.3	8.4	5.5	4.8	7.1	6.5	6.3	7.1	6.3
2080s	RCP2.6	3.9	2.4	-	-	3.6	-	2.4	3.3	1.3	2.2	-	3.5	2.6	-	2.1
	RCP4.5	6.2	4.7	4.8	7.6	-	9.9	7.2	7.5	4.7	4.5	6.5	-	5.8	6.4	5.6
	RPC8.5	12.9	9.9	9.6	15.6	13.4	17.8	12.4	14.3	10.8	8.9	12.5	12.1	11.6	12.5	11.7
Mean annual net precipitation – Baseline: 574.8																
2050s	RCP2.6	0.4	9.2	-	-	14.9	-	-0.9	3.8	16.2	21.6	-	0.4	8.3	-	15.5
	RCP4.5	0.0	4.1	13.3	-18.9	-	-15.8	1.1	-3.5	7.8	14.1	-20.6	-	3.9	-1.9	5.6
	RCP8.5	-0.1	3.1	22.6	-19.1	7.0	-14.0	0.2	-3.1	-5.6	4.1	-26.5	-2.6	-0.3	-3.8	4.6
2080s	RCP2.6	-10.2	1.2	-	-	6.0	-	5.4	10.2	28.8	18.5	-	1.5	9.0	-	15.5
	RCP4.5	1.6	7.4	16.3	-12.8	-	-4.7	2.8	8.8	-3.6	-0.9	-15.2	-	2.6	-0.4	7.2
	RCP8.5	-7.1	6.3	17.0	-26.6	-6.5	-30.4	-14.6	-25.9	-19.3	-5.5	-36.2	-16.9	-11.1	-14.1	-6.1
Days where Precipitation > ET: Baseline 1826 of 4748 days																
2050s	RCP2.6	-26	9	-	-	-6	-	-20	-4	21	26	-	-14	-1	-	-2
	RCP4.5	-28	-8	5	-79	-	-83	-26	-32	-3	4	-60	-	-15	-29	-29
	RCP8.5	-31	-21	-5	-113	-20	-98	-35	-45	-50	-16	-91	-38	-27	-44	-42
2080s	RCP2.6	-38	-5	-	-	0	-	-10	10	49	21	-	-5	8	-	7
	RCP4.5	-32	-13	4	-83	-	-84	-35	-24	-33	-33	-71	-	-29	-38	-38
	RCP8.5	-55	-21	-18	-189	-65	-157	-79	-107	-121	-67	-118	-82	-68	-81	-75

SM2. Projected changes in simulated stream flow

Table SM2.1 presents the projected changes in high flows (Q5, discharges exceeded for 5% of the time), mean discharge and low flows (Q95, discharges exceeded for 95% of the time) at two gauging stations for all of the climate change scenarios including the three ensemble means. These data are used to summarise the projected changes in stream flow above (Rocher) and below (Pont de Pierre) the Dauges mire as simulated by the MIKE SHE / MIKE 11 model within Table 3 of the paper.

Table SM2.1. Baseline simulated Q5, mean discharge and Q95 (m^3s^{-1}) for two gauging stations in the Dauges catchment (Rocher upstream of the mire, Pont de Pierre downstream of the mire – see Figure 1) and changes in these discharge metrics (%) simulated for each GCM / RCM pair and the three ensemble means (EM) for the three RCP scenarios in the 2050s and 2080s. Shaded cells indicate reductions compared to the baseline. See Figure 1 within the paper for locations of gauging stations.

		GCM / RCM 1	GCM / RCM 2	GCM / RCM 3	GCM / RCM 4	GCM / RCM 5	GCM / RCM 6	GCM / RCM 7	GCM / RCM 8	GCM / RCM 9	GCM / RCM 10	GCM / RCM 11	GCM / RCM 12	EM_6	EM_{10}	EM_{all}
Rocher																
Q5 – Baseline: 0.00794 m^3s^{-1}																
2050s	RCP2.6	10.4	16.1	-	-	27.7	-	1.6	5.3	22.0	28.0	-	2.6	14.2	-	14.9
	RCP4.5	6.7	8.7	21.5	0.5	-	4.0	12.9	7.2	16.1	23.1	-13.1	-	11.6	7.1	7.1
	RCP8.5	8.7	8.9	35.3	11.9	21.5	17.2	14.3	12.4	10.7	13.6	-17.6	7.9	11.9	9.5	9.9
2080s	RCP2.6	-4.0	5.1	-	-	12.3	-	12.4	16.2	33.4	24.7	-	5.1	14.2	-	14.8
	RCP4.5	16.4	16.9	21.7	8.7	-	25.3	16.7	23.8	10.8	10.7	-1.0	-	16.1	13.7	13.7
	RCP8.5	14.2	18.6	32.4	25.0	13.0	12.7	7.0	0.5	12.0	15.5	-19.4	3.0	9.7	8.2	8.3
Mean discharge – Baseline: 0.00255 m^3s^{-1}																
2050s	RCP2.6	6.8	13.2	-	-	23.3	-	1.7	6.6	20.4	26.8	-	4.1	12.3	-	12.7
	RCP4.5	5.8	7.1	19.7	-8.4	-	-3.9	8.6	3.3	12.3	20.1	-16.2	-	9.3	3.9	3.9
	RCP8.5	7.2	7.9	32.6	-1.5	16.5	3.8	8.8	7.2	2.8	9.8	-21.9	5.2	6.8	4.3	5.3
2080s	RCP2.6	-6.1	2.8	-	-	10.2	-	9.5	15.3	34.6	23.8	-	5.5	13.2	-	11.7
	RCP4.5	10.2	13.2	22.9	-1.3	-	13.0	12.4	19.5	4.0	5.8	-7.4	-	10.5	7.9	7.9
	RPC8.5	7.0	15.5	29.0	6.7	5.1	-4.4	-0.2	-9.1	1.3	7.8	-27.1	-3.9	2.4	0.6	0.3
Q95 – Baseline: 0.00028 m^3s^{-1}																
2050s	RCP2.6	-8.3	7.6	-	-	2.1	-	-1.7	5.2	11.7	13.7	-	-3.4	5.2	-	5.3
	RCP4.5	-4.2	2.7	7.0	-66.7	-	-57.8	-11.0	-15.9	-3.0	1.7	-61.8	-	-3.5	-11.9	-11.9
	RCP8.5	-7.8	3.4	9.1	-69.8	-3.1	-59.3	-12.5	-26.8	-32.7	-5.7	-68.3	-13.6	-11.3	-24.4	-17.9
2080s	RCP2.6	-33.2	1.8	-	-	4.3	-	2.5	6.7	24.1	10.4	-	-0.7	6.3	-	5.2
	RCP4.5	-9.9	3.0	8.6	-60.6	-	-45.4	-10.9	-2.0	-24.7	-20.8	-60.8	-	-7.9	-13.8	-13.8
	RCP8.5	-47.2	5.0	4.9	-75.8	-21.0	-73.6	-55.7	-67.3	-63.4	-41.9	-77.4	-62.1	-53.4	-58.6	-57.6
Point de Pierre																
Q5 – Baseline: 0.0938 m^3s^{-1}																
2050s	RCP2.6	6.5	9.7	-	-	18.2	-	0.5	3.3	15.0	16.7	-	2.4	9.3	-	9.4
	RCP4.5	4.5	5.0	12.9	-1.9	-	1.5	7.4	4.5	10.7	14.0	-9.8	-	8.3	4.3	4.3
	RCP8.5	5.3	5.0	22.0	6.2	12.2	8.9	8.3	6.0	5.9	7.6	-12.7	6.4	6.4	6.6	7.5
2080s	RCP2.6	-3.1	1.4	-	-	7.5	-	6.2	10.6	21.5	14.3	-	3.1	9.5	-	8.2
	RCP4.5	9.6	10.5	15.1	3.6	-	14.7	10.6	16.2	5.7	5.3	-1.1	-	10.2	9.3	9.3
	RCP8.5	7.3	11.4	21.3	16.3	8.5	5.1	2.8	-2.2	8.8	9.1	-15.6	1.3	5.8	5.4	4.9
Mean discharge – Baseline: 0.0292 m^3s^{-1}																
2050s	RCP2.6	4.5	8.7	-	-	16.0	-	0.6	3.9	13.6	18.3	-	2.6	8.0	-	8.1
	RCP4.5	3.5	4.6	13.1	-6.9	-	-4.0	5.4	1.8	8.0	13.6	-12.1	-	5.9	2.1	2.1
	RCP8.5	4.2	4.7	21.9	-2.7	10.7	0.6	5.1	4.1	1.1	6.2	-15.9	2.9	4.0	2.0	3.0
2080s	RCP2.6	-4.9	1.5	-	-	6.5	-	6.0	9.8	23.5	16.1	-	3.3	8.6	-	7.5
	RCP4.5	6.6	8.6	15.3	-2.2	-	7.7	7.7	12.5	2.1	3.6	-5.9	-	6.6	4.8	4.8
	RCP8.5	3.6	9.4	19.0	1.6	2.4	-5.7	-1.5	-8.4	-1.2	4.4	-20.1	-4.0	0.1	-1.3	-1.2
Q95 – Baseline: 0.0031 m^3s^{-1}																
2050s	RCP2.6	-12.2	6.7	-	-	1.0	-	-2.4	7.7	11.8	14.6	-	-7.1	5.3	-	3.4
	RCP4.5	-10.8	2.2	1.1	-35.0	-	-26.1	-13.6	-13.2	1.9	3.0	-30.9	-	-5.5	-12.5	-12.5
	RCP8.5	-11.0	2.6	4.1	-39.2	-5.2	-30.1	-12.2	-17.2	-15.4	-4.7	-32.4	-14.0	-10.8	-16.6	-15.4
2080s	RCP2.6	-18.7	2.9	-	-	2.1	-	1.5	5.7	23.2	10.7	-	-3.8	4.9	-	3.4
	RCP4.5	-11.9	3.6	4.6	-29.9	-	-26.0	-12.4	-5.4	-14.1	-11.7	-27.0	-	-9.4	-13.9	-13.9
	RCP8.5	-28.3	-0.3	-3.1	-50.7	-16.2	-45.5	-30.6	-37.9	-34.5	-21.2	-44.2	-31.8	-27.1	-31.0	-29.9

SM3. Projected changes in groundwater depths

Table SM3.1 presents the projected changes in high groundwater depths (GWD5, water table depths exceeded for 5% of the time), mean groundwater depth and low groundwater depths (GWD95, water table depths exceeded for 95% of the time) at four wells within the Dauges mire for all of the climate change scenarios including the three ensemble means. These data are used to summarise the projected changes in groundwater depths at these wells as simulated by the MIKE SHE / MIKE 11 model within Table 4 of the paper. Table SM3.2 presents the same groundwater depth metrics averaged across the MIKE SHE grid cells that are within the area defined as mire that are used to summarise mire-wide groundwater depth changes within Table 5 of the paper.

Table SM3.1 Baseline simulated GWD-5, mean groundwater depth (GWD) and GWD-95 (cm above ground) at the locations of four dip wells within the Dauges mire and changes in these groundwater level metrics (cm) simulated for each GCM / RCM pair and the three ensemble means (EM) for the three RCP scenarios in the 2050s and 2080s. Shaded cells indicate reductions compared to the baseline. See Figure 1 within the paper for locations of dip wells.

		GCM / RCM 1	GCM / RCM 2	GCM / RCM 3	GCM / RCM 4	GCM / RCM 5	GCM / RCM 6	GCM / RCM 7	GCM / RCM 8	GCM / RCM 9	GCM / RCM 10	GCM / RCM 11	GCM / RCM 12	EM_6	EM_{10}	EM_{all}
Well D3																
GWD-5 – Baseline: 2.1 cm																
2050s	RCP2.6	0.1	0.1	-	-	0.2	-	0.0	0.1	0.2	0.2	-	0.0	0.1	-	0.1
	RCP4.5	0.1	0.1	0.2	-0.1	-	-0.1	0.1	0.1	0.1	0.2	-0.2	-	0.1	0.0	0.0
	RCP8.5	0.1	0.1	0.3	0.0	0.1	0.1	0.1	0.1	0.1	0.1	-0.2	0.1	0.1	0.0	0.1
2080s	RCP2.6	-0.1	0.0	-	-	0.0	-	0.1	0.1	0.3	0.2	-	0.0	0.1	-	0.1
	RCP4.5	0.1	0.1	0.3	0.0	-	0.2	0.1	0.2	0.1	0.1	-0.1	-	0.1	0.1	0.1
	RCP8.5	0.1	0.1	0.3	0.1	0.1	0.0	0.0	-0.1	0.0	0.1	-0.3	0.0	0.0	0.0	0.0
Mean GWD – Baseline: -3.4 cm																
2050s	RCP2.6	-1.4	0.7	-	-	0.1	-	-0.2	0.5	1.2	1.3	-	-0.7	0.5	-	0.4
	RCP4.5	-1.3	0.1	0.5	-6.5	-	-5.5	-1.6	-2.1	0.1	0.4	-4.6	-	-0.6	-1.6	-1.6
	RCP8.5	-1.4	0.2	0.8	-8.2	-0.6	-6.2	-1.4	-2.8	-3.0	-0.6	-6.2	-2.0	-1.4	-2.5	-2.3
2080s	RCP2.6	-2.6	0.1	-	-	0.2	-	0.2	0.5	1.9	1.1	-	-0.2	0.6	-	0.4
	RCP4.5	-1.7	0.3	0.9	-5.6	-	-4.7	-1.5	-0.7	-2.5	-1.8	-4.8	-	-1.2	-1.9	-1.9
	RPC8.5	-3.9	0.0	0.2	-11.9	-2.6	-10.6	-5.3	-7.9	-7.5	-3.8	-8.9	-5.8	-4.4	-5.3	-5.0
GWD-95 – Baseline: -31.4 cm																
2050s	RCP2.6	-7.6	5.5	-	-	2.0	-	-1.3	4.5	10.9	12.4	-	-3.2	4.7	-	3.3
	RCP4.5	-4.7	1.8	3.7	-27.9	-	-26.7	-6.7	-12.7	4.2	5.3	-23.9	-	-1.9	-7.5	-7.5
	RCP8.5	-7.7	1.6	7.2	-28.5	-2.7	-27.3	-5.7	-14.4	-19.7	-3.4	-26.9	-10.3	-6.3	-13.3	-12.3
2080s	RCP2.6	-15.0	1.0	-	-	2.8	-	2.4	5.7	16.6	10.4	-	0.0	5.8	-	4.2
	RCP4.5	-9.4	4.2	7.1	-27.0	-	-25.4	-7.4	-1.5	-15.9	-10.0	-24.0	-	-5.3	-9.6	-9.6
	RCP8.5	-21.5	2.1	3.6	-35.3	-12.4	-29.7	-25.5	-27.7	-27.8	-20.6	-29.3	-26.8	-23.2	-25.4	-25.0
Well D7																
GWD-5 – Baseline: 2.7 cm																
2050s	RCP2.6	0.2	0.2	-	-	0.3	-	0.1	0.1	0.2	0.2	-	0.1	0.1	-	0.2
	RCP4.5	0.1	0.1	0.2	0.0	-	0.0	0.2	0.1	0.2	0.2	-0.2	-	0.1	0.1	0.1
	RCP8.5	0.1	0.1	0.4	0.1	0.2	0.2	0.1	0.1	0.1	0.1	-0.2	0.1	0.1	0.1	0.2
2080s	RCP2.6	-0.1	0.0	-	-	0.1	-	0.1	0.1	0.3	0.2	-	0.1	0.1	-	0.1
	RCP4.5	0.2	0.2	0.3	0.0	-	0.3	0.2	0.3	0.1	0.1	0.0	-	0.1	0.2	0.2
	RCP8.5	0.1	0.1	0.4	0.2	0.1	0.1	0.1	-0.1	0.1	0.2	-0.2	0.0	0.1	0.1	0.1
Mean GWD – Baseline: -3.5 cm																
2050s	RCP2.6	-1.7	0.1	-	-	-0.8	-	-0.5	0.0	0.5	0.4	-	-0.6	-0.1	-	-0.2
	RCP4.5	-1.7	-0.1	-0.1	-4.3	-	-4.6	-1.8	-1.9	-0.5	-0.4	-2.9	-	-1.0	-1.7	-1.7
	RCP8.5	-1.7	-0.3	-0.3	-5.8	-1.6	-5.5	-1.9	-2.9	-2.4	-0.9	-3.3	-2.2	-1.6	-2.4	-2.2
2080s	RCP2.6	-2.2	0.0	-	-	-0.4	-	-0.5	-0.2	0.8	0.1	-	-0.7	-0.2	-	-0.2
	RCP4.5	-2.3	-0.5	0.3	-4.1	-	-5.0	-2.1	-1.8	-2.3	-1.9	-3.5	-	-1.7	-2.1	-2.1
	RCP8.5	-3.9	-0.9	-0.6	-9.2	-2.8	-8.5	-4.6	-5.7	-6.6	-3.7	-4.6	-4.4	-4.2	-4.6	-4.5
GWD-95 – Baseline: -33.7 cm																
2050s	RCP2.6	-8.9	0.0	-	-	-4.2	-	-2.2	0.1	2.1	1.8	-	-2.4	-1.2	-	-1.8
	RCP4.5	-7.9	-0.9	-0.2	-18.2	-	-19.7	-9.0	-9.2	-3.9	-4.0	-12.8	-	-5.6	-8.9	-8.9
	RCP8.5	-8.3	-1.9	-1.5	-21.4	-8.2	-20.9	-9.7	-13.5	-12.6	-5.6	-14.3	-10.1	-8.3	-11.5	-11.2
2080s	RCP2.6	-10.6	-0.2	-	-	-1.9	-	-2.8	-1.4	3.2	-1.4	-	-4.1	-2.0	-	-2.1
	RCP4.5	-11.0	-3.4	2.0	-17.2	-	-21.1	-10.4	-9.1	-12.1	-10.5	-15.1	-	-9.0	-10.8	-10.8
	RCP8.5	-17.4	-4.7	-2.3	-22.1	-13.5	-22.0	-19.5	-21.5	-21.8	-18.5	-17.9	-18.1	-18.4	-19.7	-18.8

Table SM3.1 cont.

		GCM / RCM 1	GCM / RCM 2	GCM / RCM 3	GCM / RCM 4	GCM / RCM 5	GCM / RCM 6	GCM / RCM 7	GCM / RCM 8	GCM / RCM 9	GCM / RCM 10	GCM / RCM 11	GCM / RCM 12	EM_6	EM_{10}	EM_{all}
Well D13																
GWD-5 – Baseline: 2.1 cm																
2050s	RCP2.6	0.1	0.1	-	-	0.3	-	0.0	0.1	0.2	0.2	-	0.0	0.1	-	0.1
	RCP4.5	0.1	0.1	0.2	-0.1	-	-0.0	0.2	0.1	0.1	0.2	-0.2	-	0.1	0.1	0.1
	RCP8.5	0.1	0.1	0.3	0.0	0.2	0.1	0.1	0.1	0.1	0.1	-0.2	0.1	0.1	0.0	0.1
2080s	RCP2.6	-0.1	0.0	-	-	0.0	-	0.1	0.1	0.3	0.2	-	0.0	0.1	-	0.1
	RCP4.5	0.1	0.1	0.3	-0.0	-	0.2	0.2	0.2	0.1	0.1	-0.0	-	0.1	0.1	0.1
	RCP8.5	0.1	0.1	0.3	0.1	0.1	0.0	0.0	-0.1	0.0	0.2	-0.2	-0.0	0.1	0.0	0.0
Mean GWD – Baseline: -2.2 cm																
2050s	RCP2.6	-1.3	0.3	-	-	-0.1	-	-0.3	0.4	0.7	0.8	-	-0.8	0.3	-	0.1
	RCP4.5	-1.3	0.0	-0.1	-4.5	-	-3.3	-1.2	-1.2	0.2	0.1	-3.3	-	-0.5	-1.2	-1.2
	RCP8.5	-1.2	0.0	-0.0	-5.7	-0.7	-4.3	-1.2	-1.9	-1.4	-0.5	-3.5	-1.5	-1.0	-1.7	-1.6
2080s	RCP2.6	-2.0	0.2	-	-	-0.0	-	0.0	0.3	1.1	0.5	-	-0.5	0.2	-	0.1
	RCP4.5	-1.2	0.1	0.1	-3.7	-	-3.6	-1.3	-0.7	-1.2	-1.1	-3.0	-	-0.8	-1.3	-1.3
	RCP8.5	-3.6	-0.4	-0.6	-9.7	-2.1	-7.7	-4.0	-5.6	-5.3	-2.5	-5.5	-4.0	-3.2	-4.0	-3.8
GWD-95 – Baseline: -22.1 cm																
2050s	RCP2.6	-9.3	3.1	-	-	-0.7	-	-1.9	5.4	7.7	9.1	-	-7.1	2.8	-	1.1
	RCP4.5	-8.8	-0.3	-2.1	-28.5	-	-22.7	-9.8	-9.2	2.1	0.9	-20.8	-	-3.7	-9.1	-9.1
	RCP8.5	-8.5	1.4	-1.0	-32.2	-5.2	-27.9	-9.2	-13.4	-11.5	-4.0	-22.0	-10.5	-7.4	-12.4	-11.8
2080s	RCP2.6	-13.9	1.9	-	-	-1.0	-	0.5	4.3	12.2	6.0	-	-3.8	2.8	-	0.8
	RCP4.5	-8.7	2.2	-1.1	-23.5	-	-25.9	-9.5	-5.0	-8.6	-8.7	-20.2	-	-5.6	-9.7	-9.7
	RCP8.5	-25.1	-2.3	-6.0	-36.7	-15.4	-35.6	-26.4	-31.8	-31.8	-20.4	-29.8	-25.3	-23.2	-26.3	-25.7
Well D18																
GWD-5 – Baseline: 2.2cm																
2050s	RCP2.6	0.1	0.1	-	-	0.2	-	0.0	0.0	0.2	0.2	-	0.0	0.1	-	0.1
	RCP4.5	0.0	0.1	0.1	-0.1	-	-0.1	0.1	0.0	0.1	0.1	-0.1	-	0.1	0.0	0.0
	RCP8.5	0.0	0.0	0.2	0.0	0.1	0.0	0.0	0.0	0.0	0.1	-0.2	0.0	0.0	0.0	0.0
2080s	RCP2.6	-0.1	0.0	-	-	0.0	-	0.0	0.1	0.2	0.2	-	0.0	0.1	-	0.1
	RCP4.5	0.1	0.1	0.2	0.0	-	0.1	0.1	0.1	0.0	0.0	-0.1	-	0.1	0.0	0.0
	RCP8.5	0.0	0.1	0.2	0.0	0.0	0.0	0.0	-0.1	0.1	0.1	-0.2	-0.1	0.0	0.0	0.0
Mean GWD – Baseline: -5.2 cm																
2050s	RCP2.6	-1.8	0.0	-	-	-1.3	-	-0.5	0.0	0.5	0.5	-	-0.5	-0.2	-	-0.3
	RCP4.5	-1.7	0.0	0.0	-4.3	-	-4.9	-2.1	-2.2	-0.6	-0.5	-2.4	-	-1.1	-1.7	-1.7
	RCP8.5	-1.8	-0.3	-0.4	-6.4	-1.7	-6.6	-2.0	-3.1	-2.9	-0.9	-2.9	-2.4	-1.8	-2.5	-2.5
2080s	RCP2.6	-2.1	0.0	-	-	-0.4	-	-0.7	-0.3	0.8	0.2	-	-0.7	-0.3	-	-0.3
	RCP4.5	-2.6	-0.7	0.5	-4.5	-	-5.6	-2.4	-2.2	-2.7	-1.9	-3.5	-	-2.0	-2.4	-2.4
	RPC8.5	-3.7	-0.8	-0.5	-10.6	-2.5	-9.2	-4.6	-5.9	-7.8	-4.0	-4.1	-4.4	-4.2	-4.6	-4.4
GWD-95 – Baseline: -37.9 cm																
2050s	RCP2.6	-8.0	0.2	-	-	-5.3	-	-2.0	0.6	2.0	1.7	-	-1.6	-1.2	-	-1.8
	RCP4.5	-7.8	0.7	0.2	-16.2	-	-17.4	-8.9	-9.0	-4.0	-3.2	-11.3	-	-5.1	-7.6	-7.6
	RCP8.5	-7.4	-1.4	-1.4	-18.2	-8.0	-18.1	-9.3	-12.7	-12.1	-5.1	-12.3	-10.1	-8.0	-11.0	-10.5
2080s	RCP2.6	-9.9	0.8	-	-	-1.7	-	-4.0	-1.5	2.5	-0.7	-	-3.5	-1.9	-	-1.8
	RCP4.5	-10.9	-3.4	3.0	-15.7	-	-17.9	-10.2	-10.1	-11.8	-9.3	-14.1	-	-9.3	-10.2	-10.2
	RCP8.5	-15.6	-3.5	-1.0	-19.0	-11.4	-18.8	-17.4	-18.1	-18.4	-16.5	-15.6	-16.2	-16.5	-17.3	-16.7

Table SM3.2. Mean baseline simulated GWD-5, mean GWD and GWD-95 (cm above ground) across all MIKE SHE grid cells that are within the area defined as mire using botanical (Durepaire and Guerbaa, 2008) and pedological (Duranel, 2015) criteria and mean changes in these groundwater depth metrics (cm) simulated for each GCM / RCM pair and the three ensemble means (EM) for the three RCP scenarios in the 2050s and 2080s. Shaded cells indicate reductions compared to the baseline.

		GCM / RCM 1	GCM / RCM 2	GCM / RCM 3	GCM / RCM 4	GCM / RCM 5	GCM / RCM 6	GCM / RCM 7	GCM / RCM 8	GCM / RCM 9	GCM / RCM 10	GCM / RCM 11	GCM / RCM 12	EM_6	EM_{10}	EM_{all}
GWD-5 – Baseline: 3.3 cm																
2050s	RCP2.6	0.2	0.2	-	-	0.4	-	0.0	0.1	0.3	0.3	-	0.1	0.2	-	0.2
	RCP4.5	0.1	0.1	0.3	0.0	-	0.0	0.2	0.1	0.3	0.3	-0.2	-	0.2	0.1	0.1
	RCP8.5	0.1	0.1	0.5	0.2	0.3	0.2	0.2	0.1	0.2	0.2	-0.2	0.1	0.2	0.1	0.2
2080s	RCP2.6	-0.1	0.0	-	-	0.1	-	0.2	0.2	0.4	0.3	-	0.1	0.2	-	0.1
	RCP4.5	0.2	0.2	0.4	0.0	-	0.4	0.3	0.3	0.2	0.1	0.0	-	0.2	0.2	0.2
	RCP8.5	0.2	0.2	0.5	0.3	0.2	0.1	0.1	-0.1	0.2	0.2	-0.3	0.0	0.2	0.1	0.1
Mean GWD – Baseline: -5.8 cm																
2050s	RCP2.6	-1.3	0.4	-	-	-0.3	-	-0.3	0.3	0.9	1.1	-	-0.5	0.3	-	0.1
	RCP4.5	-1.0	0.2	0.4	-4.5	-	-4.1	-1.4	-1.7	-0.3	0.0	-2.8	-	-0.6	-1.3	-1.3
	RCP8.5	-1.1	0.1	0.4	-6.0	-0.7	-4.9	-1.4	-2.2	-2.4	-0.5	-3.6	-1.7	-1.2	-1.9	-1.8
2080s	RCP2.6	-1.8	0.1	-	-	0.0	-	-0.1	0.2	1.7	0.8	-	-0.3	0.3	-	0.2
	RCP4.5	-1.5	-0.1	0.8	-4.2	-	-4.1	-1.4	-0.9	-2.1	-1.4	-3.3	-	-1.2	-1.6	-1.6
	RPC8.5	-2.8	0.0	0.2	-9.2	-1.9	-7.6	-3.7	-5.1	-5.7	-2.8	-5.1	-4.1	-3.2	-3.8	-3.7
GWD-95 – Baseline: -34.1 cm																
2050s	RCP2.6	-5.4	1.2	-	-	-1.9	-	-1.0	1.5	3.0	3.1	-	-1.8	0.6	-	0.1
	RCP4.5	-4.4	0.4	0.6	-14.8	-	-13.1	-5.8	-6.1	-1.7	-0.9	-9.9	-	-3.0	-5.3	-5.3
	RCP8.5	-4.8	0.1	0.5	-18.1	-3.7	-15.0	-6.0	-8.3	-8.0	-2.6	-12.0	-6.4	-5.0	-7.4	-7.1
2080s	RCP2.6	-6.9	0.4	-	-	-0.2	-	-0.8	0.5	5.5	1.8	-	-1.5	0.4	-	0.1
	RCP4.5	-6.1	-0.9	2.4	-13.2	-	-14.0	-6.0	-4.5	-7.5	-6.2	-11.4	-	-5.1	-6.6	-6.6
	RCP8.5	-11.4	-0.9	-0.5	-25.3	-7.6	-21.9	-13.3	-16.3	-17.3	-10.7	-16.4	-13.4	-11.8	-13.6	-13.1

SM4. Projected changes in indices of groundwater depth within the mire

The following figures, which are referenced in the section of the paper which evaluates the impacts of climate change upon groundwater depth (GWD), illustrate baseline and scenario changes in GWD-5 (the water table depths exceeded for 5% of the time and indicative of high [winter] groundwater; Figures SM4.1–SM4.3), mean GWD (Figures SM4.4–SM4.6) and GWD-95 (the water table depths exceeded for 95% of the time and indicative of low [summer] groundwater; Figures SM4.7–SM4.9) across the mire area defined using botanical (Durepaire and Guerbaa, 2008)[1] and pedological (Duranel, 2015)[2] criteria. Individual maps are provided for each of the GCM / RCM pairs that provide data for the three RCPs in the 2050s and 2080s. The equivalent changes are also shown for the available ensemble means. The scales of the colour bars for changes in an individual metric (i.e. GWD-5, mean GWD and GWD-95) are the same in each of the three figures to facilitate comparisons between the results for different RCPs. Bar graphs summarise the mean changes in the three water table depth metrics across all MIKE SHE grid cells within the mire for the pairs and ensemble means that are available for a given RCP. Different shading is used to differentiate the six pairs that provide data for all RCPs, those pairs that do not provide data for all RCPs as well as the ensemble means. Vertical dashed lines within the bar graphs indicate when a particular pair does not provide data for the current RCP. Note that the y-axis ranges for the bar graphs showing results for an individual metric (i.e. GWD-5, mean GWD and GWD-95) are the same in each of the three figures that show results for the different RCPs but that they vary between metrics.

[1] Durepaire P, Guerbaa K (2008) Tourbière des Dauges – Plan de gestion 2008–2012, Conservatoire Régional des Espaces Naturels du Limousin, St-Gence, France

[2] Duranel AJ (2015) Hydrology and Hydrological Modelling of Acidic Mires in Central France. PhD Thesis, University College London, London, UK

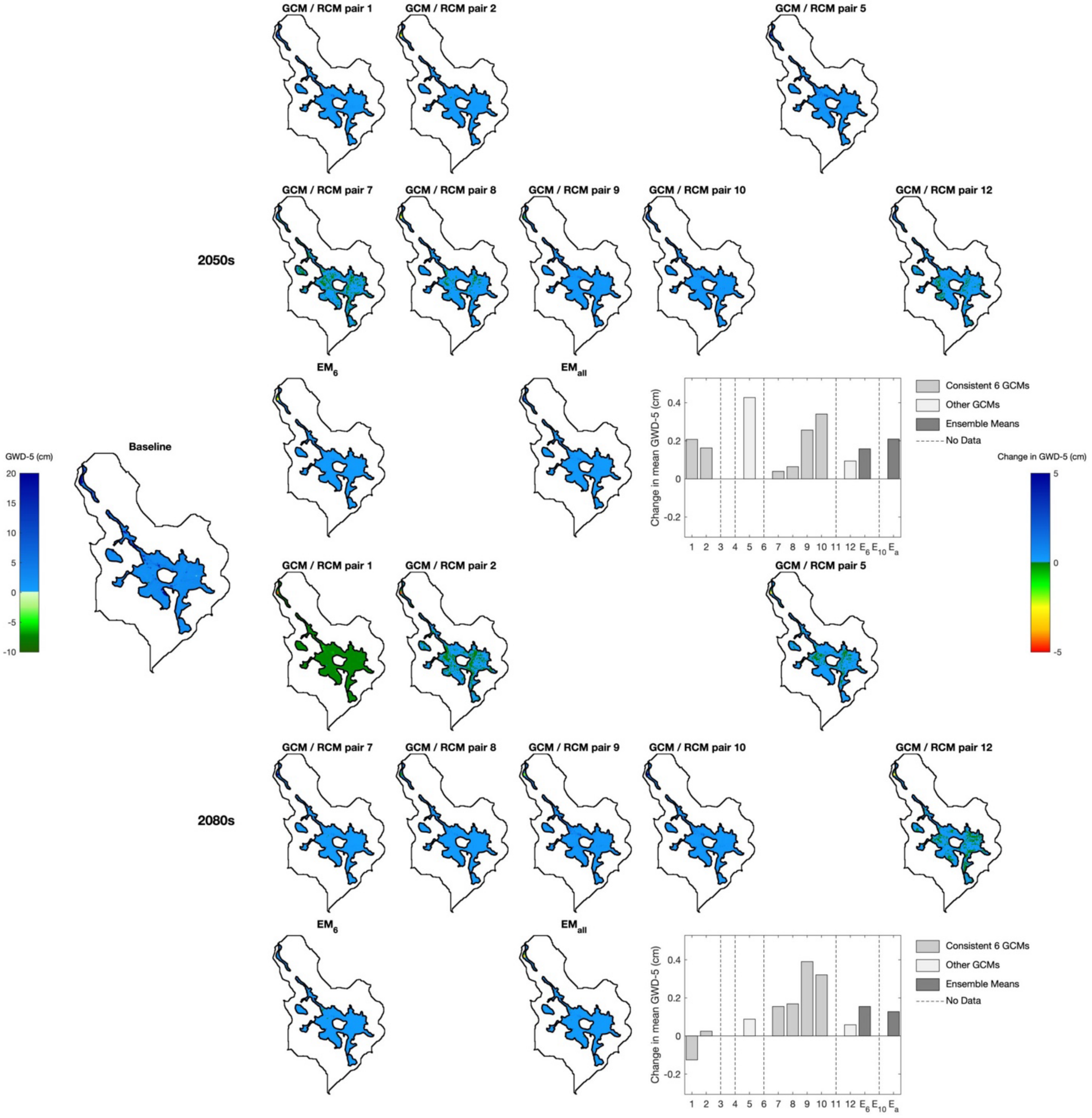

Figure SM4.1. Baseline GWD-5 and changes in GWD-5 across the mire area for RCP2.6 in the 2050s (top) and 2080s (bottom).

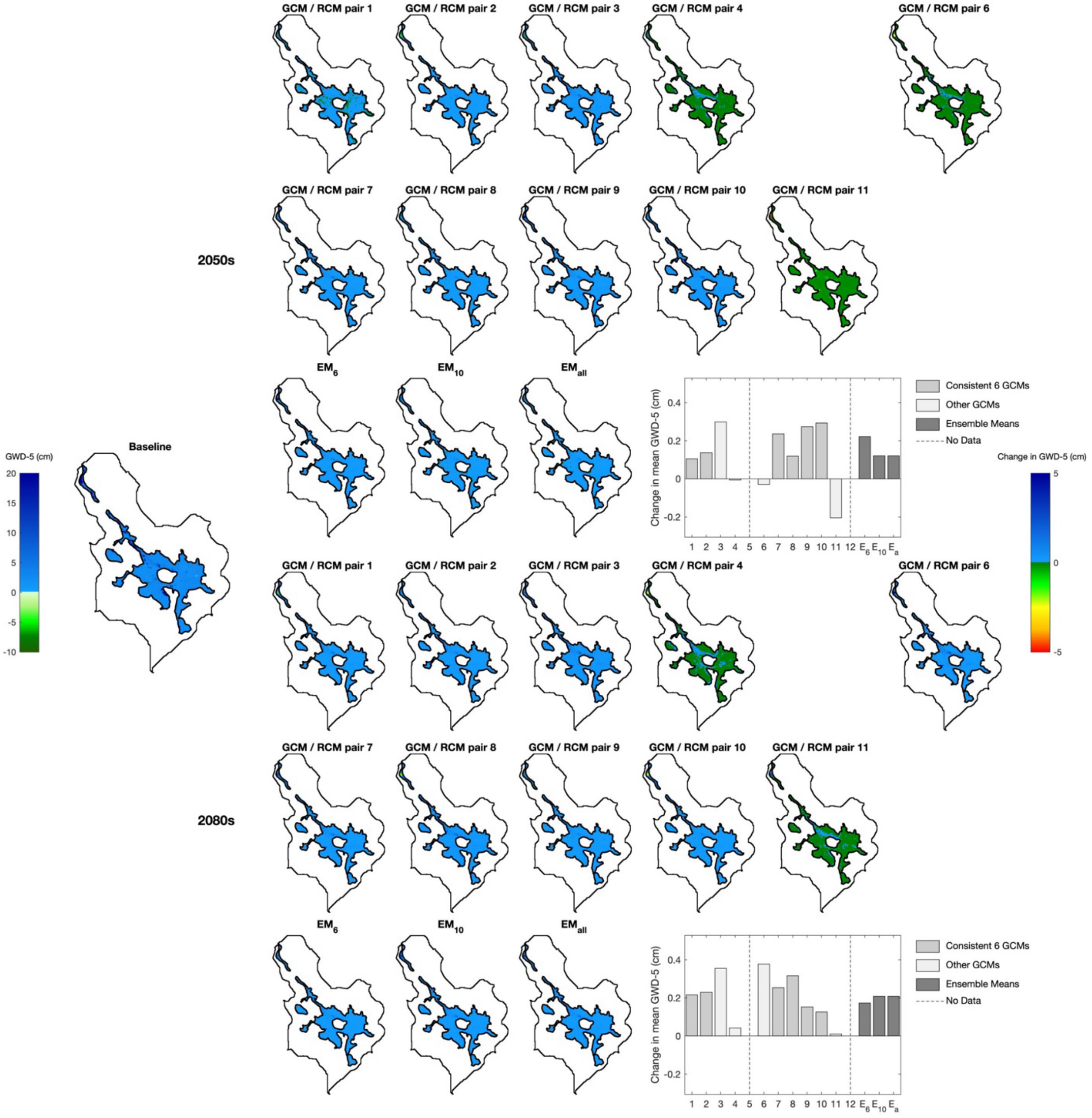

Figure SM4.2. Baseline GWD-5 and changes in GWD-5 across the mire area for RCP4.5 in the 2050s (top) and 2080s (bottom).

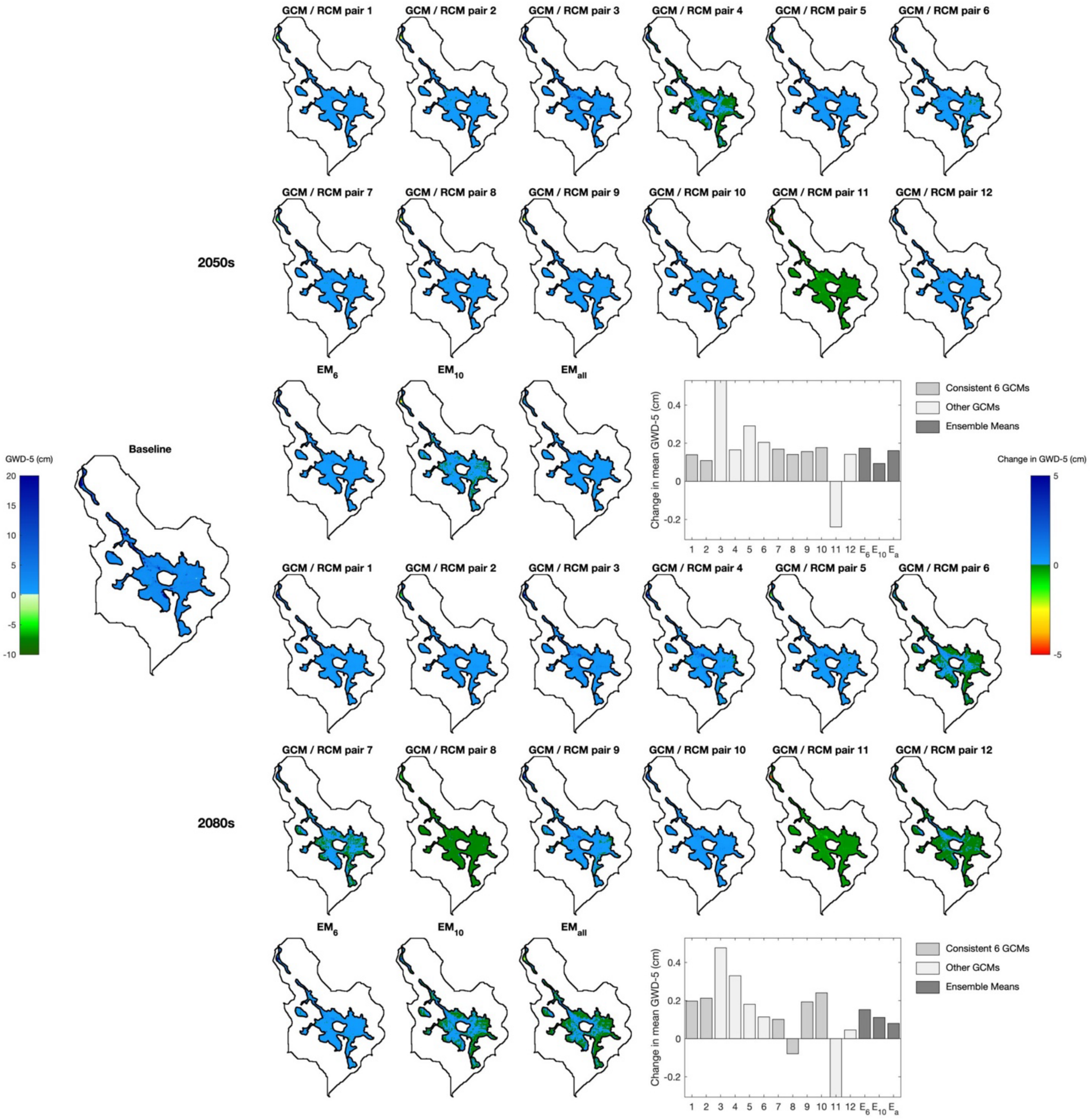

Figure SM4.3. Baseline GWD-5 and changes in GWD-5 across the mire area for RCP8.5 in the 2050s (top) and 2080s (bottom).

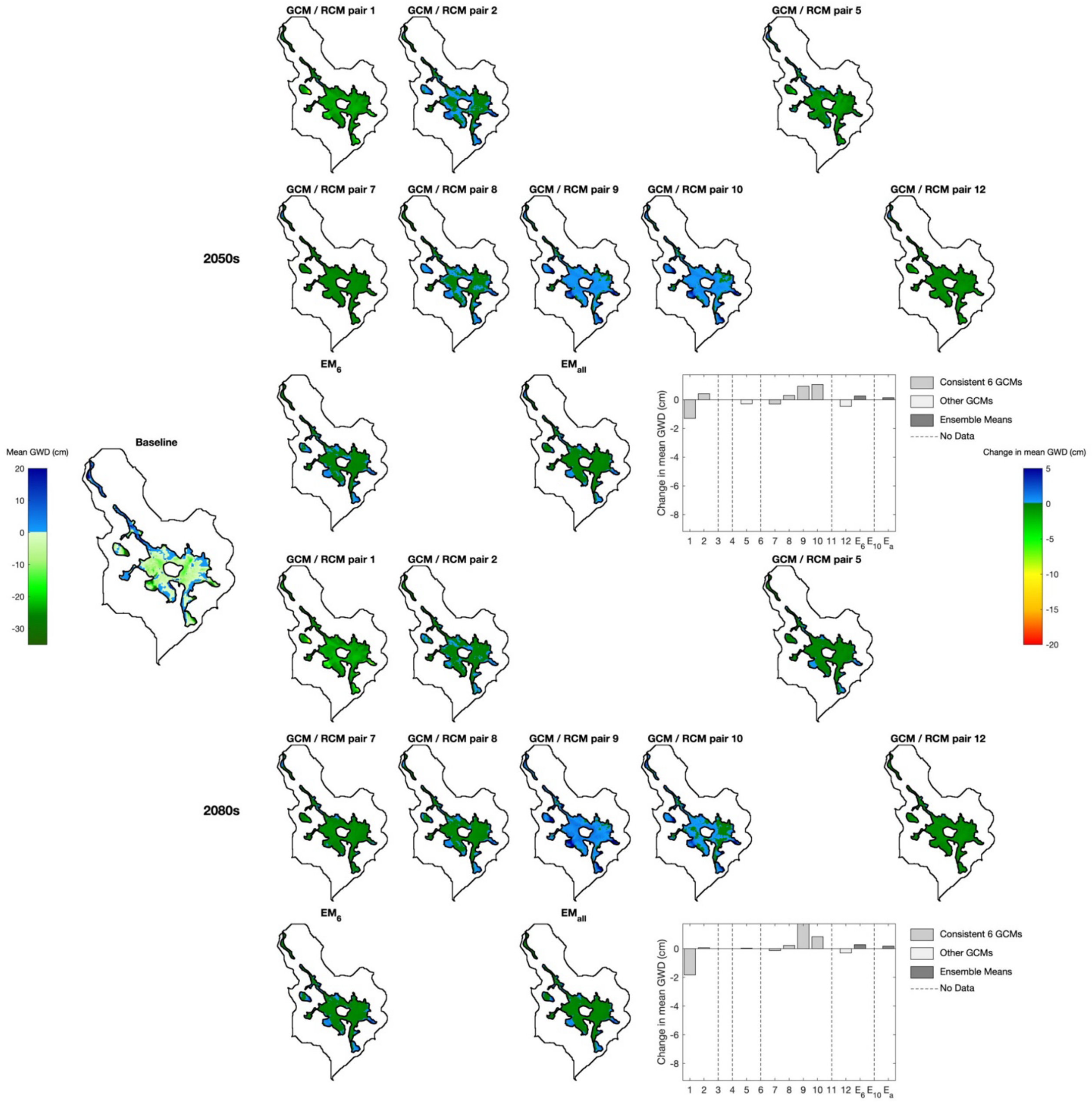

Figure SM4.4. Baseline mean GWD and changes in mean GWD across the mire area for RCP2.6 in the 2050s (top) and 2080s (bottom).

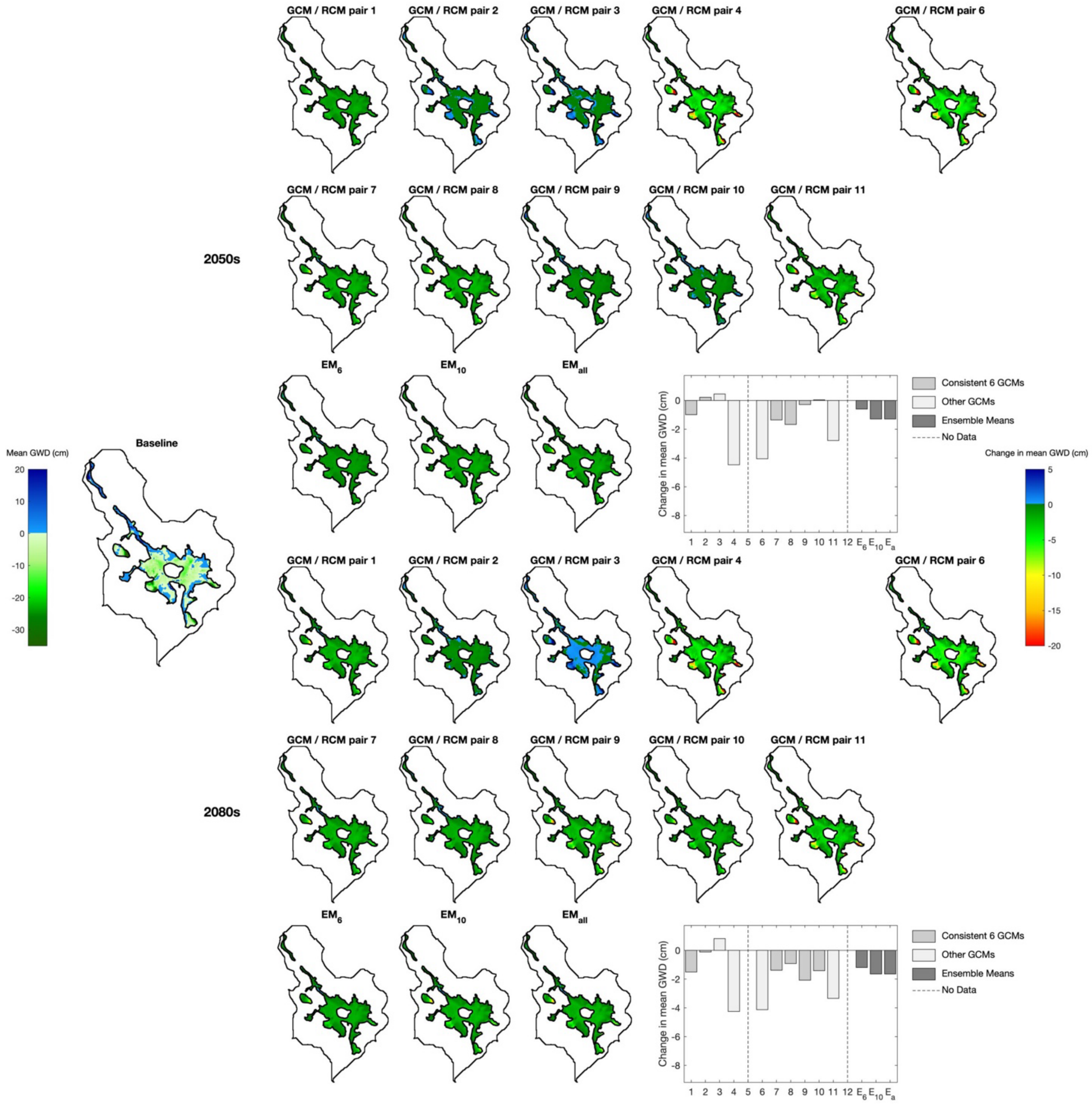

Figure SM4.5. Baseline mean GWD and changes in mean GWD across the mire area for RCP4.5 in the 2050s (top) and 2080s (bottom).

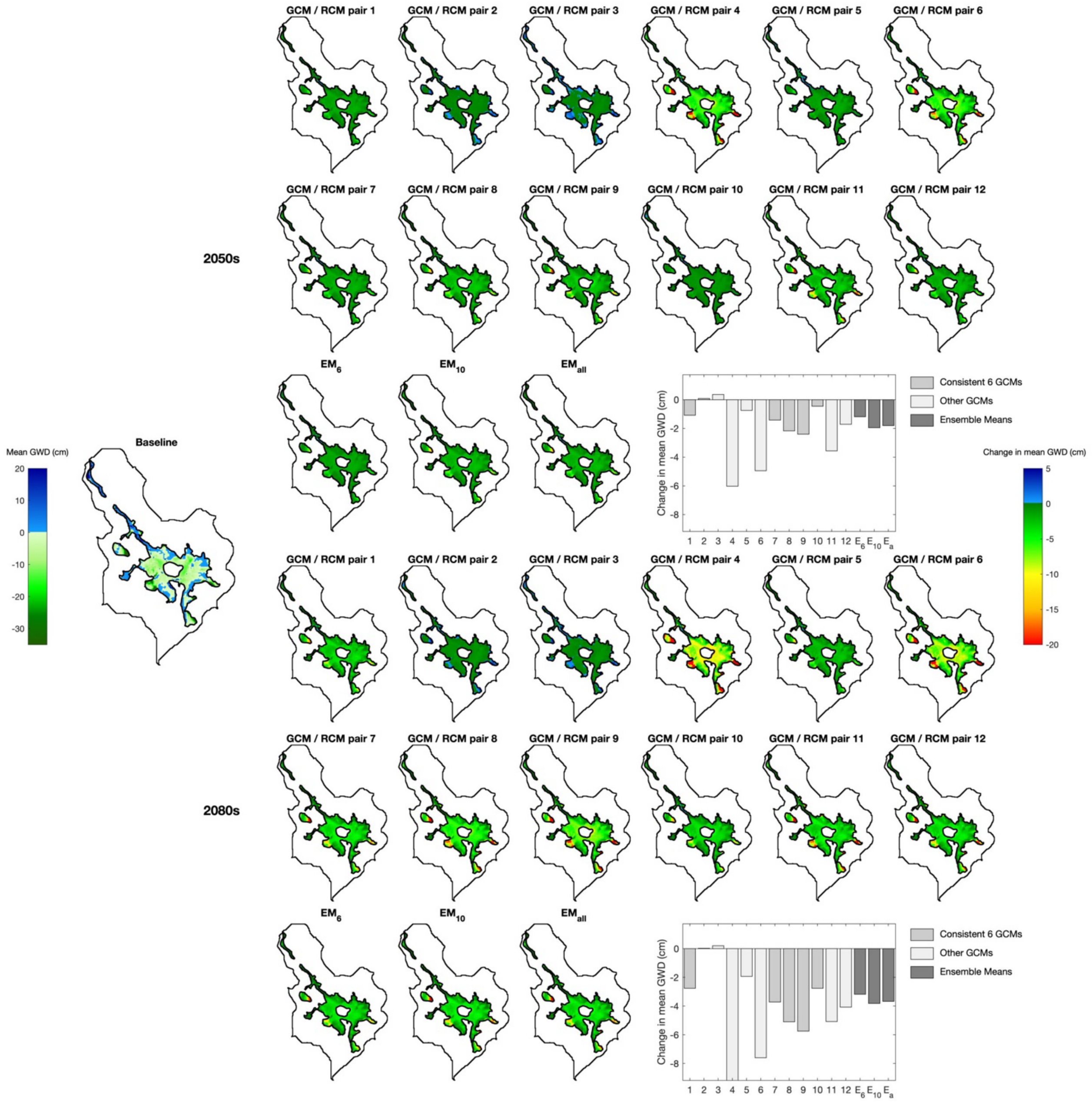

Figure SM4.6. Baseline mean GWD and changes in mean GWD across the mire area for RCP8.5 in the 2050s (top) and 2080s (bottom).

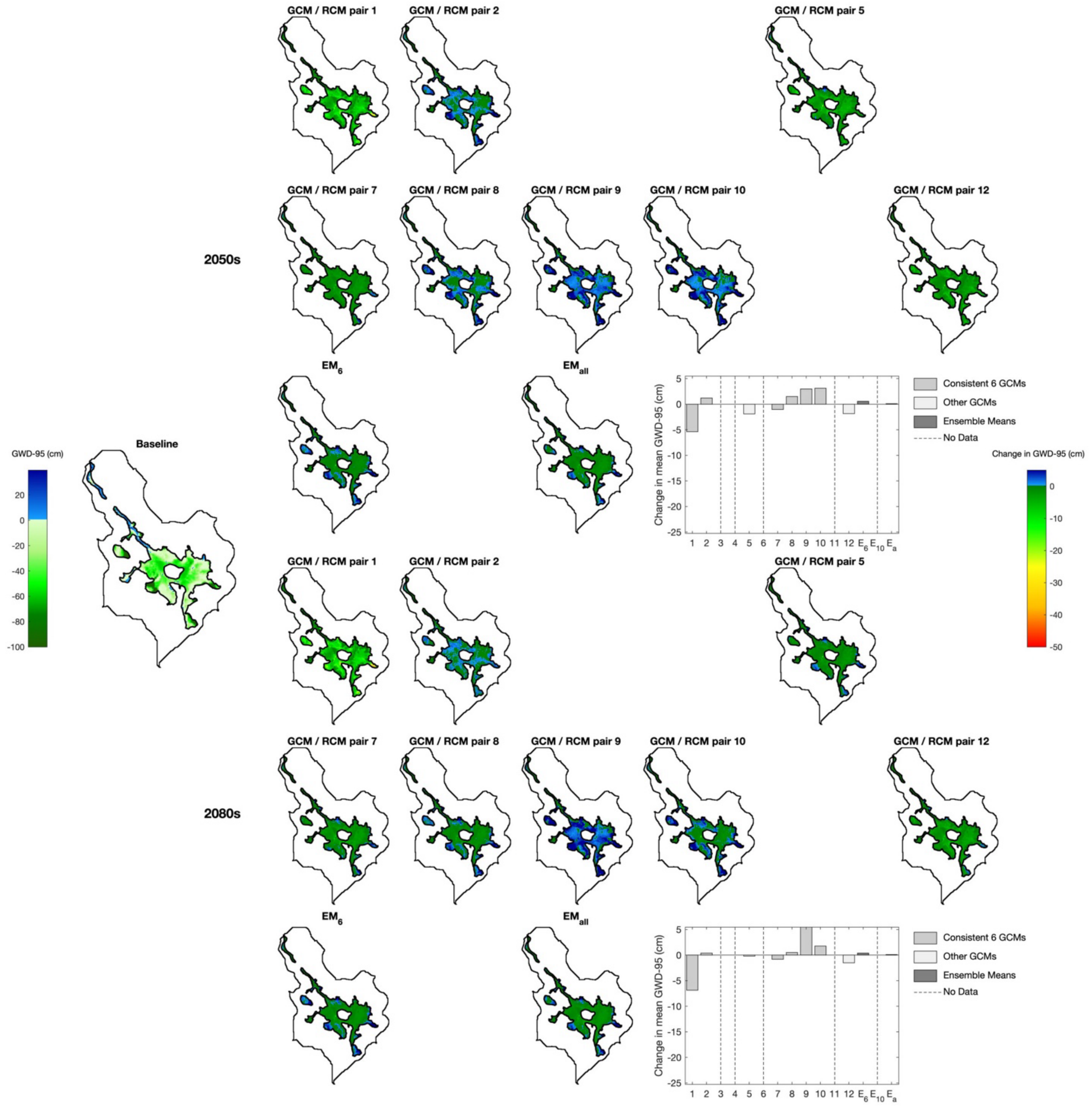

Figure SM4.7. Baseline GWD-95 and changes in GWD-95 across the mire area for RCP2.6 in the 2050s (top) and 2080s (bottom).

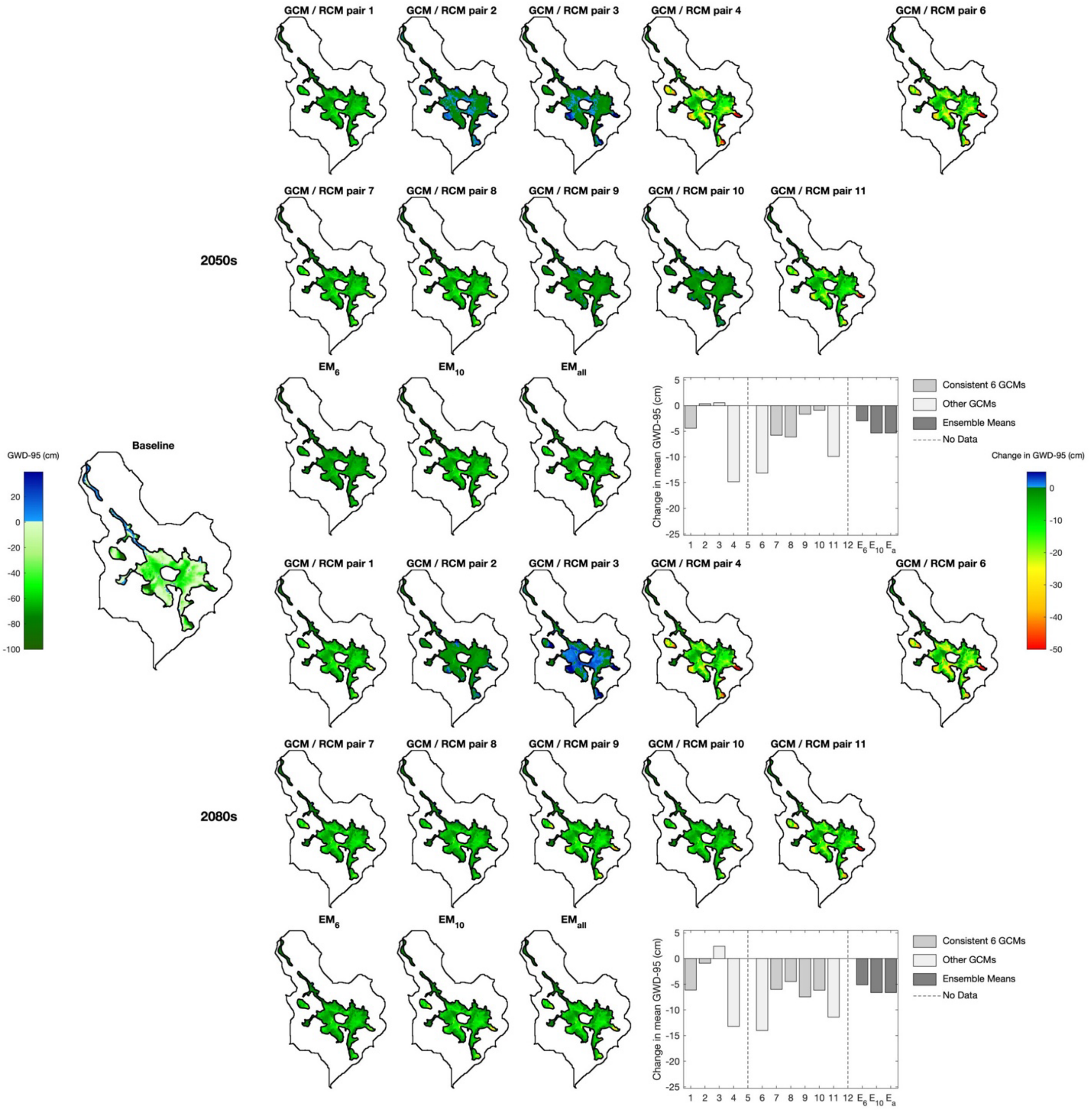

Figure SM4.8. Baseline GWD-95 and changes in GWD-95 across the mire area for RCP4.5 in the 2050s (top) and 2080s (bottom).

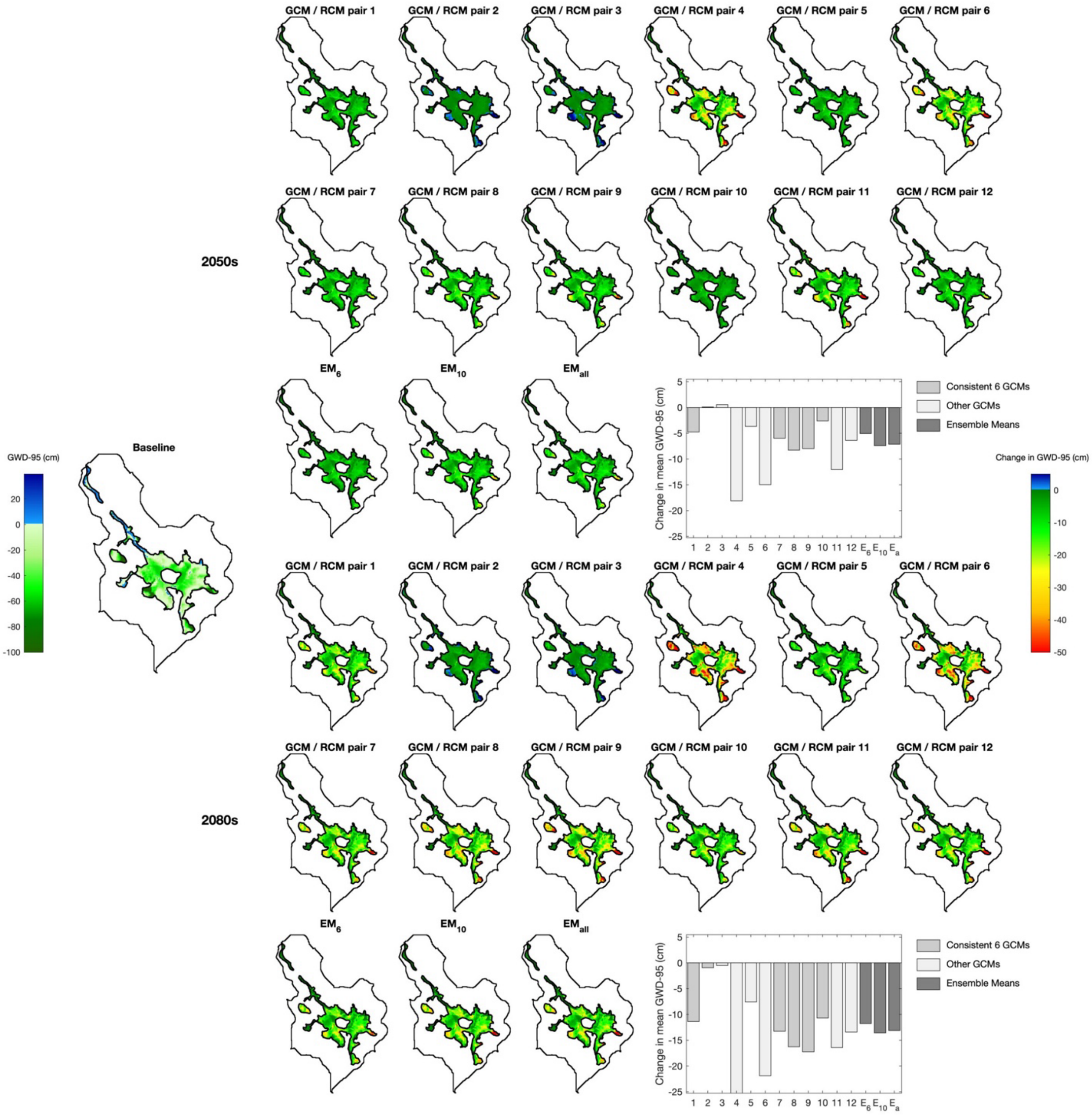

Figure SM4.9. Baseline GWD-95 and changes in GWD-95 across the mire area for RCP8.5 in the 2050s (top) and 2080s (bottom).

SM5. Projected changes in saturated zone-surface seepage

Table SM5.1 summarises the projected changes in the area in which mean September seepage from the saturated zone to the surface exceed the threshold (0.005 mm d^{-1}) that best discriminates between mire and non-mire vegetation for all of the climate change scenarios including the three ensemble means. It also summaries the changes in the mean September seepage within these areas for each scenario. These data are used to summarise the projected changes in saturated zone – surface seepage within Table 6 of the paper.

Table SM5.1. Baseline area (m^2) in which simulated mean September seepage from the saturated zone to the surface exceeds the threshold (0.005 mm d^{-1}) that best discriminates between mire and non-mire vegetation and change in this area (%) for each GCM / RCM pair and the three ensemble means (EM) for the three RCP scenarios in the 2050s and 2080s. Baseline mean September seepage (mm day^{-1}) and change in mean September seepage (%) within these areas.

		GCM / RCM 1	GCM / RCM 2	GCM / RCM 3	GCM / RCM 4	GCM / RCM 5	GCM / RCM 6	GCM / RCM 7	GCM / RCM 8	GCM / RCM 9	GCM / RCM 10	GCM / RCM 11	GCM / RCM 12	EM_6	EM_{10}	EM_{all}
Area within threshold for peat – Baseline: 511,400 m^2																
2050s	RCP2.6	-3.5	2.0	-	-	-5.4	-	2.5	5.5	2.1	0.9	-	3.1	0.3	-	0.04
	RCP4.5	-0.8	1.2	3.9	-14.1	-	-18.1	-6.9	-8.7	-3.2	-3.1	-1.9	-	-4.4	-5.8	-5.8
	RCP8.5	-4.6	1.5	7.3	-24.7	-4.4	-29.0	-4.7	-9.9	-11.5	-4.4	-8.2	-3.7	-6.4	-8.4	-8.0
2080s	RCP2.6	-3.6	-0.9	-	-	0.8	-	0.9	0.8	7.1	0.6	-	1.7	0.4	-	0.5
	RCP4.5	-6.1	-2.3	10.4	-20.7	-	-13.5	-6.4	-6.6	-9.1	-5.9	-9.0	-	-6.1	-7.3	-7.3
	RCP8.5	-6.9	1.8	9.8	-27.5	-3.9	-25.7	-12.8	-24.8	-28.2	-10.4	-8.4	-13.1	-11.7	-13.00	-12.4
Mean September seepage within these areas – Baseline: 3.27 mm day^{-1}																
2050s	RCP2.6	-13.2	4.6	-	-	-9.7	-	1.4	7.6	7.1	5.2	-	3.0	1.7	-	0.3
	RCP4.5	-4.9	-0.1	5.8	-38.6	-	-34.6	-17.6	-19.9	-6.2	-7.4	-13.3	-	-9.8	-14.6	-14.6
	RCP8.5	-12.7	1.4	12.4	-48.6	-11.1	-45.7	-13.4	-22.5	-25.4	-10.9	-26.7	-13.7	-14.8	-20.4	-19.2
2080s	RCP2.6	-11.7	-2.9	-	-	1.8	-	-0.1	1.7	20.9	4.4	-	3.4	2.1	-	2.2
	RCP4.5	-14.8	-2.9	19.0	-39.3	-	-33.1	-16.5	-13.8	-21.3	-15.2	-26.1	-	-14.3	-17.6	-17.6
	RPC8.5	-23.3	3.0	12.7	-57.3	-15.3	-53.7	-32.0	-46.1	-48.9	-27.0	-31.7	-31.6	-29.4	-32.3	-31.2

SM6. Projected changes in the area in which mean groundwater depth (GWD) exceeds the threshold associated with mire vegetation

This section replicates the analysis undertaken within the paper that evaluates changes in the extent of the area in which simulated hydrological conditions exceed established thresholds that can discriminate between mire and non-mire vegetation. Here the threshold is a mean annual groundwater depth of 0.166 m below ground level. This threshold was established by Duranel *et al.* (2021)[1] via the optimisation of a Cohen's kappa agreement function (Congalton, 1991)[2]. The value of kappa for this function (0.841) was slightly lower than that established using the threshold mean September groundwater seepage rate of 0.005 mm d^{-1} (0.845). For this reason, the focus of the analysis as described in the paper, is the climate change-driven changes in September seepage. For completeness, the equivalent changes associated with mean groundwater depth are reported herein.

Table SM6.1 summarises the climate change impacts on the area in which mean groundwater depth exceeds the 0.166 m below ground level threshold and the mean groundwater depth across these cells for each scenario (i.e. different numbers of cells feature in these calculations). This is equivalent to Table 6 in the paper for mean September seepage. The results for all of climate change scenarios are provided in Table SM6.2 (equivalent to Table SM5.1 for mean September seepage).

Table SM6.1. Baseline area (m^2) in which simulated mean groundwater depth (GWD) exceeds the threshold (0.166 m below ground level) that best discriminates between mire and non-mire vegetation; baseline mean GWD (cm above ground) within these areas; maximum, ensemble mean (EM) and minimum changes in these metrics (% for area, cm for mean GWD) across GCM / RCM pairs for each climate change scenario (pairs responsible for the extreme changes); and frequency of positive and negative changes in metrics. Shaded cells indicate reductions in metrics compared to the baseline and where the majority of pairs project declines in the value of the metrics.

	Area within threshold for peat						Mean GWD in peat area					
	2050s			2080s			2050s			2080s		
	RCP 2.6	RCP 4.5	RCP 8.5	RCP 2.6	RCP 4.5	RCP 8.5	RCP 2.6	RCP 4.5	RCP 8.5	RCP 2.6	RCP 4.5	RCP 8.5
	Baseline: 482,300 m^2						Baseline: -2.2 cm					
Max Δ	3.6	2.5	2.5	6.4	3.1	2.4	0.8	0.3	0.3	1.2	0.6	0.1
(GCM/RCM)	(10)	(3)	(3)	(9)	(3)	(3)	(10)	(3)	(3)	(9)	(3)	(3)
EM_6	1.4	-0.5	-1.9	1.6	-1.8	-6.2	0.2	-0.5	-0.9	0.2	-0.9	-2.3
EM_{10}	-	-2.2	-3.8	-	-3.0	-7.9	-	-1.0	-1.4	-	-1.2	-2.7
EM_{all}	1.1	-2.2	-3.3	1.3	-3.0	-7.4	0.1	-1.0	-1.3	0.1	-1.2	-2.6
Min Δ	-2.1	-10.3	-14.7	-3.7	-9.7	-23.2	-0.9	-2.8	-3.6	-1.4	-2.7	-5.4
(GCM/RCM)	(1)	(4)	(4)	(1)	(4)	(4)	(1)	(4)	(4)	(1)	(6)	(4)
No. +ve Δ	5	3	2	7	2	2	4	3	2	5	1	1
No. -ve Δ	3	7	10	1	8	10	4	7	10	3	9	11

Further replicating the approach employed for mean September seepage, Figures SM6.1–SM6.3 map the MIKE SHE grid cells in which the simulated mean groundwater depth exceeds the 0.166 m below ground level threshold. Individual maps are provided for the baseline, each GCM / RCM pair and the available ensemble means in the 2050s and 2080s for the three RCPs. In the case of the baseline, the absolute mean groundwater depth for each cell that exceeds the threshold is indicated whilst for each scenario change in mean

[1] Duranel AJ, Thompson JR, Burningham H, Durepaire P, Garambois S, Wyns R, Cubizolle H (2021) Modelling the hydrological interactions between a fissured granite aquifer and a valley mire in the Massif Central, France. Hydrology and Earth System Sciences 25:291–319. https://doi.org/10.5194/hess-25-291-2021

[2] Congalton RG (1991) A review of assessing the accuracy of classifications of remotely sensed data. Remote Sensing of Environment 37:35–46. https://doi.org/10.1016/0034-4257(91)90048-B

groundwater depth from the baseline is displayed for those cells where the threshold is exceeded. To facilitate comparisons between results for different RCPs, a consistent colour ramp is used for changes in mean groundwater table depth across the three figures. Bar graphs summarise the changes from the baseline in the average mean groundwater depth across the cells that exceed the threshold depth for an individual scenario (i.e. replicating the figures in the bottom part of Table SM6.1). Different shading is used to differentiate the six pairs that provide data for all RCPs, those pairs that do not provide data for all RCPs as well as the ensemble means. Vertical dashed lines within the bar graphs indicate when a particular pair does not provide data for the current RCP. The y-axis ranges for the bar graphs are the same in Figures SM6.1–SM6.3.

Table SM6.1. Baseline area (m^2) in which simulated mean groundwater depth exceeds the threshold (0.166 m below ground level) that best discriminates between mire and non-mire vegetation and change in this area (%) for each GCM / RCM pair and the three ensemble means (EM) for the three RCP scenarios in the 2050s and 2080s. Baseline mean GWD (cm above ground) and change in mean GWD (cm) within these areas. Shaded cells indicate reductions compared to the baseline.

		GCM / RCM 1	GCM / RCM 2	GCM / RCM 3	GCM / RCM 4	GCM / RCM 5	GCM / RCM 6	GCM / RCM 7	GCM / RCM 8	GCM / RCM 9	GCM / RCM 10	GCM / RCM 11	GCM / RCM 12	EM_6	EM_{10}	EM_{all}
Area within threshold for peat – Baseline: 482,300 m^2																
2050s	RCP2.6	-2.1	1.9	-	-	0.8	-	-0.3	1.2	3.17	3.6	-	-0.39	1.4	-	1.1
	RCP4.5	-1.0	1.1	2.5	-10.3	-	-9.4	-2.3	-3.2	-0.10	0.8	-5.7	-	-0.5	-2.2	-2.2
	RCP8.5	-1.4	1.0	2.5	-14.7	-0.1	-12.7	-2.3	-3.9	-5.62	-0.1	-7.9	-3.09	-2.0	-3.8	-3.3
2080s	RCP2.6	-3.7	0.3	-	-	0.9	-	0.4	1.5	6.37	3.1	-	0.08	1.6	-	1.4
	RCP4.5	-2.2	0.5	3.1	-9.7	-	-9.6	-2.00	-0.4	-4.44	-2.5	-7.0	-	-1.8	-3.0	-3.0
	RCP8.5	-4.3	1.9	2.4	-23.2	-2.9	-19.2	-7.5	-12.1	-15.0	-5.41	-10.80	-8.7	-6.2	-8.0	-7.4
Mean GWD within these area – Baseline: -2.2 cm																
2050s	RCP2.6	-0.9	0.3	-	-	-0.2	-	-0.2	0.2	0.7	0.8	-	-0.3	0.2	-	0.1
	RCP4.5	-0.8	0.2	0.3	-2.8	-	-2.7	-1.0	-1.2	-0.2	0.00	-1.9	-	-0.5	-1.0	-1.0
	RCP8.5	-0.9	0.0	0.3	-3.6	-0.5	-3.2	-1.1	-1.6	-1.6	-0.4	-2.3	-1.2	-0.9	-1.4	-1.3
2080s	RCP2.6	-1.4	0.1	-	-	0.04	-	-0.1	0.2	1.2	0.6	-	-0.2	0.2	-	0.1
	RCP4.5	-1.1	-0.01	0.6	-2.6	-	-2.7	-1.0	-0.8	-1.4	-1.1	-2.2	-	-0.9	-1.2	-1.2
	RPC8.5	-2.1	-0.00	0.1	-5.4	-1.4	-4.8	-2.6	-3.5	-3.7	-2.0	-3.2	-2.7	-2.3	-2.7	-2.6

The overall trends associated with changes in the area in which mean annual groundwater tables exceed the mire threshold are very similar to those reported for mean September seepage within the paper. Across the 60 combinations of RCPs, time slices and GCM / RCM pairs, the extent of the area in which the threshold mean groundwater depth is exceeded declines in 39 (65.0%) cases (Table SM6.1). Declines become more numerous with degree of radiative forcing. In the case of RCP2.6, declines in this area are projected by three (37.7%) and one (12.5%) of the eight pairs in the 2050s and 2080s, respectively. The inter-pair range varies by 5.7 (-2.1–3.6%) and 10.0 (-3.7–6.4%) percentage points in the two time slices, respectively. Small (<2%) increases are projected by the two ensemble means. Of the ten pairs for RCP4.5, seven and eight (70% and 80%) project declines in the area in which the threshold water table depth is exceeded. The inter-pair range is equivalent to 12.8 percentage points in both time slices (-10.3–2.5%, 2050s; -9.7–3.1%, 2080s). Small declines (<2% for EM_6, <3% for EM_{all}) are projected by the ensemble means. In the case of RCP8.5, ten of the 12 pairs project declines in area for both time slices with the overall range of changes equivalent to 17.2 and 25.6 percentage points in the 2050s and 2080s (-14.7–2.5% and -23.2–2.4%), respectively. Declines for the ensemble means increase in magnitude compared to those for RCP4.5 (EM_6: 2.0% and 6.2%, <2% larger for the other ensemble means). The inter-pair variations in the areas in which the mean groundwater table depth threshold is exceeded, as well as the pairs responsible for the extremes of the ranges reported above, follows those described in the paper for the area in which the mean September seepage is exceeded (as well as those for groundwater table depth across the

whole mire). For example, in the case of RCP2.6 the largest mean gains in area are projected by either pair 9 or 10 whilst pair 1 projects the largest declines. For both RCP4.5 and RCP8.5 in both time slices the largest increases in area are consistently projected by pair 3 whilst pairs 4 and 6, which do not provide data for RCP2.6 (plus pair 9 for RCP8.5 in the 2080s), are associated with the largest declines.

Further very close similarities between projected changes in the area where mean groundwater table depth exceeds the threshold for mire vegetation and the corresponding changes based on mean September seepage are evident in the spatial patterns of change (Figures SM6.1–SM6.3 for the former and Figures 6–9 in the paper for the latter). Where gains are projected, they are concentrated around the edges of those areas where the threshold is exceeded under baseline conditions although the relatively small extent of these increases means that they are difficult to discern. These same areas are the focus of most declines in the area where water table depth exceeds the threshold when an overall decline in area is projected. As for the changes associated with the seepage threshold, declines below the groundwater level threshold within the main body of the mire are effectively limited to those scenarios with the largest declines in overall area - pairs 4 and 6 for RCP4.5 and RCP8.5 (both time slices) as well as pairs 8 and 9 for RCP8.5 in the 2080s. In these cases, the larger areas where groundwater depth fall below the 0.166 m below ground level threshold are concentrated both upstream and downstream of Puy Rond, in a lobe towards the western mire boundary and in the largest, isolated patch of mire in the west.

Changes in the mean groundwater depth across those MIKE SHE grid cells in which the 0.166 m below ground threshold is exceeded (Table S2.1) also exhibit the same overall trends as those reported in the paper for mean September seepage. Similarly, trends are very similar to those for mean groundwater table depth within the current extent of the mire (Table 5 of the paper) although in most cases the magnitude of changes, especially when there is a decline in the mean, is slightly smaller but then normally by only a few cm. This is the result of the removal of cells around the edge of the mire which experience some of the largest declines in groundwater table depth from the calculation of mean level across those cells where the threshold is now exceed. Declines in mean groundwater depth in those cells exceeding dominate projections (44 or 73.3% of the 60 RCP, time slice and GCM / RCM pair combinations) with declines becoming more common with an increase in the magnitude of radiative forcing and more distance time slice. For example, declines are projected for half of the eight RCP2.6 pairs in both time slices. This increases to seven (70%) and nine (90%) of the ten RCP4.5 pairs in the 2050s and 2080s, respectively, and ten (83.3%, 2050s) and 11 (91.7%, 2080s) of the 12 RCP8.5 pairs. Inter-pair range of change increase as the number of pairs projecting overall declines grows. For example, in the 2050s this range is 1.7 cm (-0.9–0.8 cm) for RCP2.6 and 3.9 cm (-3.6–0.3 cm) for RCP8.5 whilst the corresponding values for the 2080s are 2.6 cm (-1.4–1.2 cm) and 5.5 cm (-5.4–0.1 cm). The same individual pairs account for the extremes of these ranges as those reported above for change in the area in which the water table depth threshold is exceeded. In common with the results for changes in the extent of these areas, the ensemble means project very small increases in mean groundwater level for RCP2.6 (e.g. EM_6 increases are just 0.2 cm in both the 2050s and 2080s, respectively). Declines are projected for higher radiative forcing. In the case of EM_6 in the 2050s these are equivalent to 0.5 cm and 0.9 cm for RCP4.5 and RCP8.5, respectively whist in the 2080s the corresponding declines are 0.9 cm and 2.3 cm. Declines for EM_{10} and EM_{all} are very slightly (<0.5 cm) larger.

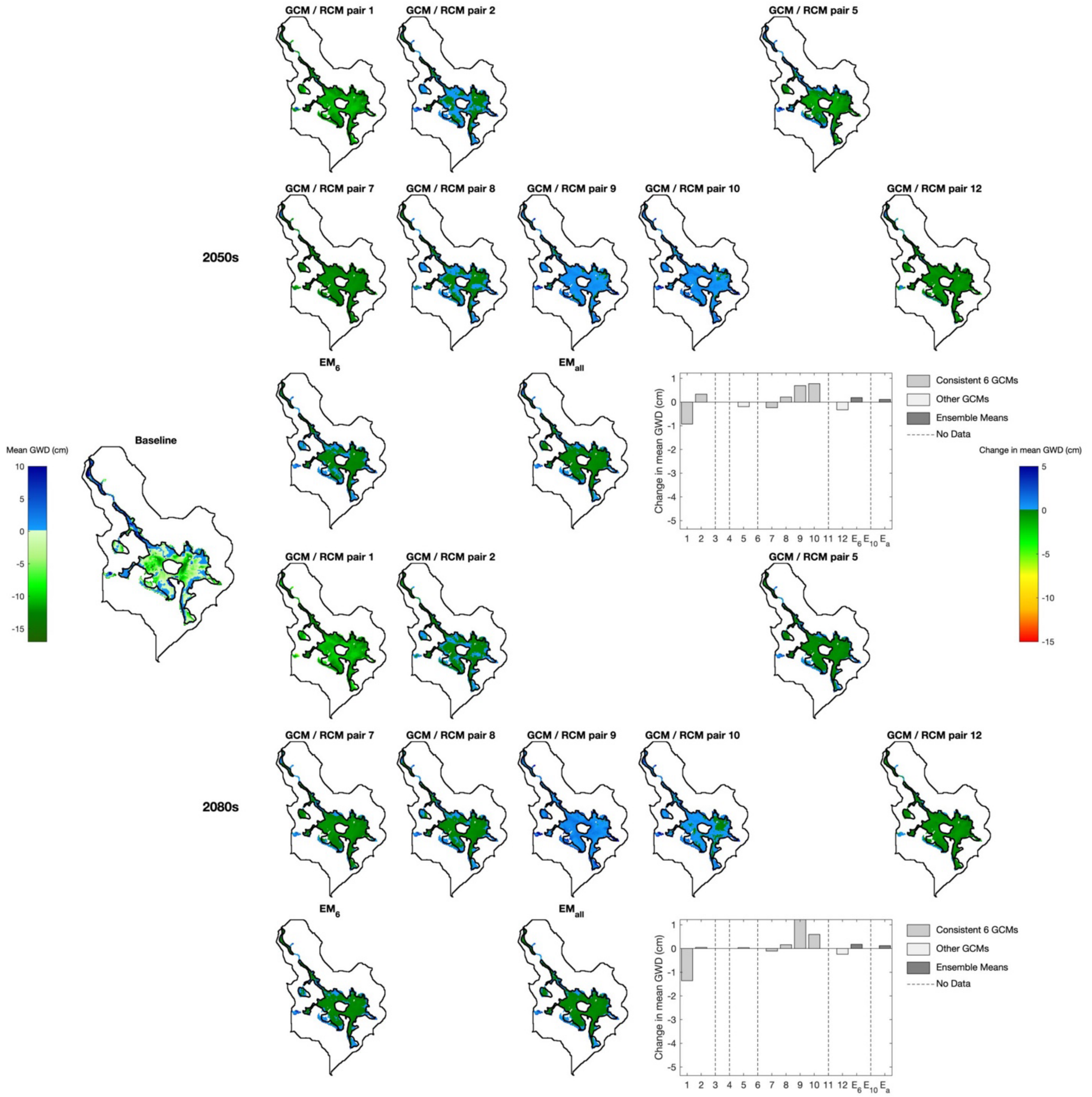

Figure SM6.1. Baseline mean GWD and changes in mean GWD within those MIKE SHE grid cells in which mean GWD exceeds the threshold (0.166 m below ground level) that best discriminates between mire and non-mire vegetation for RCP2.6 in the 2050s (top) and 2080s (bottom).

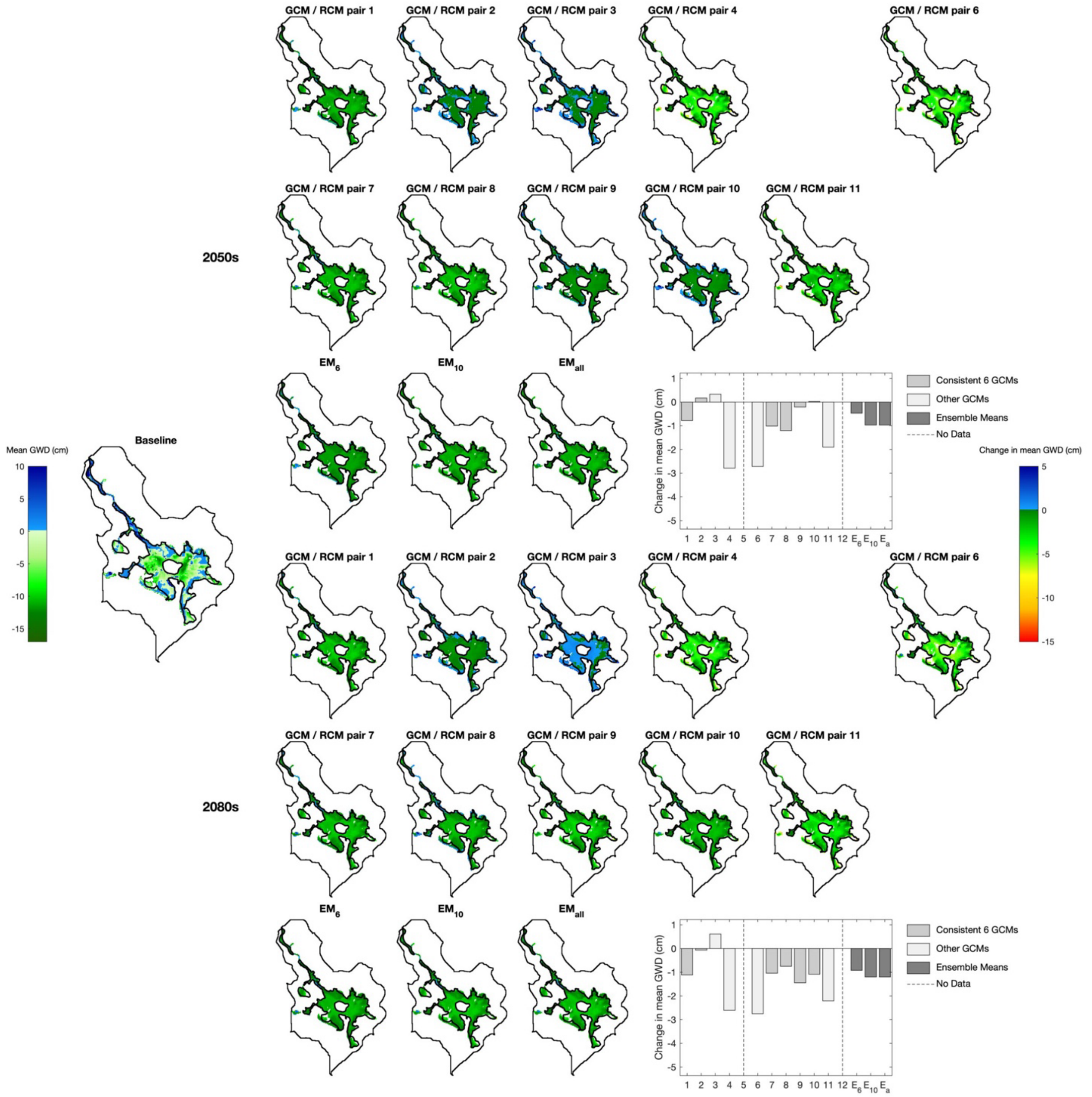

Figure SM6.2. Baseline mean GWD and changes in mean GWD within those MIKE SHE grid cells in which mean GWD exceeds the threshold (0.166 m below ground level) that best discriminates between mire and non-mire vegetation for RCP4.5 in the 2050s (top) and 2080s (bottom).

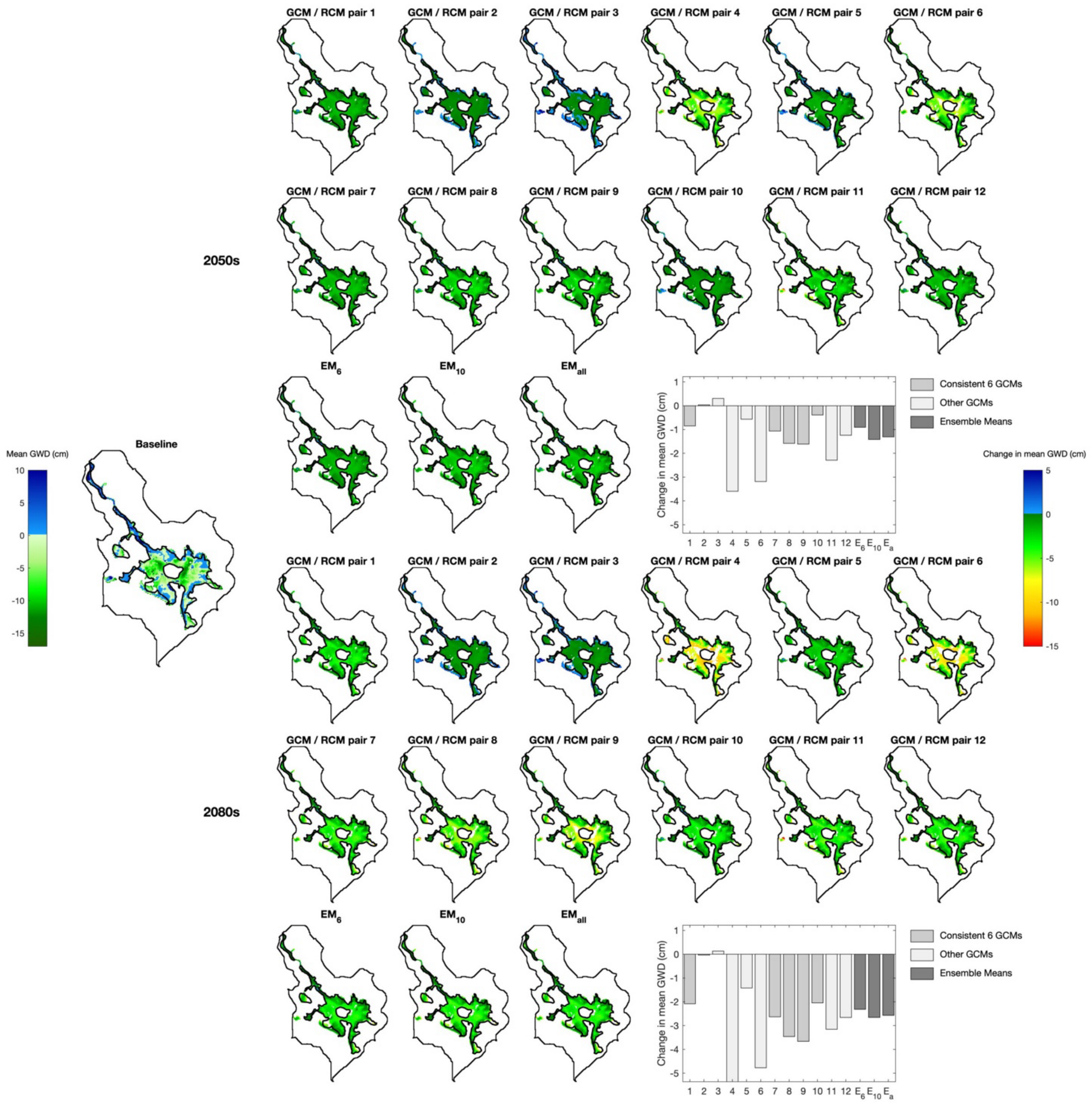

Figure SM6.3. Baseline mean GWD and changes in mean GWD within those MIKE SHE grid cells in which mean GWD exceeds the threshold (0.166 m below ground level) that best discriminates between mire and non-mire vegetation for RCP8.5 in the 2050s (top) and 2080s (bottom).
